# Platinum Meets Pyridine: Affinity Studies of Pyridinecarboxylic Acids and Nicotinamide for Platinum—Based Drugs

**DOI:** 10.3390/ijms262411875

**Published:** 2025-12-09

**Authors:** Beata Szefler, Kamil Szupryczyński, Przemysław Czeleń

**Affiliations:** 1 Department of Physical Chemistry, Faculty of Pharmacy, Collegium Medicum, Nicolaus Copernicus University, Kurpińskiego 5, 85-096 Bydgoszcz, Poland; 2 Doctoral School of Medical and Health Sciences, Faculty of Pharmacy, Collegium Medicum, Nicolaus Copernicus University, Jagiellońska 13, 85-067 Bydgoszcz, Poland

**Keywords:** platinum-based drugs, cisplatin, pyridine, vitamin B3, Gibbs free energy change (ΔG_r_), Density Functional Theory (DFT), UV-Vis spectroscopy

## Abstract

Since 1978, platinum-based drugs have benefited countless cancer patients and come to form the foundation of many cancer pharmacotherapies. These drugs induce apoptosis in cancer cells by forming cross-links between nucleobases in the DNA. Our previous studies have shown that these drugs can also interact with other similar compounds whose structures resemble nucleobases. Therefore, this study analyzed the interactions of Cisplatin, Carboplatin, and Oxaliplatin with Pyridine derivatives (Nicotinic acid, Nicotinamide, Isonicotinic acid, and Picolinic acid). These values were then compared with those for Guanine and Adenine coming from DNA using spectroscopic methods and computational chemistry (B3LYP/6-31G(d,p) and MN15/def2-TZVP methods). Theoretical studies suggest cytostatic affinity, not only for nucleobases but also for Pyridine derivatives. Experimental studies have confirmed these theoretical results.

## 1. Introduction

Cancer remains the world’s leading cause of death, with approximately 20 million people diagnosed and 10 million lives lost each year. Roughly 1 in 5 individuals will develop cancer during their lifetime, and about 1 in 9 men and 1 in 12 women will die from the disease [1]. Nearly 50% of all chemotherapy patients receive platinum-based drugs, which are considered some of the most effective and foundational anticancer agents [2].

**Figure 1:**
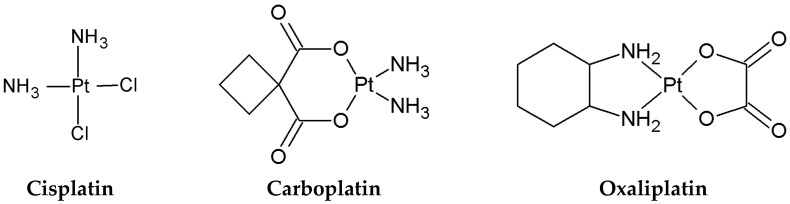
Structure of Cisplatin, Carboplatin, and Oxaliplatin.

Cisplatin is the most popular platinum-based drug and forms the basis for all others. Its anticancer properties were accidentally discovered by Barnett Rosenberg in 1965 [3,4]. This accidental finding led to its rapid introduction to the pharmaceutical market, receiving FDA approval in 1978 [5] and being introduced in Europe a year later [6]. The advent of Cisplatin revolutionized the treatment of solid tumours in combination with other chemotherapy drugs; its cure rate for testicular cancer, for example, exceeds 90% [7]. This success spurred research into further derivatives. Soon after, other drugs were approved, including the well-known Carboplatin (1989) and Oxaliplatin (1996), both of which are globally recognized chemotherapeutic agents [8,9,10,11]. The structures of these medicines are shown in Figure 1. Cisplatin is a potent chemotherapeutic agent with wide clinical use in managing various solid malignancies, including testicular, ovarian, bladder, lung, head, and neck cancers [12,13,14,15,16,17,18].

Carboplatin is less potent than Cisplatin but has reduced systemic toxicity. Consequently, it is broadly used in treating small-cell lung cancer, and cancers of the head, neck, and bladder [19,20,21,22,23,24].

Oxaliplatin was developed to overcome resistance to other platinum agents by operating through a distinct mechanism, specifically the induction of ribosome biogenesis stress [25]. It is primarily employed in the chemotherapy of colorectal cancer [26,27].

### 1.1. Mode of Action

**Figure 2:**
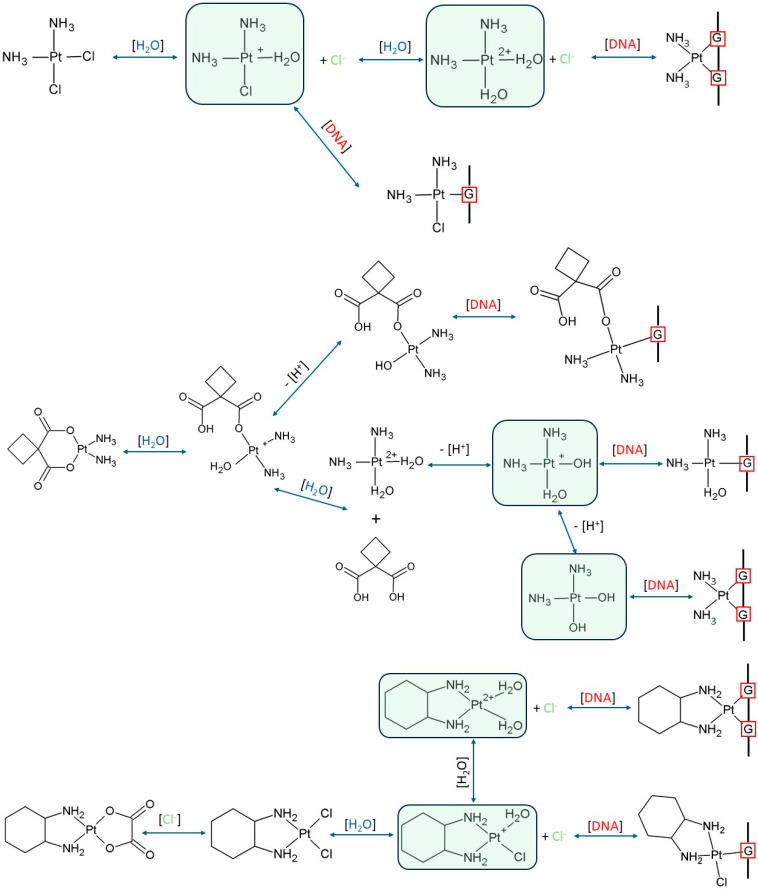
Hydrolysis pathways of Cisplatin, Carboplatin and Oxaliplatin, along with representative examples of their possible interactions with DNA bases. The molecules analyzed in this study are highlighted in green.

Platinum-based drugs are administered intravenously as inactive prodrugs that remain stable in the bloodstream due to the high chloride ion concentration [28]. Upon cellular uptake via various transporters, such as Copper (CTR), Organic Cation (OCT), and Multidrug and Toxin extrusion (MATE) transporters [29,30], they are activated through hydrolysis, which generates active hydrolytic species, as shown in Figure 2 [31,32,33].

**Figure 3:**
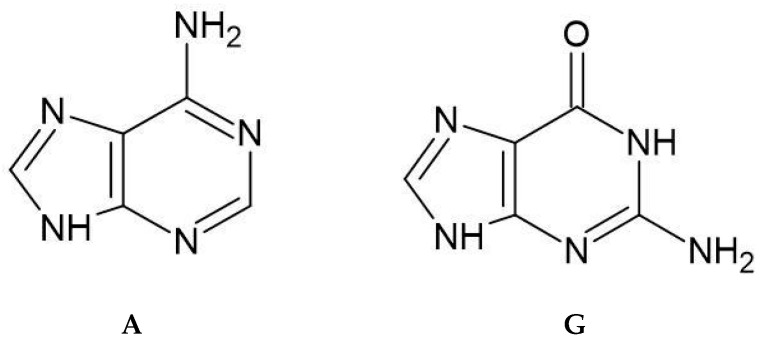
Structures of nucleobases: Adenine (A), Guanine (G).

These active forms of platinum compounds exhibit a strong affinity for nitrogen and sulphur atoms that possess available lone pairs of electrons. These atoms are commonly found in cellular components like the sulfhydryl groups of cysteine residues in proteins and the purine nitrogen atoms of nucleic acids (Guanine and Adenine), whose structures are shown in Figure 3 [34,35,36].

The substitution reactions occurring within the coordination sphere of Pt(II) complexes are classically described by an associative or interchange associative mechanism, as established in numerous kinetic and computational studies [32,33,35,37,38,39,40].

The mechanisms of ligand substitution in square-planar platinum(II) complexes are a classical and well-studied area of coordination chemistry [41]. The fundamental principles governing the kinetics of these processes, including reaction rates and the influence of leaving and entering groups, were defined in pioneering works [41,42] and were comprehensively reviewed in the literature [43,44].

Otto and Elding conducted low-temperature kinetic studies on rapid chloro–halogen substitutions in Pt(II) systems [45], while Samanta et al. investigated the kinetics of Pt(II) substitution by thiol ligands in aqueous solution [46]. Moreover, the structure of the chelating ligand significantly affects the rate of substitution reactions, as demonstrated in studies on cyclometalation [47,48].

The present study builds upon this classical framework to interpret the time-resolved evolution of the UV spectra observed during ligand substitution by pyridine derivatives. However, it should be emphasized that the computational modelling employed herein is not intended to reproduce intermediate transition states.

Cisplatin, the first-generation Pt(II) drug, is a neutral compound containing two labile chloride ligands. Its primary activation mechanism involves the hydrolysis of these chloride ligands in the low chloride environment of the cell to generate the active, electrophilic aqua species, such as [Pt(NH_3_)_2_(H_2_O)Cl]^+^ and the diaqua species [Pt(NH_3_)_2_(H_2_O)_2_]^2+^. These highly reactive intermediates readily bind to nucleobases in DNA, forming cytotoxic adducts responsible for the drug’s therapeutic activity [49,50].

Carboplatin, a second-generation analogue, features a bidentate cyclobutane-1,1-dicarboxylate ligand that is significantly less labile than the chloride ligands of Cisplatin. This enhanced stability results in slower activation, leading to a milder toxicity profile and a longer half-life. Its activation, necessary for generating DNA binding species, proceeds via a substantially slower hydrolysis rate, often involving the displacement of the carboxylate group by water or catalysis by endogenous species such as bicarbonate or carbonate ions [51,52].

For Oxaliplatin and other members of the DACH platinum group, a detailed analysis of hydrolysis and ligand exchange pathways is essential [53,54,55,56]. These studies have clearly identified the resulting species, such as monochloro monooxalato complexes and aqua or chloroaqua cations ([Pt(DACH)(H_2_O)_2_]^2+^, [Pt(DACH)(H_2_O)Cl]^+^), which constitute the active pharmacokinetic forms relevant to the kinetic and computational analyses presented in this work [57].

Once bound to DNA, these compounds form numerous cross-links, primarily within a single DNA strand and, less frequently, between strands. This interaction induces structural distortion and damage to the DNA. If unrepaired by cellular mechanisms, these alterations ultimately trigger apoptosis (programmed cell death) [58].

### 1.2. Pyridine Derivatives

**Figure 4:**
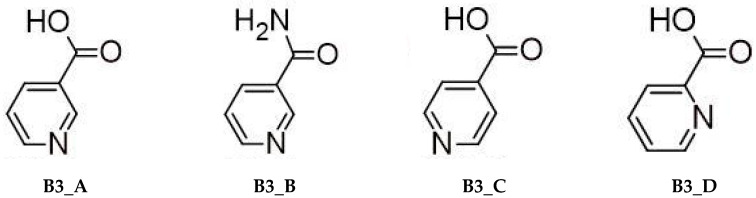
Structures of Pyridine derivatives: Nicotinic acid (B3_A), Nicotinamide (B3_B), Isonicotinic acid (B3_C), and Picolinic acid (B3_D).

Platinum compounds are known to react with structures that resemble nucleobases, particularly those containing nitrogen atoms with lone electron pairs in aromatic rings. Both theoretical and experimental investigations have shown that molecules such as B vitamins (Figure 4) readily interact with platinum-based chemotherapeutic agents (Figure 1) [59,60,61,62,63]. The present study extends this research by examining the affinity of additional Pyridine derivatives:

- Nicotinic acid (3-pyridinecarboxylic acid, B3_A).
- Nicotinamide (pyridine-3-carboxamide, B3_B).
- Isonicotinic acid (pyridine-4-carboxylic, B3_C).
- Picolinic acid (pyridine-2-carboxylic, B3_D).

The compounds studied in the article, Nicotinic acid (B3_A) and Nicotinamide (B3_B), along with its structural isomers Isonicotinic acid (B3_C) and Picolinic acid (B3_D) are derivatives of Pyridine, with Nicotinic acid and Nicotinamide being the two primary vitamers of Vitamin B3 (Niacin).

### 1.3. Significance of Vitamin B3 in the Human Body

Vitamin B3 is an essential nutrient critical for cellular health and metabolism.

- Coenzyme Precursors: Nicotinic acid and Nicotinamide serve as precursors for the vital coenzymes Nicotinamide Adenine Dinucleotide (NAD^+^) and its phosphorylated form, Nicotinamide Adenine Dinucleotide Phosphate (NADP^+^).
- Metabolic Role: These coenzymes are required for over 50 oxidation–reduction (redox) reactions in the body. NAD^+^ is primarily involved in catabolic processes that generate energy from carbohydrates, fats, and proteins, while NADP^+^ is essential for anabolic reactions, such as the synthesis of fatty acids and cholesterol.
- Cellular Function: The NAD^+^ system is also a substrate for non-redox reactions, including those involved in DNA repair, cell signalling, and protein modification, which are crucial for maintaining genome stability.
- Deficiency: A severe deficiency of Vitamin B3 causes pellagra, a disease characterized by the “three Ds”: dermatitis, diarrhea, and dementia.

### 1.4. Pharmacological Importance of Vitamin B3

The two main vitamers, Nicotinic acid and Nicotinamide, have distinct therapeutic uses at pharmacological doses:

- Nicotinic Acid (Niacin): It is a long-established prescription medication primarily used to treat dyslipidaemia (high cholesterol and triglycerides). It significantly lowers LDL (“bad”) cholesterol and triglycerides while increasing HDL (“good”) cholesterol.
- Nicotinamide (Niacinamide): This form is preferred for treating niacin deficiency (pellagra) as it avoids the severe flushing side effect caused by Nicotinic acid. Pharmacologically, it is used in dermatology for conditions like acne, rosacea, and has been shown to reduce the incidence of non-melanoma skin cancer (NMSC) in high-risk patients by enhancing DNA repair. It may also be used adjunctively for osteoarthritis and diabetes.

The crucial biological function of Vitamin B3 (Niacin), primarily in the form of Nicotinic acid and Nicotinamide, stems from its role as a precursor to the coenzymes NAD^+^ and NADP^+^, which are indispensable for redox reactions, DNA repair, and overall cellular energy metabolism. However, it is the structural characteristic of these compounds specifically, their pyridine ring that holds significant implications in the context of cancer pharmacotherapy. Given that the mechanism of action of platinum-based chemotherapy drugs (such as Cisplatin, Carboplatin and Oxaliplatin) relies on their affinity for nucleobases (like Guanine and Adenine) in DNA, the structural resemblance between the pyridine ring of the B3 vitamers and these nucleobases is key. This structural similarity suggests a potential for competitive coordination and complex formation, leading to the hypothesis tested in this study: that the described Nicotinic acid and Nicotinamide compounds may actively interfere with or influence the action of platinum chemotherapeutics by forming complexes with them.

Both theoretical and experimental studies indicate that molecules such as B-group vitamins containing a pyridine ring can interact with platinum-based drugs. This structural similarity to the natural targets of platinum, the purine nucleobases in DNA (Guanine and Adenine), what is crucial, since the cytotoxic mechanism of these drugs relies on the formation of DNA adducts, as described in detail in the literature [43,50,57]. Our hypothesis assumes that this structural analogy leads to competitive coordination.

**Figure 5:**
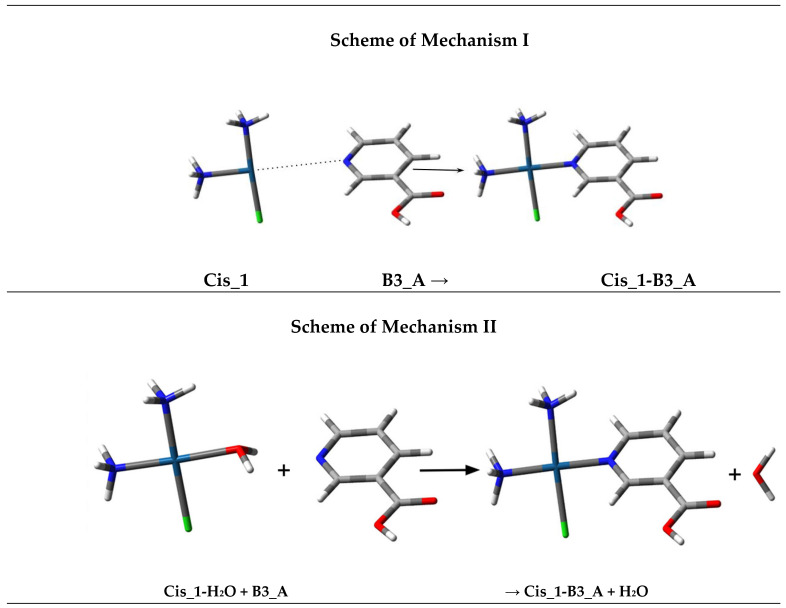

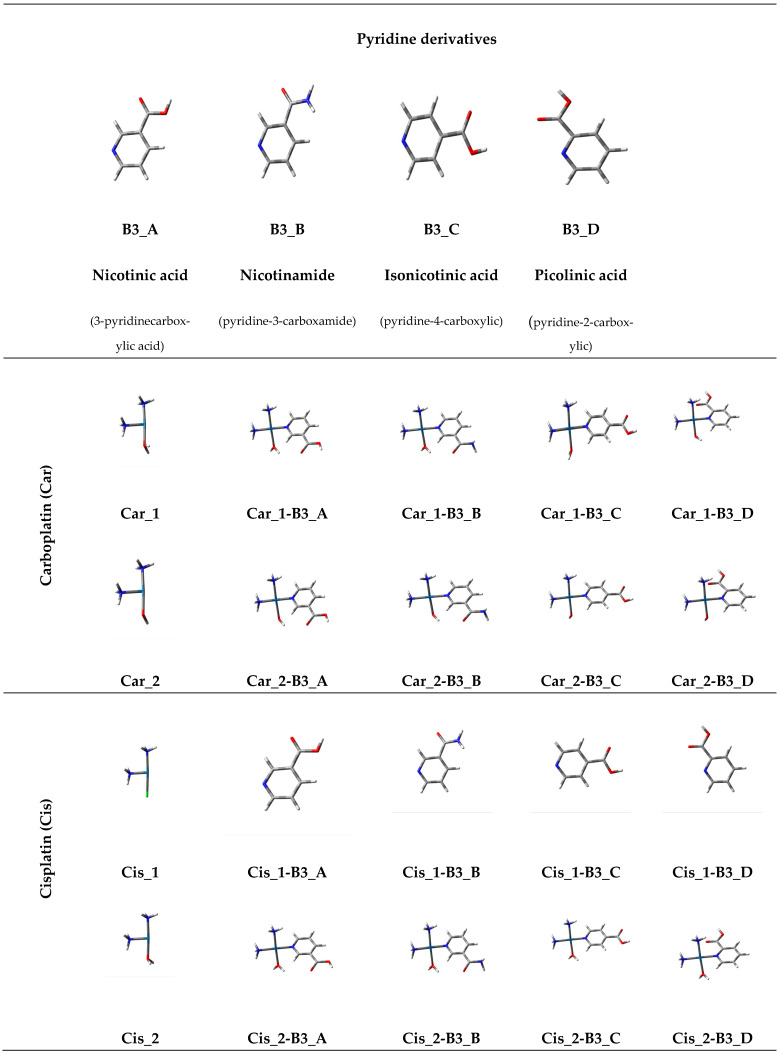

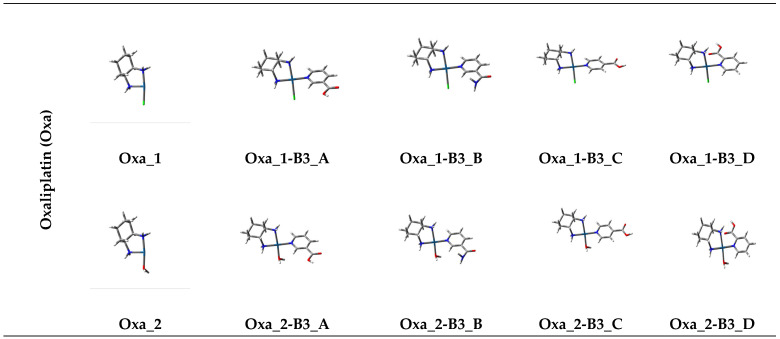
Scheme of the complex formation between first and second products of hydrolysis of Cisplatin, Carboplatin or Oxaliplatin with studied Pyridine derivatives, such as Nicotinic acid (B3_A), Nicotinamide (B3_B), Isonicotinic acid (B3_C), and Picolinic acid (B3_D), as well as Nucleobases, such as Adenine (A) and Guanine (G) (Mechanism I), and scheme of the complex formation involving a water molecule in tetracoordinated Pt complexes with studied structures (Mechanism II).

The present study investigates the molecular interactions between several Pyridine derivatives, which include the essential B vitamin forms Nicotinic acid and Nicotinamide, and the core group of platinum-based chemotherapeutic agents, namely Cisplatin, Carboplatin and Oxaliplatin. These platinum drugs are known to be highly effective against various cancers, inducing apoptosis primarily by forming cross-links with nucleobases in DNA. However, based on the principle that platinum compounds can also interact with other molecules structurally similar to nucleobases (Figure 4 and Figure 5), particularly those containing lone electron pairs on aromatic nitrogen atoms, both theoretical and experimental investigations confirm that these B vitamin derivatives possess a significant affinity for the platinum-based chemotherapeutics and form stable complexes with them.

## 2. Results and Discussion

### 2.1. Gibbs Free Energy Changes (ΔG_rs_)

Table 1 and Table 2 present the calculated Gibbs free energy changes (ΔG_rs_) for the complex formation between the various platinum species and the Pyridine derivatives (Figure 5), as well as the nucleobases Adenine (A) and Guanine (G), using both the B3LYP/6-31G(d,p)/LANL2DZ and MN15/def2-TZV computational methods.

The negative ΔG_rs_ values in all tables (Mechanism I) indicate that the complex formation between the hydrolysed platinum forms and the tested compounds is thermodynamically favourable (spontaneous).

However, the computational data clearly demonstrate that Mechanism I (direct ligand substitution from the hypothetical T-shaped or three-coordinate Pt(II) fragment) consistently yields strongly negative ΔG_r_ values for all systems. These values probably predominantly reflect the energy released upon breaking a coordinated water or chloride ligand in a low-coordination fragment structures. In contrast, Mechanism II, which evaluates the reaction: Pt(H_2_O)(L*) + B3 ligand → Pt(B3 ligand)(L*) + H_2_O, probably more accurately models aqueous reactivity of square-planar Pt(II) complexes.

The water substitution reactions corresponding to Mechanism II, maintain the tetracoordinate geometry of Pt(II) and directly represent the competition between a coordinated H_2_O molecule and an incoming ligand.

#### 2.1.1. Comparison of Computational Methods (B3LYP vs. MN15)

**Figure 6:**
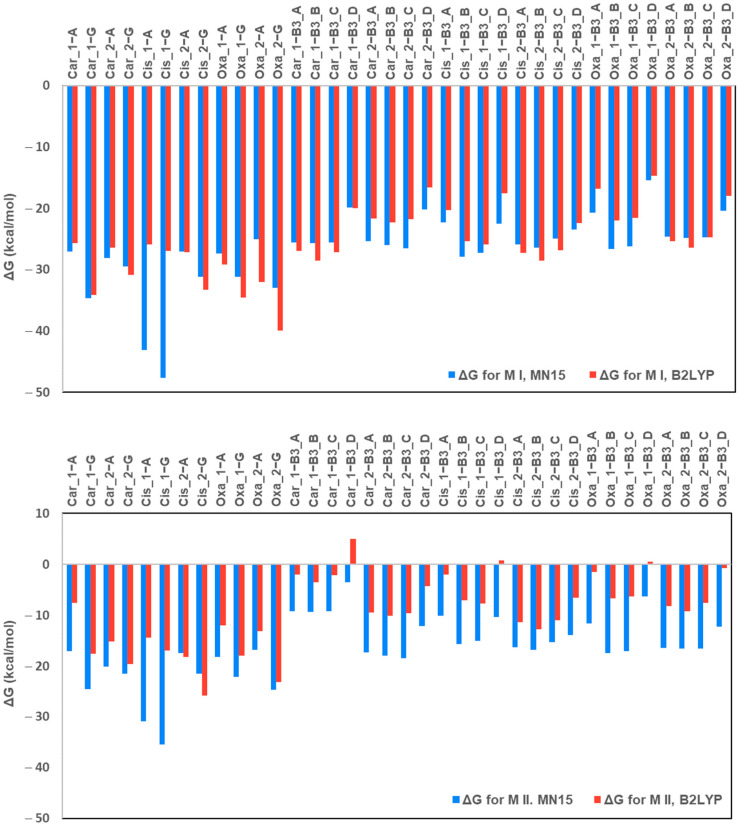
Calculated values of the Gibbs free energy changes (ΔG_rs_) for the complexation reactions between the primary and secondary hydrolysis products of the platinum-based drugs (Mechanism I (M I)) and for the complexation reactions with the water substitution reaction in tetracoordinated Pt complexes (Mechanism II (M II)), specifically Cisplatin (Cis_1 and Cis_2), Carboplatin (Car_1 and Car_2), and Oxaliplatin (Oxa_1 and Oxa_2) and the tested ligands. The ligands include: Pyridine derivatives: Nicotinic acid (B3_A), Nicotinamide (B3_B), Isonicotinic acid (B3_C), and Picolinic acid (B3_D); Nucleobases: Adenine (A) and Guanine (G). Comparison of two computational methods, the B3LYP/6-31G(d,p)/LANL2DZ and the MN15/def2–TZV.

The ΔG_rs_ values show differences between the B3LYP and MN15 methods (Figure 6).

Both computational approaches (B3LYP/6-31G(d,p)/LANL2DZ and MN15/def2-TZVP) yield consistently negative Gibbs free energy changes for the water–ligand substitution reactions described by Mechanism II, indicating that the exchange of coordinated water by the tested ligands is thermodynamically favourable. However, clear quantitative differences arise between the methods.

The MN15 functional predicts systematically more negative ΔG_r_ values for nucleobase substitution, particularly for Guanine indicating a stronger thermodynamic preference of Pt(II) species for biologically relevant purines. In contrast, B3LYP yields ΔG_r_ values for pyridine derivatives that are closer in magnitude to those obtained for Adenine, occasionally suggesting comparable affinity. Thus, MN15 provides a sharper distinction between the nucleobases and the pyridine ligands, whereas B3LYP tends to underestimate this contrast.

Despite these quantitative differences, both methods produce identical qualitative trends:

(i) All water substitution reactions remain thermodynamically favourable;
(ii) Guanine consistently exhibits the strongest affinity among all ligands;
(iii) Nicotinamide (B3_B) is the most favourable pyridine derivative; and
(iv) Picolinic acid (B3_D) is the least favourable ligand.

These results confirm that MN15 and B3LYP differ mainly in the absolute magnitude of predicted ΔGr values, while their relative ordering of ligands is fully consistent across Mechanism II reactions.

#### 2.1.2. Affinity Trend for Pyridine Derivatives

Evaluation of the water–ligand substitution reactions (Mechanism II) reveals a clear thermodynamic ranking among the pyridine derivatives, strongly dependent on the position and nature of the substituent on the aromatic ring.

Across all Pt(II) species and both computational methods, Nicotinamide (B3_B) exhibits the most favourable Gibbs free energy of substitution, indicating the highest thermodynamic affinity. This behaviour is fully consistent with its lower HOMO–LUMO energy gap and higher electronic softness (Table 3), which facilitate more effective electron donation to the electrophilic Pt(II) centre (Figure S30, Table S1).

Nicotinic acid (B3_A) and Isonicotinic acid (B3_C) show intermediate affinity values, with small variations depending on the hydrolysis state of the drug. Their comparable ΔGr values reflect similar electronic characteristics and the absence of chelation-driven geometric effects.

In contrast, Picolinic acid (B3_D) consistently displays the least favourable substitution free energies among the pyridine derivatives. Although its 2-carboxylate position enables rapid chelation and fast complex formation (as observed experimentally), its electronic “hardness” and large HOMO–LUMO gap result in a thermodynamically weaker Pt–ligand bond. Mechanism II therefore confirms that B3_D is a kinetically privileged ligand but a thermodynamically disfavoured one. Overall, the thermodynamic affinity trend among the pyridine derivatives is:

B3_B (Nicotinamide) > B3_A ≈ B3_C >> B3_D (Picolinic acid), and this ranking is fully consistent with the electronic descriptors provided in Table 3.

### 2.2. HOMO–LUMO Energies and Chemical Properties of Studied Molecules

The chemical properties of the free ligands, platinum hydrolysis products, and their complexes were calculated using Density Functional Theory (DFT) at two levels: B3LYP/6-31G(d,p)/LANL2DZ (Tables S1 and S2) and MN15/def2-TZV (Tables S1 and S2). The parameters studied include HOMO and LUMO energies, energy gap (ΔE_gap_), absolute electronegativity (χ), Chemical potential (μ), absolute hardness (η), absolute softness (σ), global electrophilicity index (ω), global softness (S), and maximum additional electronic charge (ΔN_max_), (Figure S30, Tables S1 and S2). General trends in respect of chemical properties of studied molecules are presented in Table 3.

#### 2.2.1. Properties of Complexes and Method Comparison

- Reactivity: The smaller energy gap (ΔE_gap_) and higher global electrophilicity index (ω) of the platinum hydrolysis products (Pt-drugs) confirm their nature as highly reactive electrophiles, ready to accept electrons from the ligands.
- Complex Stability: Upon complexation with the ligands, the energy gap (ΔE_gap_) generally increases, suggesting the resulting complex is more stable and less reactive than the initial Pt-drug fragment.
- Method Consistency: The overall electronic property trends calculated by the MN15 method were consistent with those from the B3LYP method, although minor numerical differences in the values were observed.

#### 2.2.2. Correlation with Gibbs Free Energy (ΔG_rs_)

The HOMO/LUMO energy values and derived parameters correlate with the tendency of the reaction to occur, which is quantitatively measured by the change in Gibbs Free Energy (ΔG_rs_).

#### 2.2.3. Thermodynamic Spontaneity

- Affinity: All hydrolysed platinum forms (Cis_1, Cis_2, Car_1, Car_2, Oxa_1, Oxa_2) exhibit an affinity for all tested compounds (Pyridine derivatives and nucleobases).
- Spontaneity: This affinity is confirmed by the negative ΔG_rs_ values across all complexation reactions calculated by both the B3LYP and MN15 methods, indicating that complex formation is thermodynamically spontaneous.

#### 2.2.4. Correlation of Electronic Structure with Affinity (ΔG_rs_)

The highest affinity for a ligand (most negative ΔG_rs_) should correlate with a ligand’s superior electron-donating capability (highest HOMO energy, lowest χ) and the Pt-drug’s superior electron-accepting capability (highest ω, lowest ΔE_gap_).

- Overall Highest Affinity: Guanine (G) exhibits the highest affinity among all tested compounds, consistent with its role as the most potent nucleophile, possessing the highest E_HOMO_ and lowest absolute electronegativity (χ) among the free ligands.
- Highest Affinity Pyridine Derivative: Among the Pyridine derivatives, the Pt-drugs show the highest overall affinity for Nicotinamide (B3_B).
- Lowest Affinity Pyridine Derivative: Picolinic acid (B3_D) consistently displayed the lowest affinity among the Pyridine derivatives across nearly all platinum species and methods. The study suggests this lower affinity is due to the unfavourable position of the carboxylic substitution (at position 2).
- Method-Dependent Affinity Trend: The MN15 method generally showed higher affinity for Adenine (A) compared to the Pyridine derivatives, while the B3LYP method showed similar affinities between Adenine and the Pyridine derivatives.

### 2.3. UVVIS UV-Vis Spectroscopy Analysis and Correlation with Computational Data

#### 2.3.1. Analysis of Experimental UV-Vis Results, the Time-Resolved Evolution of the UV Spectra and Thermodynamic Disparity

The time-dependent UV-Vis spectroscopic analysis (Figure 7, Figure 8 and Figure 9 and Figures S4–S24) provides crucial experimental evidence for the mechanism and qualitative kinetics as the time-resolved evolution of the UV spectra of complexation between platinum drugs (Carboplatin, Cisplatin, Oxaliplatin) and the studied ligands (Pyridine derivatives and nucleobases).

Although the obtained UV–Vis spectra clearly demonstrate time-dependent complexation, it should be emphasized that these measurements primarily provide qualitative kinetic information rather than quantitative rate constants. The UV–Vis bands of the free ligands and their platinum complexes exhibit significant spectral overlap, particularly in the 220–275 nm region, where both species absorb strongly. This overlap hampers accurate deconvolution of individual contributions, making it difficult to reliably extract reaction rate constants or equilibrium constants from the absorbance–time profiles.

Therefore, in this study, UV–Vis spectroscopy was used to monitor the overall progression of complexation reactions and to compare relative rates of complex formation across different ligands. The observed kinetic behaviour as the time-resolved evolution of the UV spectra (e.g., the rapid formation of Picolinic acid complexes vs. slower evolution in Nicotinic acid systems) should be interpreted as semi-quantitative trends rather than precise kinetic constants.

This limitation is intrinsic to UV–Vis analysis of overlapping electronic transitions in the low-UV range.

##### The Time-Resolved Evolution of UV Spectra as Hydrolysis and Complexation Kinetics

**Figure 7:**
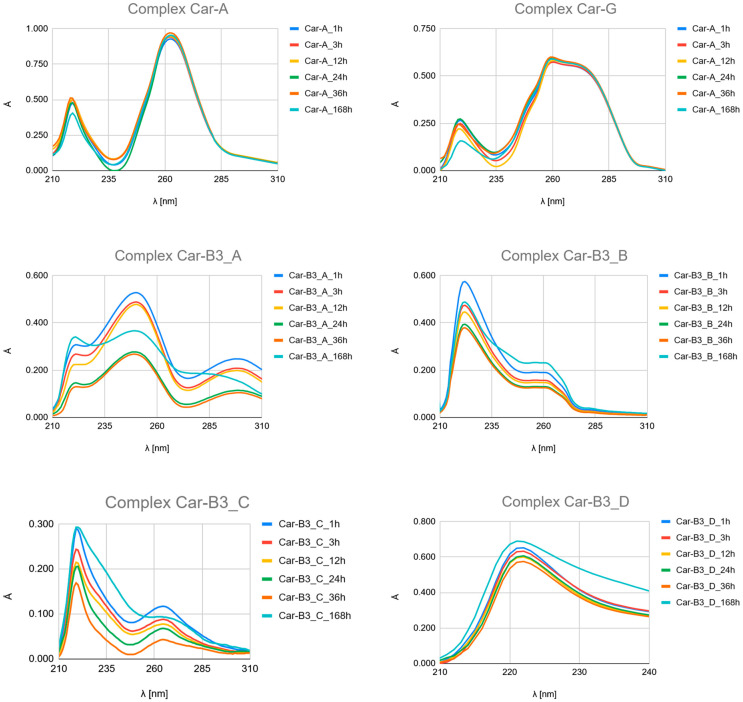
UV–Vis spectra of a mixed solution of Carboplatin (Car) with nucleobases Adenine (A) and Guanine (G), and with Nicotinic acid (3-pyridinecarboxylic acid, B3_A), Nicotinamide (pyridine-3-carboxamide, B3_B), Isonicotinic acid (pyridine-4-carboxylic, B3_C) and Picolinic acid (pyridine-2-carboxylic, B3_D) recorded after 1, 3, 12, 24, 36, and 168 h of incubation at 37 °C.

**Figure 8:**
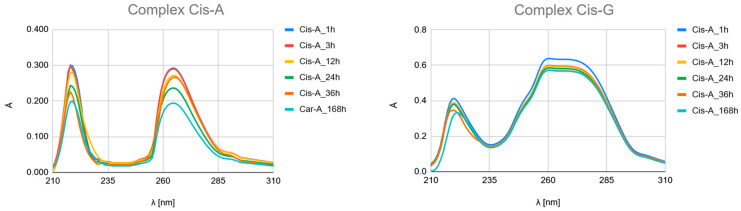

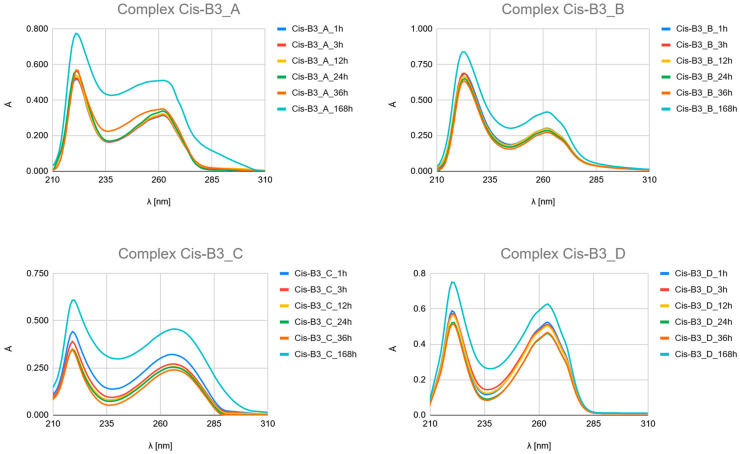
UV–Vis spectra of a mixed solution of Cisplatin (Cis) with nucleobases Adenine (A) and Guanine (G), and with Nicotinic acid (3-pyridinecarboxylic acid, B3_A), Nicotinamide (pyridine-3-carboxamide, B3_B), Isonicotinic acid (pyridine-4-carboxylic, B3_C) and Picolinic acid (pyridine-2-carboxylic, B3_D) recorded after 1, 3, 12, 24, 36, and 168 h of incubation at 37 °C.

**Figure 9:**
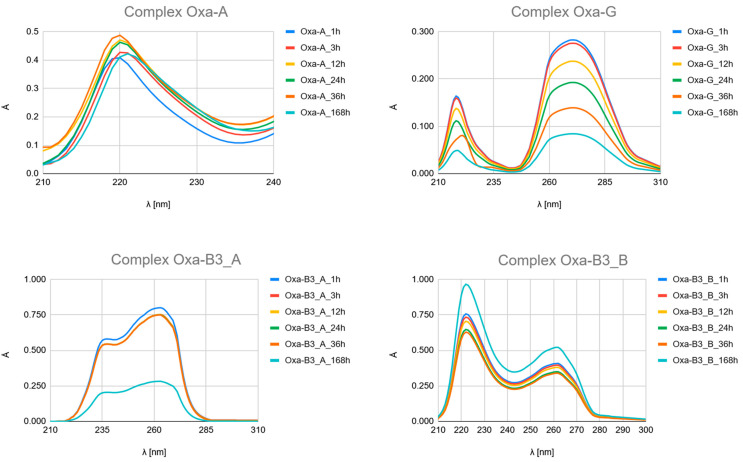

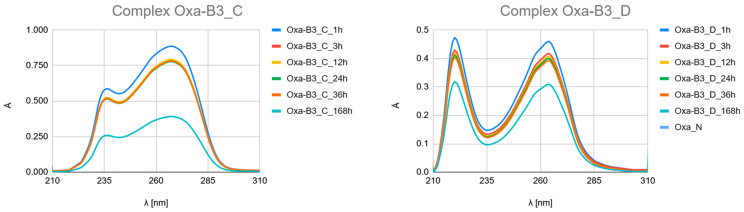
UV–Vis spectra of a mixed solution of Oxaliplatin (Oxa) with nucleobases Adenine (A) and Guanine (G), and with Nicotinic acid (3-pyridinecarboxylic acid, B3_A), Nicotinamide (pyridine-3-carboxamide, B3_B), Isonicotinic acid (pyridine-4-carboxylic, B3_C) and Picolinic acid (pyridine-2-carboxylic, B3_D) recorded after 1, 3, 12, 24, 36, and 168 h of incubation at 37 °C.

- Carboplatin Hydrolysis (Figure 7 and Figures S6–S10): The observed marked decrease in absorbance peaks (≈220 nm and ≈275 nm) over 168 h is consistent with the slow hydrolysis of the parent drug into less chromophoric aquated species (Car_1 and Car_2), which are the actual reactive intermediates.
- Ligand Binding (Figure 7, Figure 8 and Figure 9 and Figures S4–S24): All spectra confirm the time-dependent formation of new complexes, supporting the multi-step mechanism: hydrolysis followed by ligand substitution.
- Kinetic Outlier (Picolinic Acid, B3_D): Picolinic acid (B3_D) exhibits the most significant spectral evolution, characterized by a substantial and rapid increase in absorbance (e.g., around 220 nm and 265 nm) over time (Figure 7, Figure 8 and Figure 9, Figures S10, S17 and S24). This pronounced change is indicative of a high kinetic preference and rapid formation of a stable, chromophoric chelate complex. This kinetic superiority, however, contrasts sharply with the computational thermodynamic data.

##### Correlation with Computational Data (ΔG_rs_, and HOMO-LUMO Gap)

The comparison of the experimental time-resolved evolution of UV spectra with computational results (Table 4) reveals a fundamental interplay between reaction speed and final product stability (thermodynamics).

##### Experimental Time-Resolved Evolution of UV Spectra vs. Computational Thermodynamics and Enthalpy

The time-resolved evolution of UV spectra data for B3_D stands in direct contrast to the computational thermodynamic data (ΔG_rs_).
**Ligand****UV-Vis Evolution****ΔGrs (Thermodynamic Stability)****Conclusion****Picolinic Acid (B3_D)**Fastest and most complete reaction.Lowest affinity (least negative ΔG_rs_ among pyridines).The chelate effect provides a kinetic advantage, enabling a faster reaction, but the resulting complex is the least thermodynamically stable.**Nicotinamide (B3_B)**Fast initial change, stable final complex.Highest affinity (most negative ΔG_rs_ among pyridines).The complex is both kinetically accessible and thermodynamically favoured, likely due to optimal electronic fit and bond strength.


This highlights that the 2-carboxylic group of Picolinic acid provides a low-energy pathway for the transition state, leading to rapid substitution. However, the five-membered chelate ring is entropically or enthalpically less stable than the non-chelate complexes formed by the 3- and 4-substituted isomers, resulting in lower overall thermodynamic affinity.

##### Correlation with Electronic Properties (HOMO-LUMO Gap)

The HOMO-LUMO gap (ΔE_gap_ from Tables S1 and S2) correlates strongly with the calculated thermodynamic affinity (ΔG_rs_) and supports the principle of soft–soft interactions.

- Nicotinamide (B3_B): Exhibits the lowest ΔE_gap_ (5.8 eV) among the Pyridine derivatives, indicating it is the electronically ‘softest’ ligand. This ‘softness’ aligns perfectly with its highest observed thermodynamic affinity (ΔG_rs_) for the soft Platinum(II) centre.
- Picolinic Acid (B3_D): Possesses the largest ΔE_gap_ (6.2 eV), confirming its relative electronic “hardness.” This electronic property justifies its lowest calculated thermodynamic stability.

##### Comparison with Calculated UV-Vis λ_max_

The calculated UV-Vis wavelengths (λ_max_) from Time-Dependent DFT (TD-DFT) methods (Figures S25–S29, Table 4) generally support the existence of intense electronic transitions (e.g., π → π∗ or charge-transfer) in the UV region for the formed complexes. Although DFT often deviates from experimental values, the theoretical λ_max_ values confirm the highly chromophoric nature of the final products, which is manifested as the large intensity changes observed in the experimental UV-Vis spectra.

The computational results, based on the Gibbs free energy changes (ΔG_rs_) from Table 1 and Table 2, lead to the following conclusions: All hydrolysed platinum forms exhibit affinity for the studied compounds (Pyridine derivatives and nucleobases), as evidenced by the negative ΔG_rs_ values in all calculations, which indicates that complex formation is thermodynamically spontaneous. There are notable differences in affinity depending on the computational method used, reflecting inherent differences in how the B3LYP and MN15 methods calculate these energy values. The MN15 method generally showed higher affinity for Adenine compared to the Pyridine derivatives, while the B3LYP method showed similar affinities. A significant difference in affinity was observed based on the position of the carboxylic substitution. Picolinic acid (B3_D), with its 2-carboxylic group, consistently displayed the lowest affinity among the Pyridine derivatives across nearly all platinum species and methods. Overall, Guanine exhibits the highest affinity among all tested compounds. Among the Pyridine derivatives, the platinum-based drugs show the highest overall affinity for Nicotinamide (B3_B).

#### 2.3.2. New Findings and Conclusions from Spectroscopic and Correlative Analysis

The time-resolved evolution of UV spectra as hydrolysis and Complexation Kinetics study experimentally confirms the time-dependent complexation, corroborating the hydrolysis/substitution mechanism and supporting the theoretical model used in the DFT calculations.

A key mechanistic insight is the apparent discordance between qualitative kinetics as the time-resolved evolution of the UV spectra and thermodynamics: while Picolinic acid (B3_D) is found to be the least thermodynamically stable complex (lowest affinity, highest ΔG_rs_), its complexation reaction is observed to be the most rapid and visually extensive in the UV-Vis spectral analysis. This suggests that the chelate effect arising from the 2-carboxylic substitution provides a significant kinetic advantage, enabling faster substitution at the platinum centre, despite leading to a less stable final product.

Furthermore, a direct correlation is established between the electronic properties of the ligands and the stability of the final complexes. Nicotinamide (B3_B) exhibits the lowest HOMO-LUMO energy gap (ΔE_gap_ = 5.8 eV) among the Pyridine derivatives, indicating it is the electronically ‘softest’ ligand, which rationalizes its highest observed thermodynamic affinity via favourable soft–soft interactions with Platinum(II). Conversely, the largest ΔE_gap_ for Picolinic acid (B3_D) (ΔE_gap_ = 6.2 eV) confirms its electronic “hardness” and low thermodynamic stability.

These findings highlight that while the electronic structure dictates the final thermodynamic stability (ΔG_rs_/affinity), the structural configuration (chelation) of the ligand can dramatically influence the reaction kinetics (UV-Vis profile), providing a comprehensive understanding of the ligand substitution process.

## 3. Materials and Methods

### 3.1. Theoretical Study

Density Functional Theory (DFT) [74,75] was used to construct theoretical models to describe the geometric structures, energetic stability, and electronic properties of the compounds studied. Molecular models were prepared using GaussView 6.0.16 (Wallingford, CT, USA), and all quantum-chemical calculations were performed with the Gaussian 16 Rev. C.01 package (Wallingford, CT, USA) [76]. Geometry optimizations were carried out using two theoretical approaches to locate energy minima:

1. B3LYP/6-31G(d,p) [77,78], where platinum atoms were treated with the LanL2DZ [79] basis set, which incorporates relativistic effective core potentials suitable for heavy elements. The B3LYP functional is a hybrid approach recognized for its broad applicability and has been validated in studies of cisplatin–nucleobase interactions.
2. MN15/def2-TZVP. The MN15 functional is a newer hybrid functional optimized for electronically complex and larger systems, offering enhanced accuracy and reduced error rates [80].

Harmonic vibrational frequency analyses were performed to determine Zero Point Energies (ZPEs). Solvent effects (aqueous environment) were modelled using the Polarizable Continuum Model (IEF-PCM) with Bondi radii [81].

Spectroscopic characteristics were determined using the PBE0 hybrid functional, which is well known for providing accurate predictions of electronic excitation spectra [82]. The HOMO and LUMO energy levels, key parameters for interpreting electron distribution [67,83,84] and possible electronic transitions were obtained using the same DFT methodologies (B3LYP/6-31G(d,p)/LANL2DZ and MN15/def2-TZVP). These frontier orbital energies are essential, as they directly define a molecule’s electron-donating ability (HOMO) and electron-accepting capacity (LUMO), thus governing its overall chemical reactivity.

Based on the computed HOMO and LUMO energies, several chemical reactivity descriptors were derived. These quantitative parameters provide detailed insight into the molecule’s chemical nature:

- Energy Gap (ΔE_gap_): the difference between LUMO and HOMO energies (LUMO-HOMO), serving as an indicator of molecular stability and reactivity. Generally, a smaller gap corresponds to higher reactivity and more facile electronic transitions.

Absolute Electronegativity (χ): expresses the ability of an atom or group to attract electrons; it is defined as the negative of the chemical potential.

- Chemical Potential (μ): describes the tendency of electrons to escape from a molecular system.
- Absolute Hardness (η): reflects the resistance of a molecule to charge transfer or distortion of its electron cloud; higher values correspond to harder (less reactive) species.
- Absolute Softness (σ): the reciprocal of hardness, indicating how easily charge transfer can occur.
- Global Electrophilicity (ω): quantifies the overall tendency of a molecule to accept electrons, larger values signify stronger electrophilic character.
- Global Softness (S): another measure inversely related to hardness.
- Additional Electronic Charge (ΔN_max_): represents the maximum amount of electronic charge a molecule can accommodate.

Together, these descriptors provide a detailed picture of how the electronic structure of Cisplatin and its ligands evolves upon complex formation, offering a molecular-level explanation for the observed changes in their chemical reactivity and spectroscopic properties.

It should be emphasized that the computational modelling employed herein is not intended to reproduce intermediate transition states. Instead, the theoretical calculations focus on determining the thermodynamic parameters, specifically the Gibbs free energy of complex formation (ΔG_rs_), between the hydrolysed platinum species and the studied ligands. This approach provides comparative insight into the relative stability of the final adducts, regardless of the specific kinetic pathway. Therefore, the reported ΔG_rs_ values correspond to the overall thermodynamic favourability of complexation, rather than the mechanistic energies of the transition state. The adopted methodology is based on the thermodynamic framework used in previous platinum and ligand studies, such as those by Baik and Lippard [35], and remains consistent with the classical associative mechanism proposed in the literature [32,33,37,38,39,40,85].

### 3.2. Experimental Study

The following reagents were used:

- Pyridine derivatives: Nicotinic acid (99.5% purity) and Nicotinamide (99% purity) from POCH (Avantor Performance Materials Poland S.A., Gliwice, Poland); Isonicotinic acid (100% purity) from Thermo Scientific (Waltham, MA, USA); and Picolinic acid (99% purity) from POL-AURA (Morąg, Poland).
- Nucleosides: Adenosine (99% purity) and Guanosine (98% purity) from TCI (Tokyo, Japan).
- Platinum-based compounds: Cisplatin (98% purity) from Angene (Nanjing, Chine); Carboplatin (98% purity) from TCI (Japan); and Oxaliplatin (98% purity) from POL-AURA (Poland).

Phosphate buffer (pH 7.4) was procured from Witko as as a company representative Chemsolve (Łódź, Poland) and used as the reaction medium. Solution concentrations were measured with a Biosens UV-6000 spectrophotometer, Shanghai, China (1 nm resolution).

Stock solutions of Cisplatin (3.23 × 10^−5^ mol), Carboplatin (3.64 × 10^−5^ mol), and Oxaliplatin (2.47 × 10^−5^ mol) were prepared in phosphate buffer. Each stock solution was then combined with a Pyridine derivative in a 1:2 molar ratio. The mixtures were incubated at 37 °C, and aliquots were collected at predetermined time points: 0, 3, 12, 24, 36 and 168 h. Control samples consisted of individual solutions of the platinum drugs, the Pyridine derivatives, Adenosine, and Guanosine in phosphate buffer.

## 4. Conclusions

The combined computational and spectroscopic studies provide a consistent and multidimensional understanding of the interactions between platinum-based chemotherapeutics (Cisplatin, Carboplatin, and Oxaliplatin) and Pyridine derivatives (Nicotinic acid, Nicotinamide, Isonicotinic acid, and Picolinic acid). The results confirm that these interactions are both thermodynamically favourable and kinetically diverse (described in the manuscript as real-time evolution of UV spectra), revealing a fundamental interplay between structure, reactivity, and stability.

All hydrolysed platinum species (Cis_1, Cis_2, Car_1, Car_2, Oxa_1, Oxa_2) exhibit negative Gibbs free energy changes (ΔG_rs_) upon complexation with all ligands studied, confirming that the formation of Pt–ligand complexes is spontaneous (Mechanism I). The exception are complexes formed with Picolinic acid (Mechanism II). Guanine, consistent with its role in DNA cross-linking, shows the strongest overall affinity among all tested compounds, while Nicotinamide (B3_B) demonstrates the highest binding tendency among the Pyridine derivatives. This behaviour is consistent across both computational approaches (B3LYP and MN15) and in both reaction mechanisms (Mechanism I and II), though quantitative differences arise from methodological variations in electron correlation and solvation treatment and the reaction mechanism under consideration.

A significant structure activity relationship was observed among the pyridine derivatives. The position of the substituent on the pyridine ring strongly affects both thermodynamic affinity and kinetic behaviour (time-dependent complex formation). Picolinic acid (B3_D), characterized by a 2-carboxylic substitution, exhibits the lowest calculated affinity (least negative ΔGr) across nearly all platinum species and methods, yet shows the most rapid and intense spectral evolution in the experimental UV-Vis studies. This kinetic–thermodynamic disparity indicates that the chelate effect associated with the 2-carboxylic group provides a low-energy transition pathway, facilitating rapid coordination to the platinum centre, although the resulting five-membered chelate ring is less stable enthalpically and entropically compared to non-chelated analogues. Conversely, Nicotinamide (B3_B) forms both kinetically accessible and thermodynamically stable complexes. Its low HOMO–LUMO energy gap (ΔE_gap_ ≈ 5.8 eV) indicates electronic “softness,” which enhances interaction with the soft platinum(II) centre through favourable soft–soft acid–base matching.

In addition, by analyzing the ligand exchange reactions exclusively through the thermodynamically relevant Mechanism II, this study provides a realistic assessment of ligand affinity under aqueous conditions. The water substitution ΔG_r_ values confirm that all pyridine derivatives can thermodynamically outcompete coordinated water, although with markedly different efficiencies. Nicotinamide (B3_B) is identified as the most favourable pyridine ligand, consistent with its low HOMO–LUMO energy gap and electronic softness, while Picolinic acid (B3_D), despite its rapid chelation kinetics, forms the least stable complexes due to its higher electronic hardness.

These findings demonstrate that thermodynamic stability is governed primarily by ligand electronic properties, whereas kinetic behaviour is strongly influenced by structural and chelation geometry. Together, these mechanistic insights refine the understanding of Pt(II)–ligand substitution in biologically relevant environments and offer a robust framework for predicting reactivity toward non-canonical nitrogen donors such as pyridine derivatives.

This property correlates directly with its most negative ΔGrs values and strongest UV-Vis absorbance changes, confirming a synergistic relationship between electronic structure and complex stability.

Observations obtained by UV-Vis spectroscopy provide qualitative (rather than quantitative) kinetic information on the time-dependent complex formation. The UV-Vis kinetic analyses further validate the theoretical models derived from DFT calculations. The observed time-dependent absorbance changes confirm a multistep mechanism involving initial drug hydrolysis followed by ligand substitution. Among all ligands, Picolinic acid (B3_D) displayed the fastest chromophore development, while Nicotinamide (B3_B) produced the most stable long-term spectral profile, in line with computational predictions.

Overall, these results establish that:

- Thermodynamic stability (ΔG_rs_) is governed primarily by the ligand’s electronic properties (HOMO–LUMO gap, softness, and electronegativity);
- Reaction kinetics are significantly influenced by structural factors such as chelation geometry and substituent position;
- Soft–soft electronic complementarity between Pt(II) centres and soft nitrogen-donor ligands underlies the high affinity of Nicotinamide and related compounds.

The integration of theoretical and spectroscopic evidence provides a unified framework for understanding ligand substitution in platinum pharmacophores. These insights may support the rational design of new platinum-based agents or prodrugs with tuneable kinetic and thermodynamic profiles, optimized for selective biological interactions and reduced off-target toxicity.

In light of the obtained theoretical and spectroscopic results, which confirm the spontaneous and thermodynamically favourable ability of Pyridine derivatives to form complexes with platinum-based drugs, it is of key importance that the strong interaction with Nicotinamide has also been confirmed in the solid state. The authors confirm that a stable cisplatin–nicotinamide complex has been successfully isolated and fully characterized (using techniques such as NMR and DSC). Although complete structural characterization data will be presented in a separate scientific publication, the isolation of this adduct provides definitive empirical evidence that Pyridine derivatives, and, in particular, Nicotinamide, are not only competitive ligands in solution but can also form stable complexes with well-defined structures with platinum-based chemotherapeutic agents. This discovery completes the research cycle, confirming the hypothesis of a significant structural similarity to nucleobases and paving the way for the rational design of new platinum-based drugs or prodrugs.

## Figures and Tables

**Table 1:** Calculated values of the Gibbs free energy changes (ΔG_rs_) for the complexation reactions between the primary and secondary hydrolysis products of the platinum-based drugs, specifically Cisplatin (Cis_1 and Cis_2), Carboplatin (Car_1 and Car_2), and Oxaliplatin (Oxa_1 and Oxa_2); the tested ligands, presented in the table as Mechanism I; and the calculated values of the Gibbs free energy changes (ΔG_rs_) for the complexation reactions with the water substitution reaction in tetracoordinated Pt complexes with studied structures, presented in the table as Mechanism II. The ligands include Pyridine derivatives, such as Nicotinic acid (B3_A), Nicotinamide (B3_B), Isonicotinic acid (B3_C), and Picolinic acid (B3_D), and Nucleobases, such as Adenine (A) and Guanine (G). The calculations were performed using the B3LYP/6-31G(d,p)/LANL2DZ computational method. The optimized structures are presented in Figures S1–S3 of the Supplementary Materials.

Reaction	Mechanism I	Mechanism II
	ΔG_rs_ [kcal/mol]	
Cis_1 + A → Cis_1 − A	−25.86	−14.44
Cis_1 + G → Cis_1 − G	−26.92	−17.00
Cis_1 + B3_A → Cis_1 − B3_A	−20.30	−2.01
Cis_1 + B3_B → Cis_1 − B3_B	−25.33	−7.04
Cis_1 + B3_C → Cis_1 − B3_C	−25.94	−7.65
Cis_1 + B3_D → Cis_1 − B3_D	−17.55	0.73
Cis_2 + A → Cis_2 − A	−27.15	−18.18
Cis_2 + G → Cis_2 − G	−33.33	−25.85
Cis_2 + B3_A → Cis_2 − B3_A	−27.27	−11.43
Cis_2 + B3_B → Cis_2 − B3_B	−28.56	−12.72
Cis_2 + B3_C → Cis_2 − B3_C	−26.79	−10.94
Cis_2 + B3_D → Cis_2 − B3_D	−22.38	−6.53
Car_1 + A → Car_1 − A	−25.73	−7.4
Car_1 + G → Car_1 − G	−34.12	−17.53
Car_1 + B3_A → Car_1 − B3_A	−26.96	−1.99
Car_1 + B3_B → Car_1 − B3_B	−28.54	−3.58
Car_1 + B3_C → Car_1 − B3_C	−27.16	−2.19
Car_1 + B3_D → Car_1 − B3_D	−19.97	5.00
Car_2 + A → Car_2 − A	−26.46	−15.15
Car_2 + G → Car_2 − G	−30.89	−19.66
Car_2 + B3_A → Car_2 − B3_A	−21.70	−9.51
Car_2 + B3_B → Car_2 − B3_B	−22.34	−10.15
Car_2 + B3_C → Car_2 − B3_C	−21.76	−9.57
Car_2 + B3_D → Car_2 − B3_D	−16.64	−4.27
Oxa_1 + A → Oxa_1 − A	−29.20	−12.01
Oxa_1 + G → Oxa_1 − G	−34.52	−17.99
Oxa_1 + B3_A → Oxa_1 − B3_A	−16.78	−1.46
Oxa_1 + B3_B → Oxa_1 − B3_B	−21.95	−6.63
Oxa_1 + B3_C → Oxa_1 − B3_C	−21.61	−6.29
Oxa_1 + B3_D → Oxa_1 − B3_D	−14.75	0.56
Oxa_2 + A → Oxa_2 − A	−31.98	−13.12
Oxa_2 + G → Oxa_2 − G	−39.97	−23.10
Oxa_2 + B3_A → Oxa_2 − B3_A	−25.34	−8.16
Oxa_2 + B3_B → Oxa_2 − B3_B	−26.43	−9.26
Oxa_2 + B3_C → Oxa_2 − B3_C	−24.73	−7.56
Oxa_2 + B3_D → Oxa_2 − B3_D	−17.97	−0.80

**Table 2:** Calculated values of the Gibbs free energy changes (ΔG_rs_) for the complexation reactions between the primary and secondary hydrolysis products of the platinum-based drugs, specifically Cisplatin (Cis_1 and Cis_2), Carboplatin (Car_1 and Car_2), and Oxaliplatin (Oxa_1 and Oxa_2) and the tested ligands, presented in the table as Mechanism I. Calculated values of the Gibbs free energy changes (ΔG_rs_) for the complexation reactions with the water substitution reaction in tetracoordinated Pt complexes with studied structures presented in the table as Mechanism II. The ligands include Pyridine derivatives, namely, Nicotinic acid (B3_A), Nicotinamide (B3_B), Isonicotinic acid (B3_C), and Picolinic acid (B3_D), and the Nucleobases Adenine (A) and Guanine (G). The calculations were performed using the MN15/def2-TZV computational method. The optimized structures are presented in Figures S1–S3 of the Supplementary Materials.

Reaction	Mechanism I	Mechanism II
	ΔG_rs_ [kcal/mol]	
Cis_1 + A → Cis_1 − A	−43.06	−30.86
Cis_1 + G → Cis_1 − G	−47.66	−35.46
Cis_1 + B3_A → Cis_1 − B3_A	−22.27	−10.07
Cis_1 + B3_B → Cis_1 − B3_B	−27.89	−15.69
Cis_1 + B3_C → Cis_1 − B3_C	−27.22	−15.02
Cis_1 + B3_D → Cis_1 − B3_D	−22.55	−10.35
Cis_2 + A → Cis_2 − A	−27.08	−17.45
Cis_2 + G → Cis_2 − G	−31.18	−21.55
Cis_2 + B3_A → Cis_2 − B3_A	−25.90	−16.27
Cis_2 + B3_B → Cis_2 − B3_B	−26.46	−16.84
Cis_2 + B3_C → Cis_2 − B3_C	−24.94	−15.31
Cis_2 + B3_D → Cis_2 − B3_D	−23.51	−13.89
Car_1 + A → Car_1 − A	−27.08	−17.02
Car_1 + G → Car_1 − G	−34.66	−24.59
Car_1 + B3_A → Car_1 − B3_A	−25.61	−9.27
Car_1 + B3_B → Car_1 − B3_B	−25.68	−9.33
Car_1 + B3_C → Car_1 − B3_C	−25.54	−9.19
Car_1 + B3_D → Car_1 − B3_D	−19.83	−3.49
Car_2 + A → Car_2 − A	−28.16	−20.14
Car_2 + G → Car_2 − G	−29.48	−21.46
Car_2 + B3_A → Car_2 − B3_A	−25.34	−17.32
Car_2 + B3_B → Car_2 − B3_B	−26.00	−17.98
Car_2 + B3_C → Car_2 − B3_C	−26.48	−18.46
Car_2 + B3_D → Car_2 − B3_D	−20.21	−12.19
Oxa_1 + A → Oxa_1 − A	−27.36	−18.26
Oxa_1 + G → Oxa_1 − G	−31.22	−22.12
Oxa_1 + B3_A → Oxa_1 − B3_A	−20.68	−11.57
Oxa_1 + B3_B → Oxa_1 − B3_B	−26.58	−17.48
Oxa_1 + B3_C → Oxa_1 − B3_C	−26.18	−17.08
Oxa_1 + B3_D → Oxa_1 − B3_D	−15.44	−6.34
Oxa_2 + A → Oxa_2 − A	−25.05	−16.85
Oxa_2 + G → Oxa_2 − G	−32.92	−24.72
Oxa_2 + B3_A → Oxa_2 − B3_A	−24.67	−16.47
Oxa_2 + B3_B → Oxa_2 − B3_B	−24.80	−16.59
Oxa_2 + B3_C → Oxa_2 − B3_C	−24.78	−16.58
Oxa_2 + B3_D → Oxa_2 − B3_D	−20.42	−12.22

**Table 3:** General trends for studied molecules (B3LYP/MN15).

Property	Nucleobases (Adenine (A), Guanine (G))	Pyridine Derivatives (B3_A, B3_B, B3_C, B3_D)	Platinum Hydrolysis Products (Cis_1, Car_1, Oxa_2, etc.)
HOMO Energy (E_HOMO_)	Significantly Higher Less Negative, (e.g., G ≈ −6.0 eV)	Lower (More Negative, e.g., B3_A ≈ −7.7 eV)	Generally lower than ligands.
Conclusion	These compounds are superior electron donors (nucleophiles).	Poorer electron donors compared to nucleobases.	
Electrophilicity (ω)	Lower (e.g., G ≈ 13.7 eV).	Higher (e.g., B3_D ≈ 33.4).	Significantly Highest (e.g., Cis_2).
Conclusion	Weaker electrophiles.	Weaker electrophiles compared to Pt-drugs.	Strong electrophiles (electron acceptors).
Energy Gap (ΔE_gap_)	High (5.8–5.9 eV).	High (5.8–6.2 eV).	Significantly Lowest (4.3–4.5 eV).

**Table 4:** The detailed comparison of theoretical and experimental results of UV-Vis study for the studied molecules. The analyzed parameters correspond to the complexation reactions between the primary and secondary hydrolysis products of platinum-based drugs: Cisplatin (Cis_1 and Cis_2), Carboplatin (Car_1 and Car_2), and Oxaliplatin (Oxa_1 and Oxa_2) and the tested ligands. The ligands include Pyridine derivatives: Nicotinic acid (B3_A), Nicotinamide (B3_B), Isonicotinic acid (B3_C), and Picolinic acid (B3_D), as well as nucleobases: Adenine (A) and Guanine (G). All calculations were performed using the B3LYP/6-31G(d,p)/LANL2DZ computational method.

Structure	B3LYP/6-31G(d,p)/LANL2DZ	MN15/def2-TZVP	Experimental Data	Experimental Data from Literature
A (Adenina)	237.2	234.0	218.0; 264.0	220.0; 260.0 [64,65]
G (Guanina)	232.5	227.8	219.00; 260.0	219.0; 249.0; 270.0 [64,65,66]
B3_A Nicotinic acid (3-pyridinecarboxylic acid)	241.0	238.0	236.0; 263.0	215.0; 260.0 [67]
B3_B Nicotinamide (pyridine-3-carboxamide)	252.4	244.6	239.0; 262.0	215.0; 265.0 [68]
B3_C Isonicotinic acid (pyridine-4-carboxylic)	256.1	245.8	237.0; 268.0	215.0; 260.0 [69]
B3_D Picolinic acid (pyridine-2-carboxylic)	239.8	229.0; 266.8	236.0; 268.0	228.0; 245.0; 265.0 [70,71]
Car			219.0; 275.0	220.0; 270.0 [51,72]
Cis			219.0, 276.0	207.0; 275.0 [73]
Oxa			218.0, 274.0	210.0; 260.0 [72]
Car_A			219.0; 262.0	
Car_G			219; 260.0	
Cis_A			218.0; 265.0	
Cis_G			220.0; 265.0	
Oxa_A			220.0; 263.0	
Oxa_G			218.0; 270.0	
Car-B3_A			221.0; 250; 297.0	
Car-B3_B			222.0; 255.0	
Car-B3_C			219.0; 264.0	
Car-B3_D			222.0; 264.0	
Cis-B3_A			221.0; 262.0	
Cis-B3_B			223.0; 262.0	
Cis-B3_C			219.0; 266.0	
Cis-B3_D			220.0; 264.0	
Oxa-B3_A			237.0; 263.0	
Oxa-B3_B			222.0; 261.0	
Oxa-B3_C			236.0; 267.0	
Oxa-B3_D			220.0; 264.0	
Car_1	560.7	523.3		
Car_2	582.9	505.8		
Car_1_A	310.4	289.7		
Car_1_G	305.0	260.2		
Car_2_A	280.8	262.6		
Car_2_G	276.2	266.3		
Car_1-B3_A	292.8	279.2		
Car_1-B3_B	294.4	279.2		
Car_1-B3_C	290.4	284.8		
Car_1-B3_D	294.4	297.7		
Car_2-B3_A	357.4	297.6		
Car_2-B3_B	340.3	299.2		
Car_2-B3_C	376.0	374.0; 291.0		
Car_2-B3_D	367.0	352.0		
Cis_1	489.6	463.7		
Cis_2	555.0	405.0; 520.2		
Cis_1_A	316.6	287.4		
Cis_1_G	319.2	286.8		
Cis_2_A	306.1	289.7		
Cis_2_G	306.1	292.5		
Cis_1-B3_A	309.6	292.4		
Cis_1-B3_B	310.4	283.3		
Cis_1-B3_C	331.2	303.2		
Cis_1-B3_D	334.0	303.7		
Cis_2-B3_A	316.0	284.8		
Cis_2-B3_B	320.8	270.1		
Cis_2-B3_C	301.6	295.3		
Cis_2-B3_D	334.0	303.0		
Oxa 1	457.3	428.1		
Oxa 2	555.0	514.1		
Oxa 1 A	315.8	289.8		
Oxa 1 G	301.1	283.2		
Oxa 2 A	309.6	285.3		
Oxa 2 G	308.0	260.3		
Oxa_1-B3_A	309.6	292.5		
Oxa_1-B3_B	310.4	288.3		
Oxa_1-B3_C	331.2	303.2		
Oxa_1-B3_D	334.0	303.7		
Oxa_2-B3_A	316.0	284.8		
Oxa_2-B3_B	320.8	270.1		
Oxa_2-B3_C	301.6	295.3		
Oxa_2-B3_D	334.0	303.0		

## Data Availability

The original contributions presented in this study are included in the article/Supplementary Material. Further inquiries can be directed to the corresponding author.

## Supplementary Materials

The following supporting information can be downloaded at: https://www.mdpi.com/article/10.3390/ijms262411875/s1.
