# Supplementary material for: Platinum Meets Pyridine: Affinity Studies of Pyridinecarboxylic Acids and Nicotinamide for Platinum—Based Drugs
Source: Int J Mol Sci. 2025 Dec 9;26(24):11875. doi: 10.3390/ijms262411875 (PMC12732872; doi:10.3390/ijms262411875)
Supplement: Supplementary file 1 [file ijms-26-11875-s001.zip › ijms-3963529-supplementary.pdf]

Platinum Meets Pyridine: Affinity Studies of Pyridinecarboxylic Acids and Nicotinamide for Platinum-Based Drugs

Beata Szefler[a*], Kamil Szupryczyński[b], Przemysław Czeleń[a]

[a]*Department of Physical Chemistry, Faculty of Pharmacy, Collegium Medicum, Nicolaus Copernicus University, Kurpińskiego 5, 85-096, Bydgoszcz, Poland.*
**Correspondence: beatas@cm.umk.pl*

[b]*Doctoral School of Medical and Health Sciences, Faculty of Pharmacy, Collegium Medicum, Nicolaus Copernicus University, Jagiellońska 13, 85-067 Bydgoszcz, Poland*

Table of Contents:

Figure S1. *Structures of Carboplatin complexes (the primary and secondary hydrolysis products of platinum-based drug) with nucleobases (Adenine (A), Guanine (G)) and pyridine derivatives: Nicotinic acid (3-pyridinecarboxylic acid, B3_A), Nicotinamide (pyridine-3-carboxamide, B3_B), Isonicotinic acid (pyridine-4-carboxylic, B3_C), and Picolinic acid (pyridine-2-carboxylic, B3_D) calculated at the B3LYP/6-31G(d,p)/LANL2DZ and MN15/def2-TZVP levels of theory, using PCM with water as solvent.*

Figure S2. *Structures of Cisplatin complexes (the primary and secondary hydrolysis products of platinum-based drug) with nucleobases (Adenine (A), Guanine (G)) and pyridine derivatives: Nicotinic acid (3-pyridinecarboxylic acid, B3_A), Nicotinamide (pyridine-3-carboxamide, B3_B), Isonicotinic acid (pyridine-4-carboxylic, B3_C), and Picolinic acid (pyridine-2-carboxylic, B3_D) calculated at the B3LYP/6-31G(d,p)/LANL2DZ and MN15/def2-TZVP levels of theory, using PCM with water as solvent.*

Figure S3. *Structures of Oxaliplatin (the primary and secondary hydrolysis products of platinum-based drug) with nucleobases (Adenine (A), Guanine (G)) and pyridine derivatives: Nicotinic acid (3-pyridinecarboxylic acid, B3_A), Nicotinamide (pyridine-3-carboxamide, B3_B), Isonicotinic acid (pyridine-4-carboxylic, B3_C), and Picolinic acid (pyridine-2-carboxylic, B3_D) calculated at the B3LYP/6-31G(d,p)/LANL2DZ and MN15/def2-TZVP levels of theory, using PCM with water as solvent.*

Figure S4. *UV–vis spectra of Carboplatin recorded after 1, 3, 12, 24, 36, and 168 hours of incubation at 37 °C.*

Figure S5. *UV–Vis spectra of a mixed solution of Adenosine and Carboplatin recorded after 1, 3, 12, 24, 36, and 168 hours of incubation at 37 °C.*

Figure S6. *UV–Vis spectra of a mixed solution of Guanosine and Carboplatin recorded after 1, 3, 12, 24, 36, and 168 hours of incubation at 37 °C.*

Figure S7. *UV–Vis spectra of a mixed solution of Nicotinic acid (3-pyridinecarboxylic acid, B3_A) and Carboplatin recorded after 1, 3, 12, 24, 36, and 168 hours of incubation at 37 °C.*

Figure S8. *UV–Vis spectra of a mixed solution of Nicotinamide (pyridine-3-carboxamide, B3_B) and Carboplatin recorded after 1, 3, 12, 24, 36, and 168 hours of incubation at 37 °C.*

Figure S9. *UV–Vis spectra of a mixed solution of Isonicotinic acid (pyridine-4-carboxylic, B3_C) and Carboplatin recorded after 1, 3, 12, 24, 36, and 168 hours of incubation at 37 °C.*

Figure S10*. UV–Vis spectra of a mixed solution of Picolinic acid (pyridine-2-carboxylic, B3_D) and Carboplatin recorded after 1, 3, 12, 24, 36, and 168 hours of incubation at 37 °C.*

Figure S11*. UV–vis spectra of Cisplatin recorded after 1, 3, 12, 24, 36, and 168 hours of incubation at 37 °C.*

Figure S12. *UV–Vis spectra of a mixed solution of Adenosine and Cisplatin recorded after 1, 3, 12, 24, 36, and 168 hours of incubation at 37 °C.*

Figure S13. *UV–Vis spectra of a mixed solution of Guanosine and Cisplatin recorded after 1, 3, 12, 24, 36, and 168 hours of incubation at 37 °C.*

Figure S14. *UV–Vis spectra of a mixed solution of Nicotinic acid (3-pyridinecarboxylic acid, B3_A) and Cisplatin recorded after 1, 3, 12, 24, 36, and 168 hours of incubation at 37 °C.*

Figure S15. *UV–Vis spectra of a mixed solution of Nicotinamide (pyridine-3-carboxamide, B3_B) and Cisplatin recorded after 1, 3, 12, 24, 36, and 168 hours of incubation at 37 °C.*

Figure S16. *UV–Vis spectra of a mixed solution of Isonicotinic acid (pyridine-4-carboxylic, B3_C) and Cisplatin recorded after 1, 3, 12, 24, 36, and 168 hours of incubation at 37 °C.*

Figure S17*. UV–Vis spectra of a mixed solution of Picolinic acid (pyridine-2-carboxylic, B3_D) and Cisplatin recorded after 1, 3, 12, 24, 36, and 168 hours of incubation at 37 °C.*

Figure S18*. UV–vis spectra of Oxaliplatin recorded after 1, 3, 12, 24, 36, and 168 hours of incubation at 37 °C.*

Figure S19. *UV–Vis spectra of a mixed solution of Adenosine and Oxaliplatin recorded after 1, 3, 12, 24, 36, and 168 hours of incubation at 37 °C.*

Figure S20. *UV–Vis spectra of a mixed solution of Guanosine and Oxaliplatin recorded after 1, 3, 12, 24, 36, and 168 hours of incubation at 37 °C.*

Figure S21. *UV–Vis spectra of a mixed solution of Nicotinic acid (3-pyridinecarboxylic acid, B3_A) and Oxaliplatin recorded after 1, 3, 12, 24, 36, and 168 hours of incubation at 37 °C.*

Figure S22. *UV–Vis spectra of a mixed solution of Nicotinamide (pyridine-3-carboxamide, B3_B) and Oxaliplatin recorded after 1, 3, 12, 24, 36, and 168 hours of incubation at 37 °C.*

Figure S23. *UV–Vis spectra of a mixed solution of Isonicotinic acid (pyridine-4-carboxylic, B3_C) and Oxaliplatin recorded after 1, 3, 12, 24, 36, and 168 hours of incubation at 37 °C.*

Figure S24*. UV–Vis spectra of a mixed solution of Picolinic acid (pyridine-2-carboxylic, B3_D) and Oxaliplatin recorded after 1, 3, 12, 24, 36, and 168 hours of incubation at 37 °C.*

Figure S25. *Calculated UV–Vis absorption spectra of selected nucleobases, Adenine (A) and Guanine (G) and their complexes with the first and second hydrolysis products of Cisplatin (Cis_1 and Cis_2), Carboplatin (Car_1 and Car_2), and Oxaliplatin (Oxa_1 and Oxa_2). The geometries were optimized at the B3LYP/6-31G(d,p)/LANL2DZ and MN15/def2-TZV levels of theory using the PCM model with water as the solvent. All spectroscopic calculations were performed employing the PBE0 functional.*

Figure S26. *Calculated UV–Vis absorption spectra of pyridine derivatives: Nicotinic acid (B3_A), Nicotinamide (B3_B), Isonicotinic acid (B3_C), and Picolinic acid (B3_D), selected nucleobases, Adenine (A) and Guanine (G) and the first and second hydrolysis products of Cisplatin (Cis_1 and Cis_2), Carboplatin (Car_1 and Car_2) and Oxaliplatin (Oxa_1 and Oxa_2). The geometries were optimized at the B3LYP/6-31G(d,p)/LANL2DZ and MN15/def2-TZV levels of theory using the PCM model with water as the solvent. All spectroscopic calculations were performed employing the PBE0 functional.*

Figure S27. *Calculated UV–Vis absorption spectra of the first and second product of hydrolysis of Carboplatin (Car_1 and Car_2) complexes with pyridine derivatives: Nicotinic acid (B3_A), Nicotinamide (B3_B), Isonicotinic acid (B3_C), and Picolinic acid (B3_D). The geometries were optimized at the B3LYP/6-31G(d,p)/LANL2DZ and MN15/def2-TZV levels of theory using the PCM model with water as the solvent. All spectroscopic calculations were performed employing the PBE0 functional.*

Figure S28. *Calculated UV–Vis absorption spectra of the first and second product of hydrolysis of Cisplatin (Cis_1 and Cis_2) complexes with pyridine derivatives: Nicotinic acid (B3_A), Nicotinamide (B3_B),*

Isonicotinic acid (B3_C), and Picolinic acid (B3_D). The geometries were optimized at the B3LYP/6-31G(d,p)/LANL2DZ and MN15/def2-TZV levels of theory using the PCM model with water as the solvent. All spectroscopic calculations were performed employing the PBE0 functional.

Figure S29. *Calculated UV–Vis absorption spectra of the first and second product of hydrolysis of Oxaliplatin (Oxa_1 and Oxa_2) complexes with pyridine derivatives: Nicotinic acid (B3_A), Nicotinamide (B3_B), Isonicotinic acid (B3_C), and Picolinic acid (B3_D). The geometries were optimized at the B3LYP/6-31G(d,p)/LANL2DZ and MN15/def2-TZV levels of theory using the PCM model with water as the solvent. All spectroscopic calculations were performed employing the PBE0 functional.*

Figure S30. *HOMO LUMO plots of nucleobases (Adenine (A), Guanine (G)) and pyridine derivatives: Nicotinic acid (B3_A), Nicotinamide (B3_B), Isonicotinic acid (B3_C), and Picolinic acid (B3_D), and their complexes with the first and second products of hydrolysis of Carboplatin (Car_1, Car_2), Cisplatin (Cis_1, Cis_2) and Oxaliplatin (Oxa_1, Oxa_2), (isovalue = 0.02 a.u., density = 0.0004 e/Å³).*

Table S1. *HOMO–LUMO energies (in eV) and parameters describing the chemical behaviour of the studied molecules, including the energy gap (ΔE_{gap}), absolute electronegativity (χ), chemical potential (μ), absolute hardness (η), absolute softness (σ), global electrophilicity index (ω), global softness (S), and the maximum additional electronic charge (ΔNmax), calculated at the B3LYP/6-31G(d,p)/LANL2DZ level of theory. The calculated parameters correspond to the complexation reactions between the primary and secondary hydrolysis products of platinum-based drugs: Cisplatin (Cis_1 and Cis_2), Carboplatin (Car_1 and Car_2), and Oxaliplatin (Oxa_1 and Oxa_2) and the tested ligands. The ligands include pyridine derivatives: Nicotinic acid (B3_A), Nicotinamide (B3_B), Isonicotinic acid (B3_C), and Picolinic acid (B3_D), as well as nucleobases: Adenine (A) and Guanine (G). All calculations were performed using the B3LYP/6-31G(d,p)/LANL2DZ computational method.*

Table S2. *HOMO–LUMO energies (in eV) and parameters describing the chemical behaviour of the studied molecules, including the energy gap (ΔE_{gap}), absolute electronegativity (χ), chemical potential (μ), absolute hardness (η), absolute softness (σ), global electrophilicity index (ω), global softness (S), and the maximum additional electronic charge (ΔNmax), calculated at the B3LYP/6-31G(d,p)/LANL2DZ level of theory. The calculated parameters correspond to the complexation reactions between the primary and secondary hydrolysis products of platinum-based drugs: Cisplatin (Cis_1 and Cis_2), Carboplatin (Car_1 and Car_2), and Oxaliplatin (Oxa_1 and Oxa_2) and the tested ligands. The ligands include pyridine derivatives: Nicotinic acid (B3_A), Nicotinamide (B3_B), Isonicotinic acid (B3_C), and Picolinic acid (B3_D), as well as nucleobases: Adenine (A) and Guanine (G). All calculations were performed using the MN15/def2-TZV computational method.*

		Products of complexation	
		Car_1	Car_2
Substrate of complexation	Adenine (A)	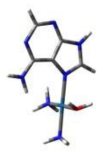	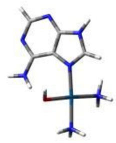
		Car_1-A	Car_2-A
	Guanine (G)	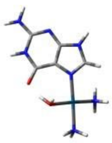	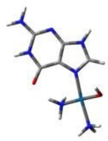
		Car_1-G	Car_2-G
	Nicotinic acid (B3_A)	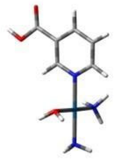	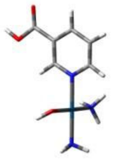
		Car_1-B3_A	Car_2-B3_A
	Nicotinamide (B3_B)	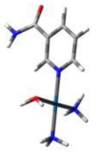	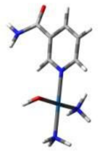
		Car_1- B3_B	Car_2-B3_B
	Isonicotinic acid (B3_C)	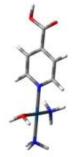	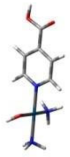
		Car_1-B3_C	Car_2-B3_C
	Picolinic acid (B3_D)	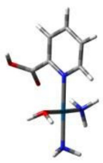	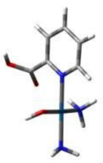
		Car_1-B3_D	Car_2-B3_D

Figure S1. *Structures of Carboplatin complexes (the primary and secondary hydrolysis products of platinum-based drug) with nucleobases (Adenine (A), Guanine (G)) and pyridine derivatives: Nicotinic acid (3-pyridinecarboxylic acid, B3_A), Nicotinamide (pyridine-3-carboxamide, B3_B), Isonicotinic acid (pyridine-4-carboxylic, B3_C), and Picolinic acid (pyridine-2-carboxylic, B3_D) calculated at the B3LYP/6-31G(d,p)/LANL2DZ and MN15/def2-TZVP levels of theory, using PCM with water as solvent.*

		Products of complexation	
		Cis_1	Cis_2
Substrate of complexation	Adenine (A)	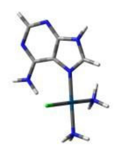	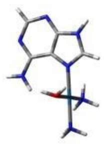
		Cis_1-A	Cis_2-A
	Guanine (G)	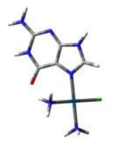	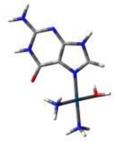
		Cis_1-G	Cis_2-G
	Nicotinic acid (B3_A)	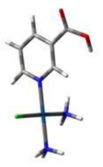	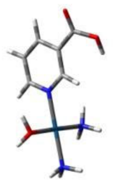
		Cis_1-B3_A	Cis_2-B3_A
	Nicotinamide (B3_B)	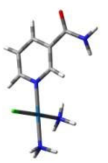	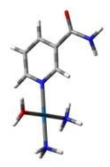
		Cis_1-B3_B	Cis_2-B3_B
	Isonicotinic acid (B3_C)	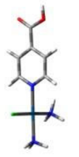	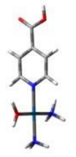
		Cis_1-B3_C	Cis_2-B3_C
	Picolinic acid (B3_D)	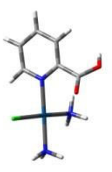	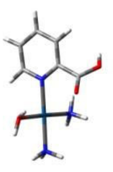
		Cis_1-B3_D	Cis_2-B3_D

Figure S2. *Structures of Cisplatin complexes (the primary and secondary hydrolysis products of platinum-based drug) with nucleobases (Adenine (A), Guanine (G)) and pyridine derivatives: Nicotinic acid (3-pyridinecarboxylic acid, B3_A), Nicotinamide (pyridine-3-carboxamide, B3_B), Isonicotinic acid (pyridine-4-carboxylic, B3_C), and Picolinic acid (pyridine-2-carboxylic, B3_D) calculated at the B3LYP/6-31G(d,p)/LANL2DZ and MN15/def2-TZVP levels of theory, using PCM with water as solvent.*

		Products of complexation	
		Oxa_1	Oxa_2
Substrateofcomplexation	Adenine (A)	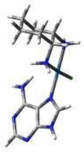 Oxa_1-A	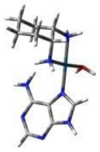 Oxa_2-A
	Guanine (G)	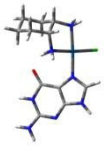 Oxa_1-G	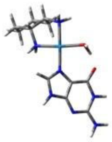 Oxa_2-G
	Nicotinic acid (B3_A)	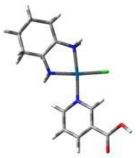 Oxa_1-B3_A	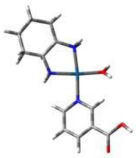 Oxa_2-B3_A
	Nicotinamide (B3_B)	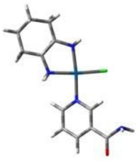 Oxa_1-B3_B	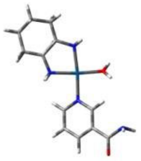 Oxa_2-B3_B
	Isonicotinic acid (B3_C)	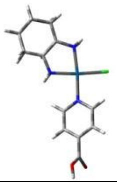 Oxa_1-B3_C	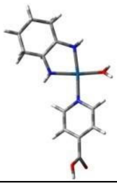 Oxa_2-B3_C
	Picolinic acid (B3_D)	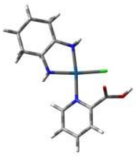 Oxa_1-B3_D	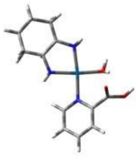 Oxa_2-B3_D

Figure S3. *Structures of Oxaliplatin (the primary and secondary hydrolysis products of platinum-based drug) with nucleobases (Adenine (A), Guanine (G)) and pyridine derivatives: Nicotinic acid (3-pyridinecarboxylic acid, B3_A), Nicotinamide (pyridine-3-carboxamide, B3_B), Isonicotinic acid (pyridine-4-carboxylic, B3_C), and Picolinic acid (pyridine-2-carboxylic, B3_D) calculated at the B3LYP/6-31G(d,p)/LANL2DZ and MN15/def2-TZVP levels of theory, using PCM with water as solvent.*

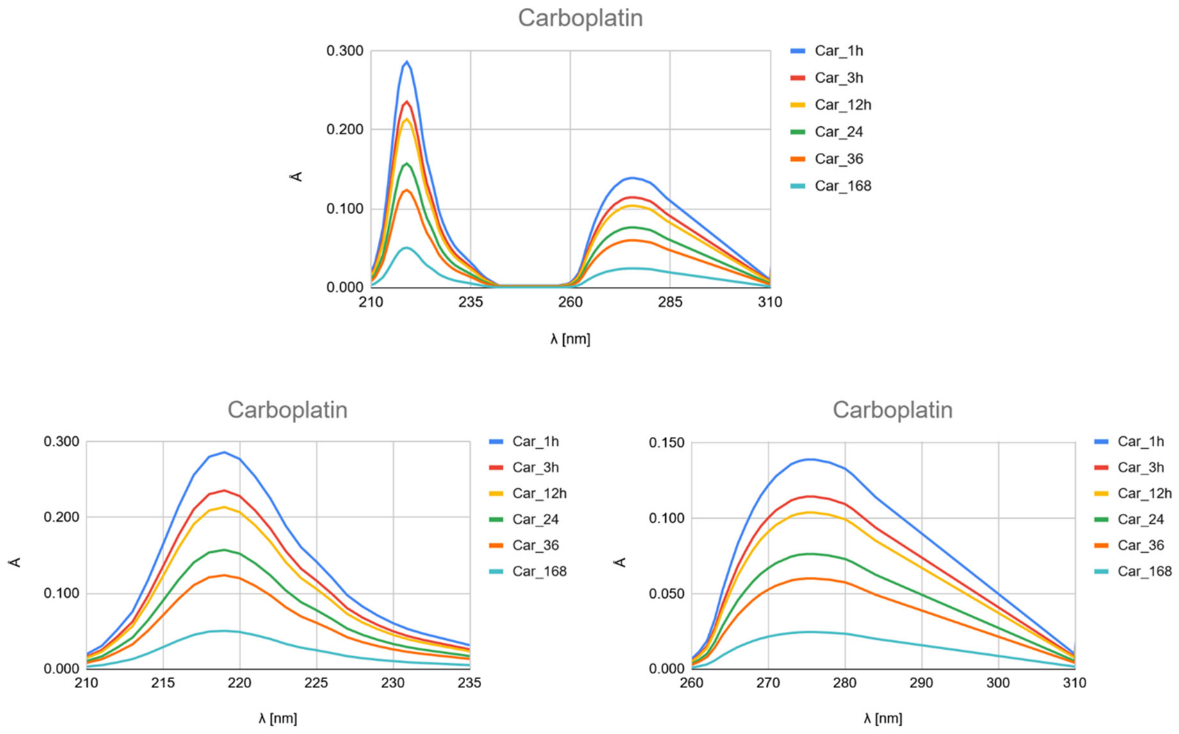

Figure S4. *UV–vis spectra of Carboplatin recorded after 1, 3, 12, 24, 36, and 168 hours of incubation at 37 °C.*

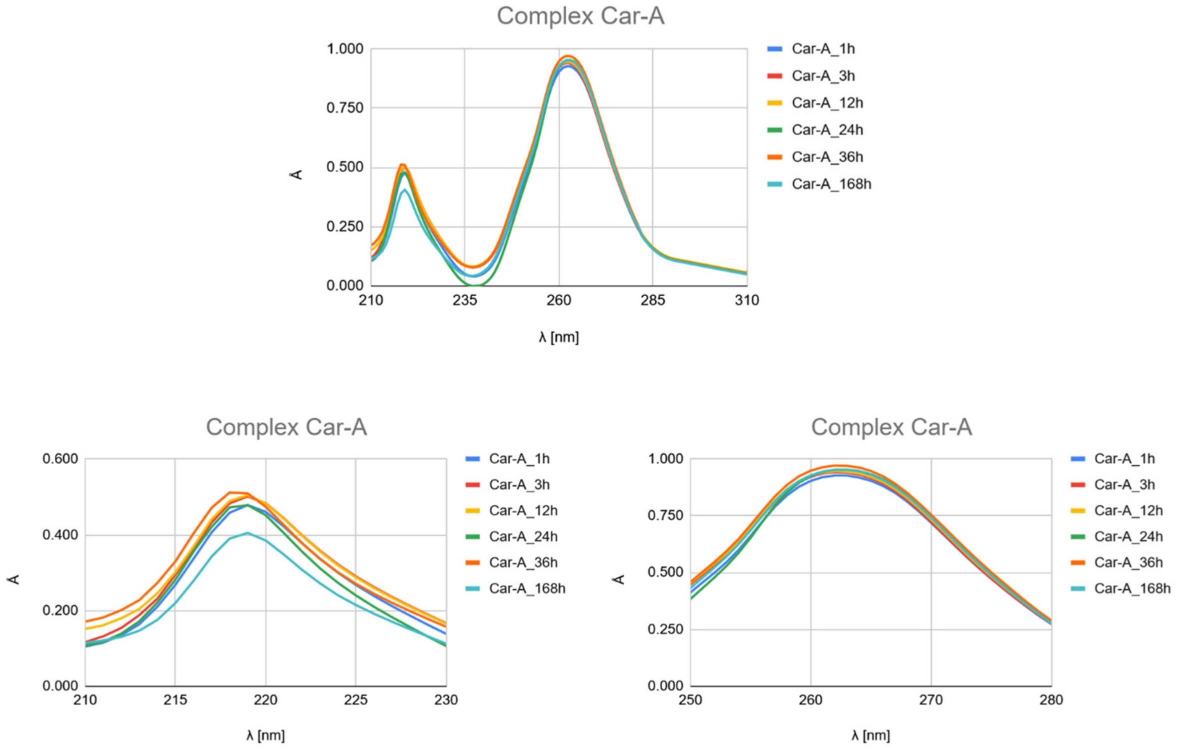

Figure S5. *UV–Vis spectra of a mixed solution of Adenosine and Carboplatin recorded after 1, 3, 12, 24, 36, and 168 hours of incubation at 37 °C.*

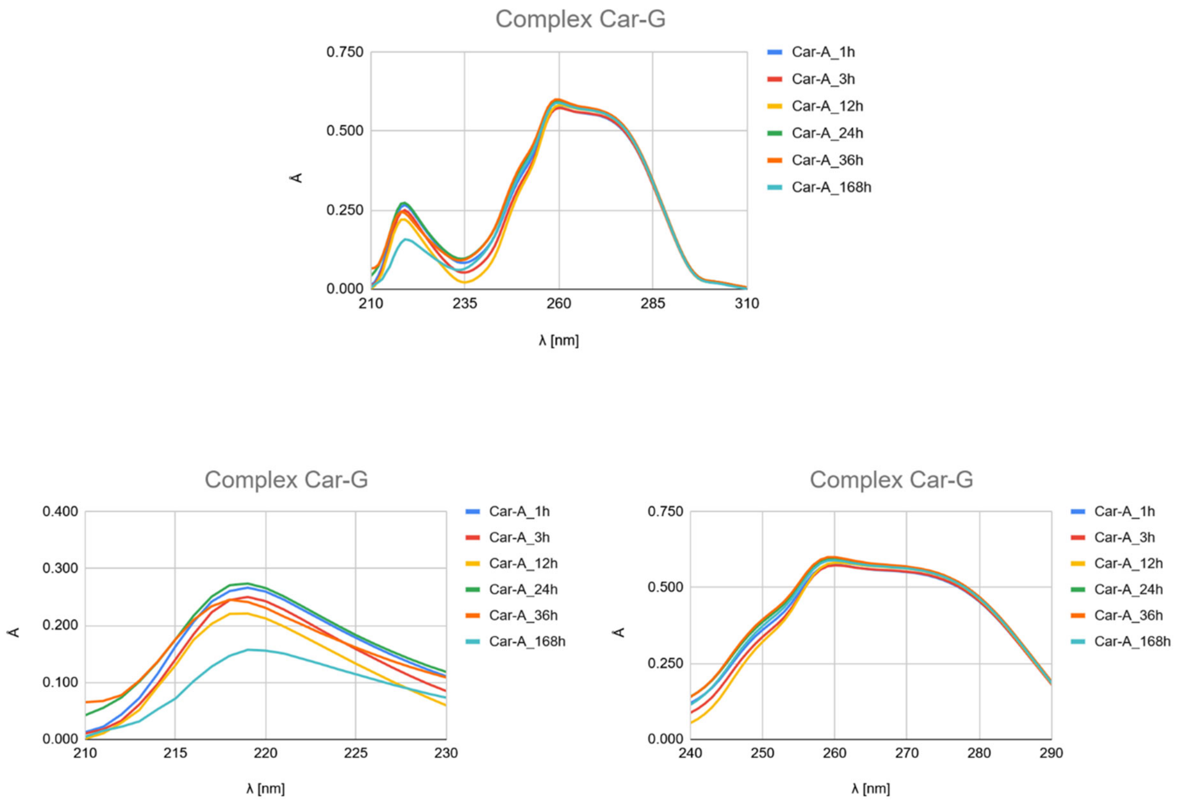

Figure S6. *UV–Vis spectra of a mixed solution of Guanosine and Carboplatin recorded after 1, 3, 12, 24, 36, and 168 hours of incubation at 37 °C.*

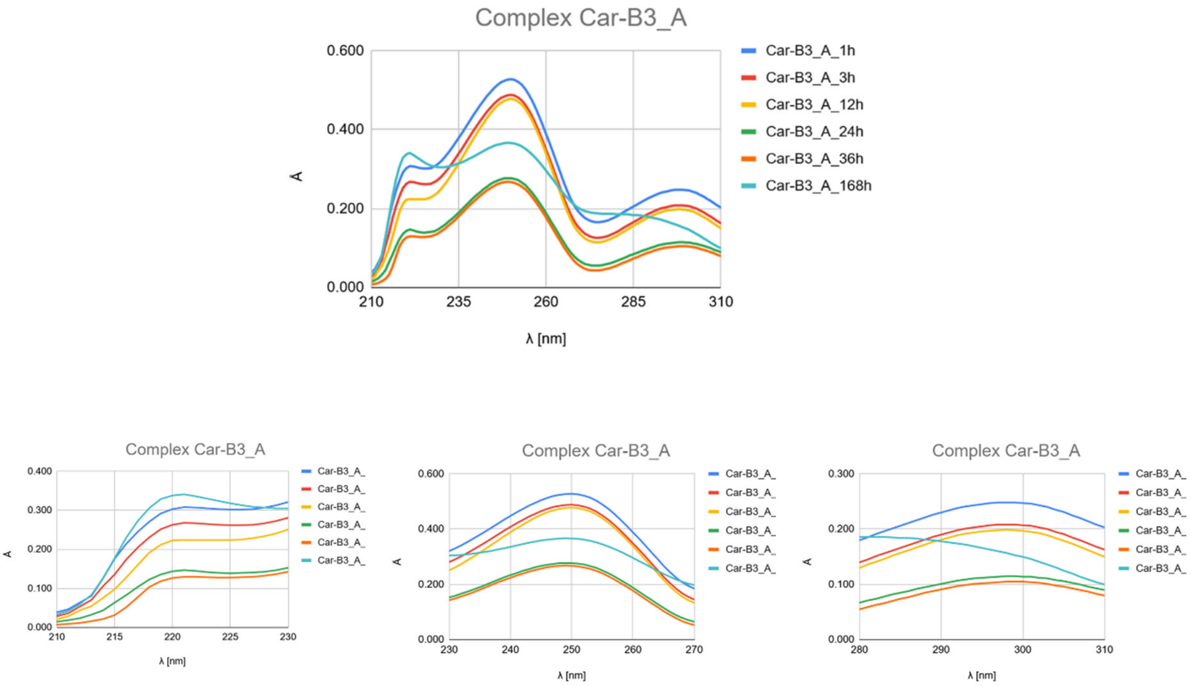

Figure S7. *UV–Vis spectra of a mixed solution of Nicotinic acid (3-pyridinecarboxylic acid, B3_A) and Carboplatin recorded after 1, 3, 12, 24, 36, and 168 hours of incubation at 37 °C.*

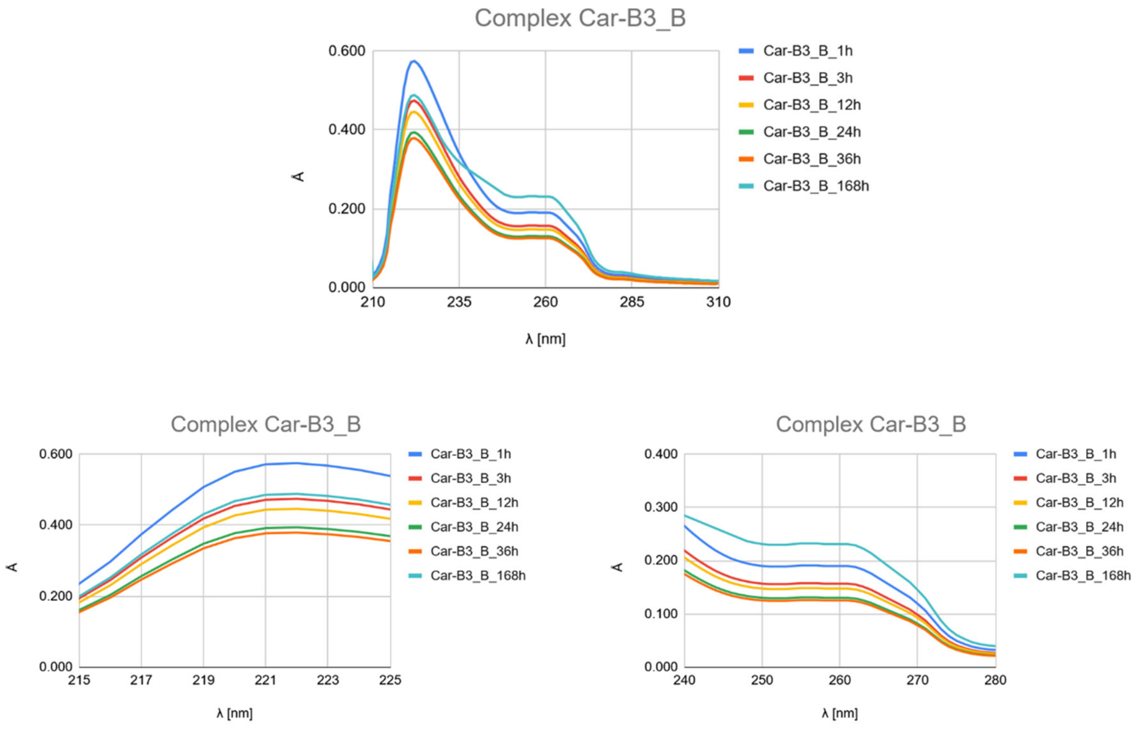

Figure S8. *UV–Vis spectra of a mixed solution of Nicotinamide (pyridine-3-carboxamide, B3_B) and Carboplatin recorded after 1, 3, 12, 24, 36, and 168 hours of incubation at 37 °C.*

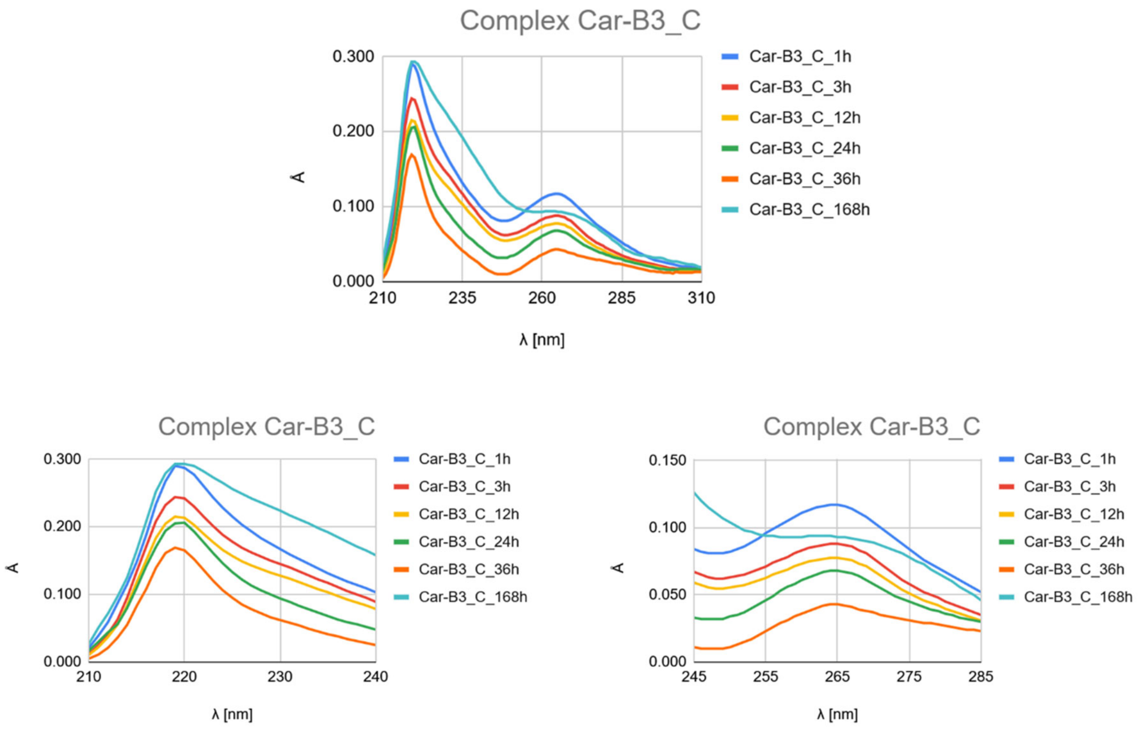

Figure S9. *UV–Vis spectra of a mixed solution of Isonicotinic acid (pyridine-4-carboxylic, B3_C) and Carboplatin recorded after 1, 3, 12, 24, 36, and 168 hours of incubation at 37 °C.*

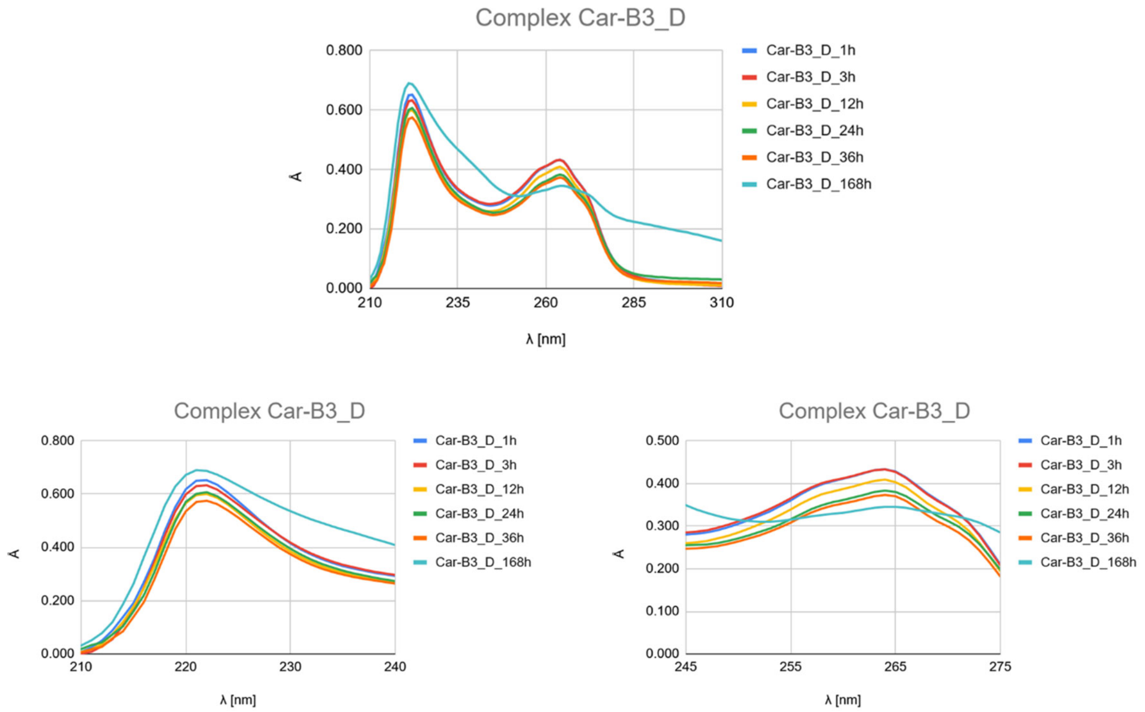

Figure S10*. UV–Vis spectra of a mixed solution of Picolinic acid (pyridine-2-carboxylic, B3_D) and Carboplatin recorded after 1, 3, 12, 24, 36, and 168 hours of incubation at 37 °C.*

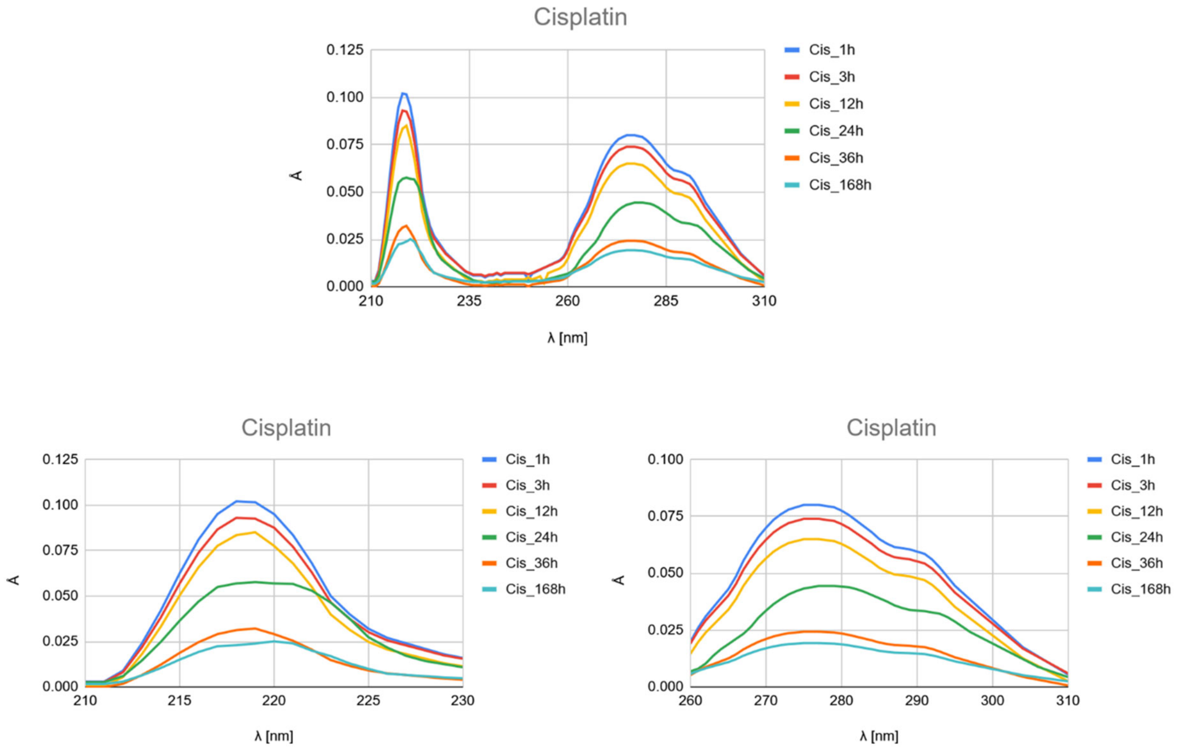

Figure S11. *UV–vis spectra of Cisplatin recorded after 1, 3, 12, 24, 36, and 168 hours of incubation at 37 °C.*

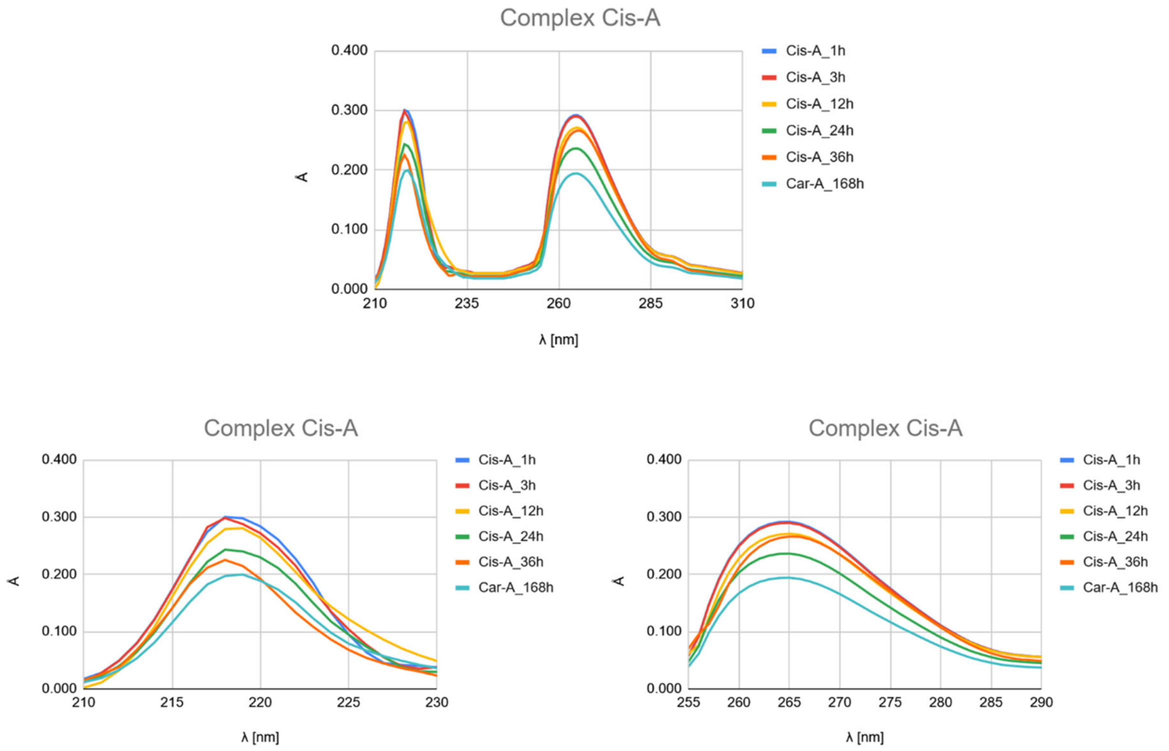

Figure S12. *UV–Vis spectra of a mixed solution of Adenosine and Cisplatin recorded after 1, 3, 12, 24, 36, and 168 hours of incubation at 37 °C.*

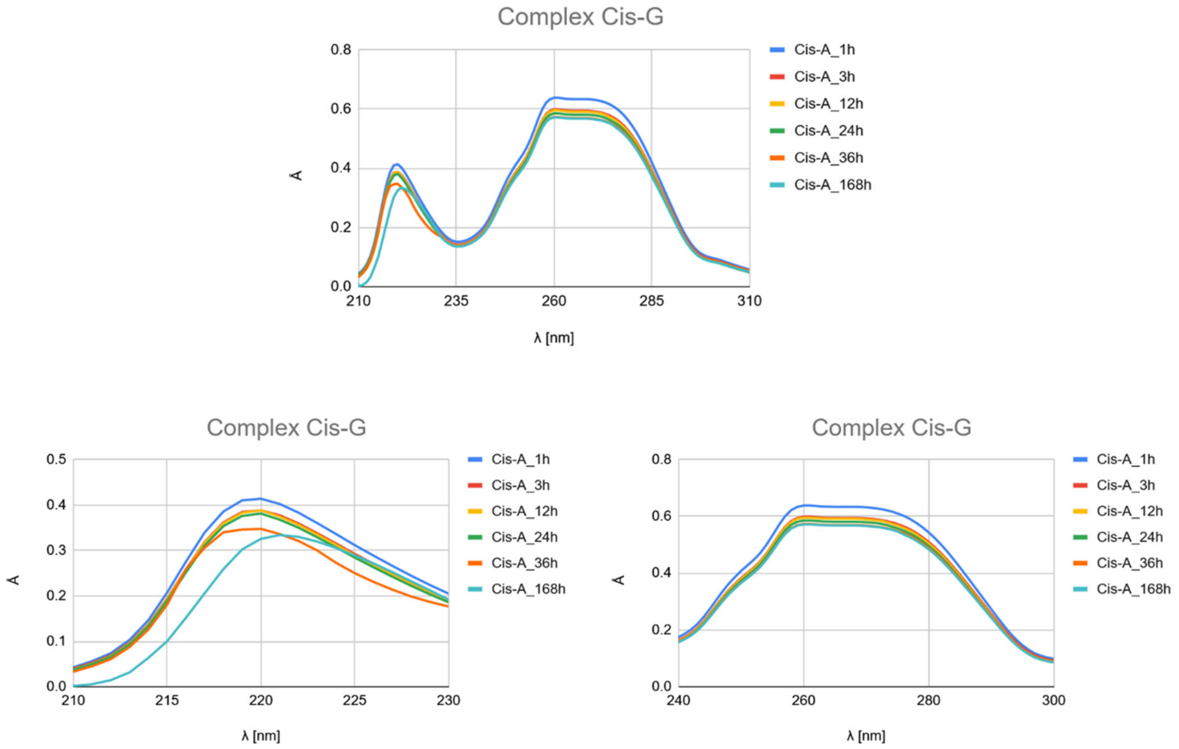

Figure S13. *UV–Vis spectra of a mixed solution of Guanosine and Cisplatin recorded after 1, 3, 12, 24, 36, and 168 hours of incubation at 37 °C.*

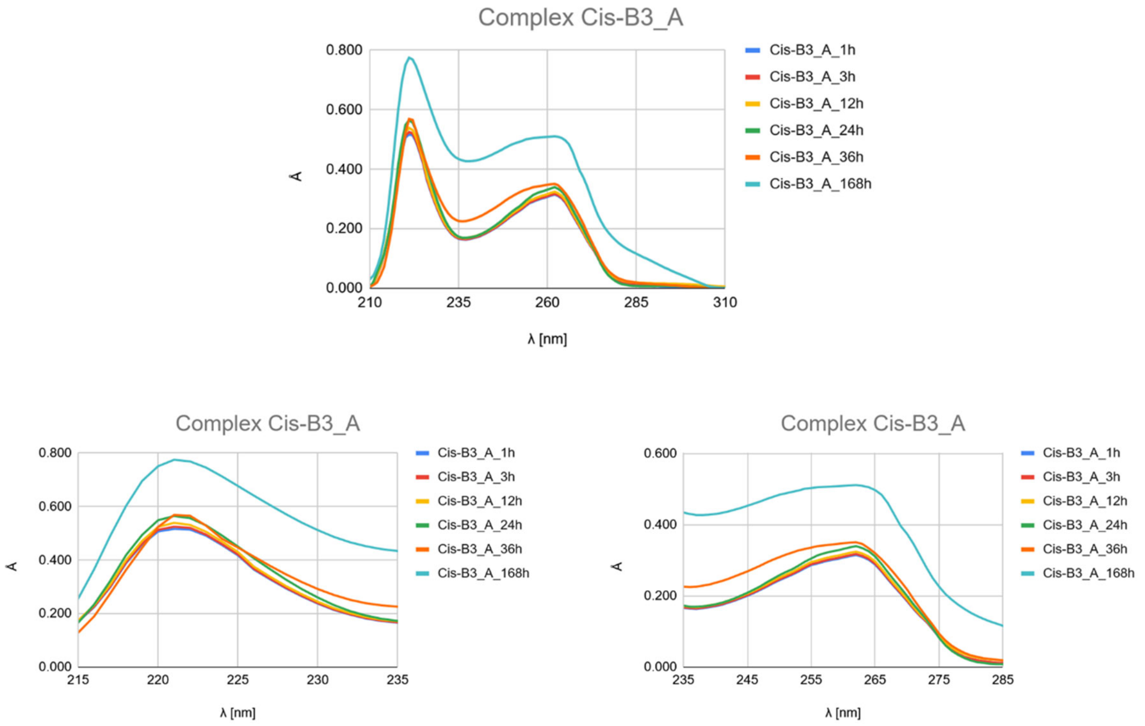

Figure S14. *UV–Vis spectra of a mixed solution of Nicotinic acid (3-pyridinecarboxylic acid, B3_A) and Cisplatin recorded after 1, 3, 12, 24, 36, and 168 hours of incubation at 37 °C.*

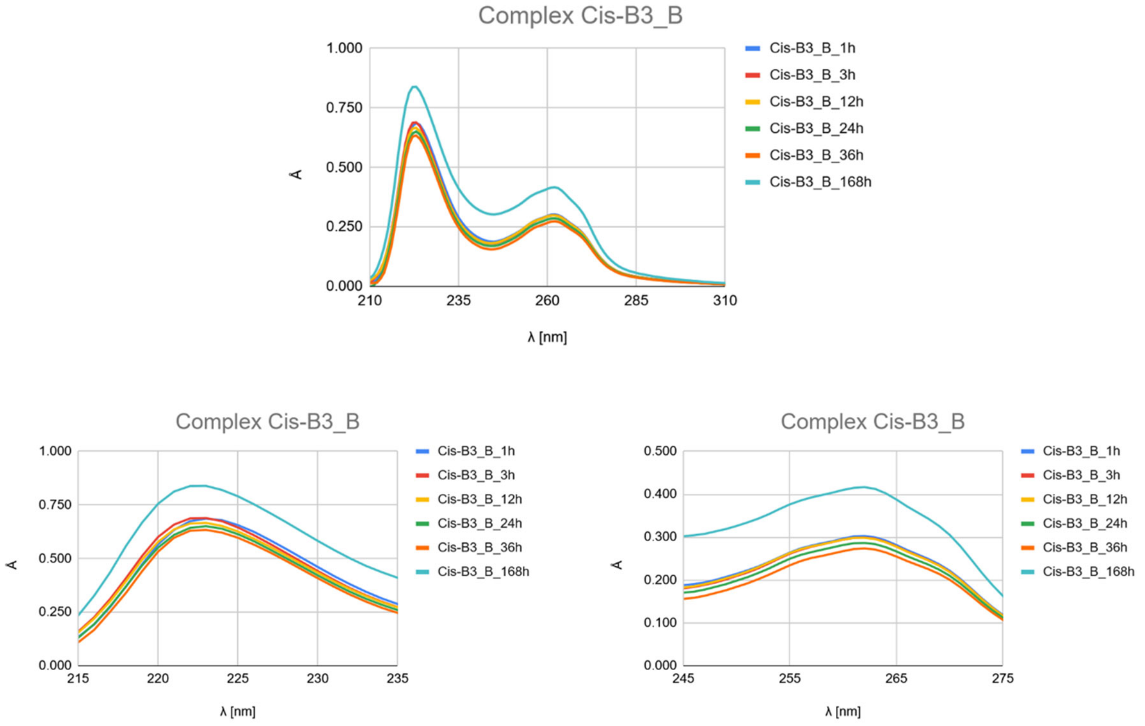

Figure S15. *UV–Vis spectra of a mixed solution of Nicotinamide (pyridine-3-carboxamide, B3_B) and Cisplatin recorded after 1, 3, 12, 24, 36, and 168 hours of incubation at 37 °C.*

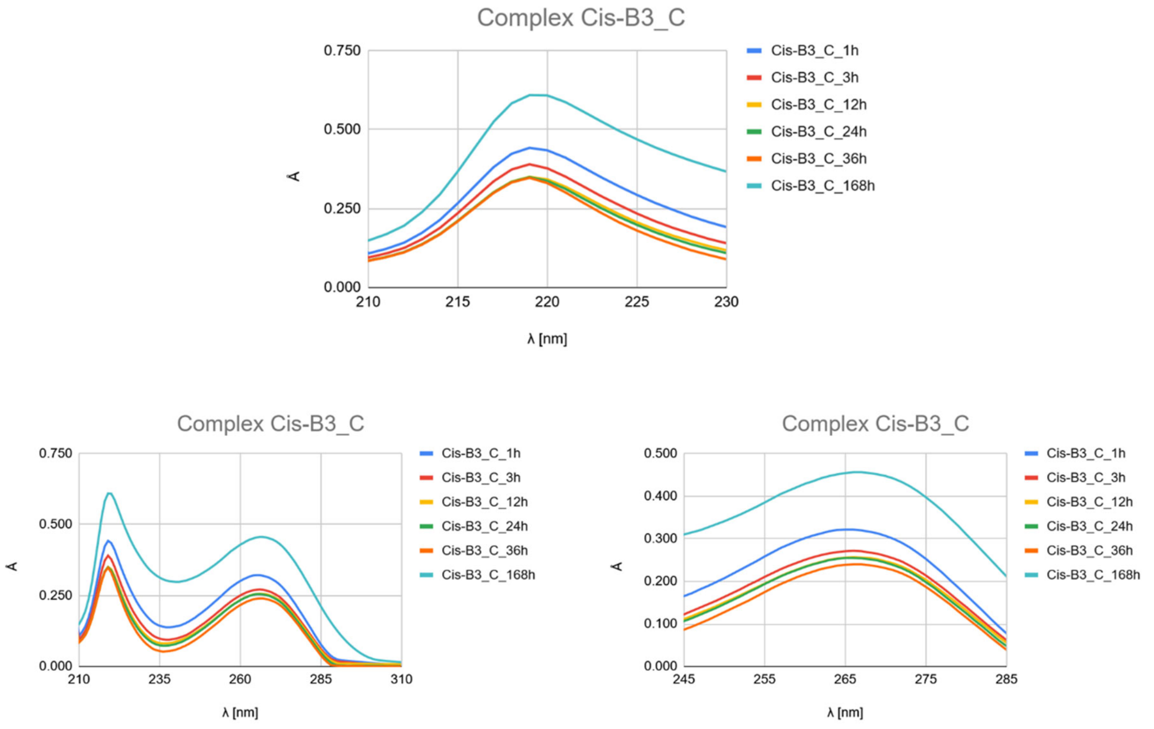

Figure S16. *UV–Vis spectra of a mixed solution of Isonicotinic acid (pyridine-4-carboxylic, B3_C) and Cisplatin recorded after 1, 3, 12, 24, 36, and 168 hours of incubation at 37 °C.*

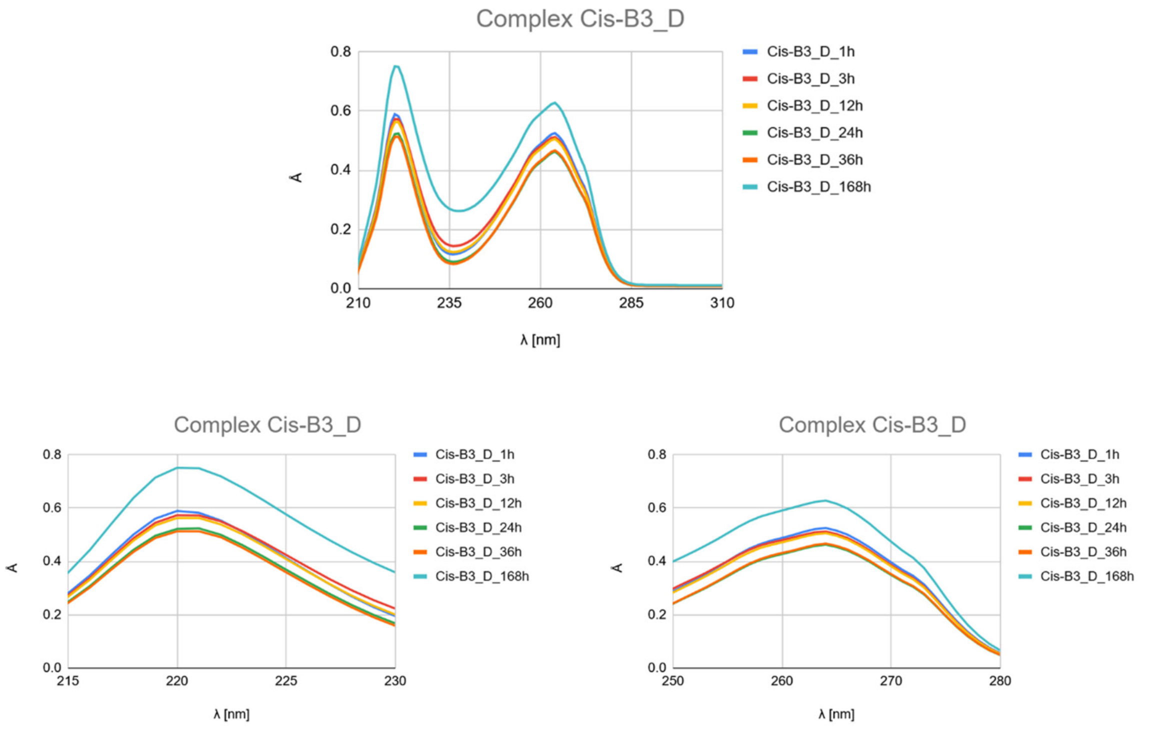

Figure S17*. UV–Vis spectra of a mixed solution of Picolinic acid (pyridine-2-carboxylic, B3_D) and Cisplatin recorded after 1, 3, 12, 24, 36, and 168 hours of incubation at 37 °C.*

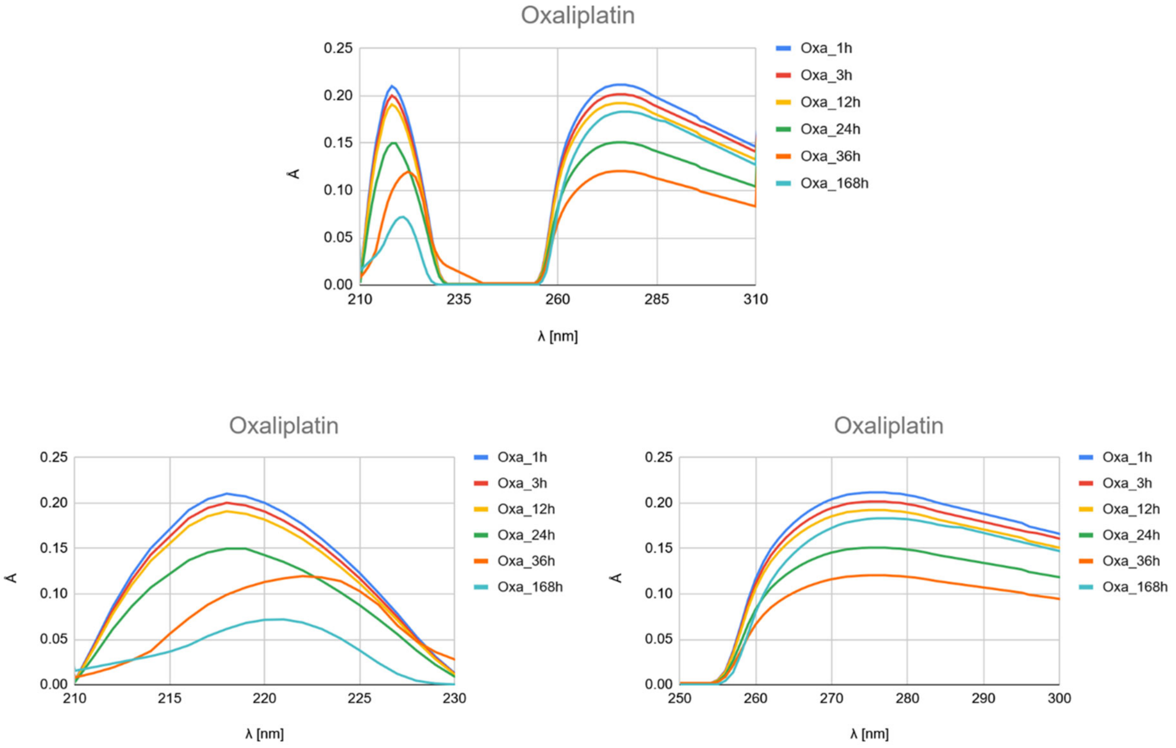

Figure S18. *UV–vis spectra of Oxaliplatin recorded after 1, 3, 12, 24, 36, and 168 hours of incubation at 37 °C.*

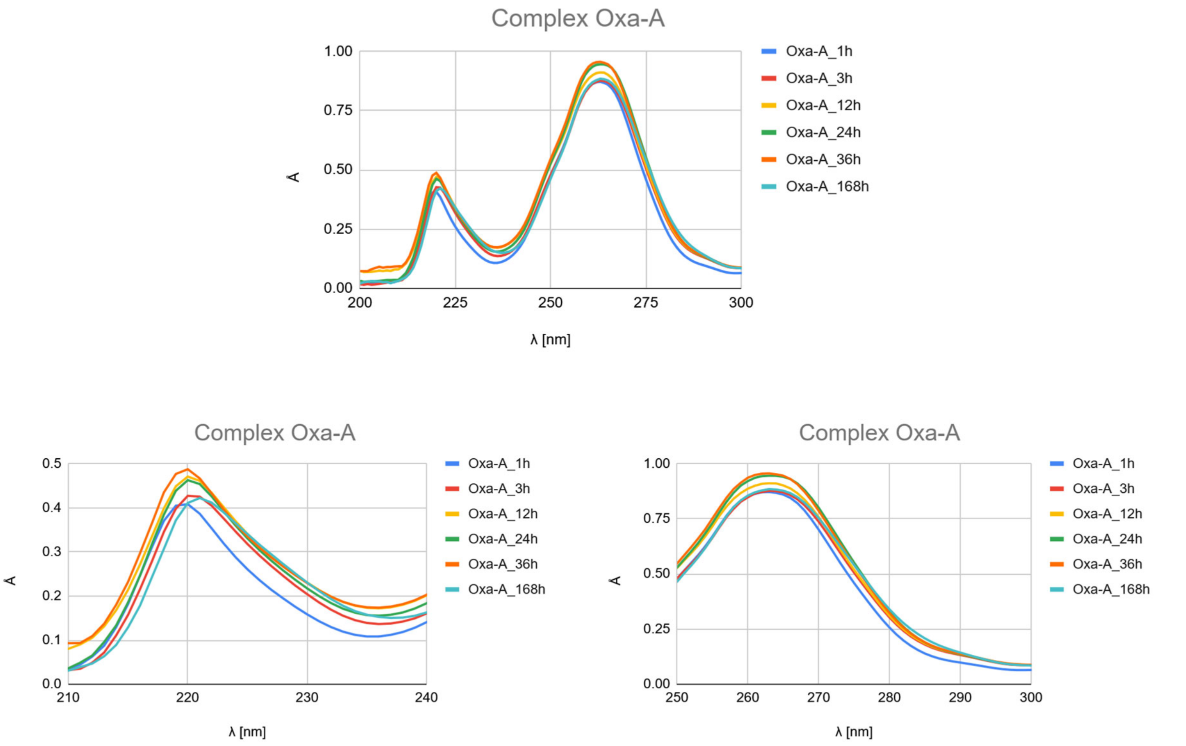

Figure S19. *UV–Vis spectra of a mixed solution of Adenosine and Oxaliplatin recorded after 1, 3, 12, 24, 36, and 168 hours of incubation at 37 °C.*

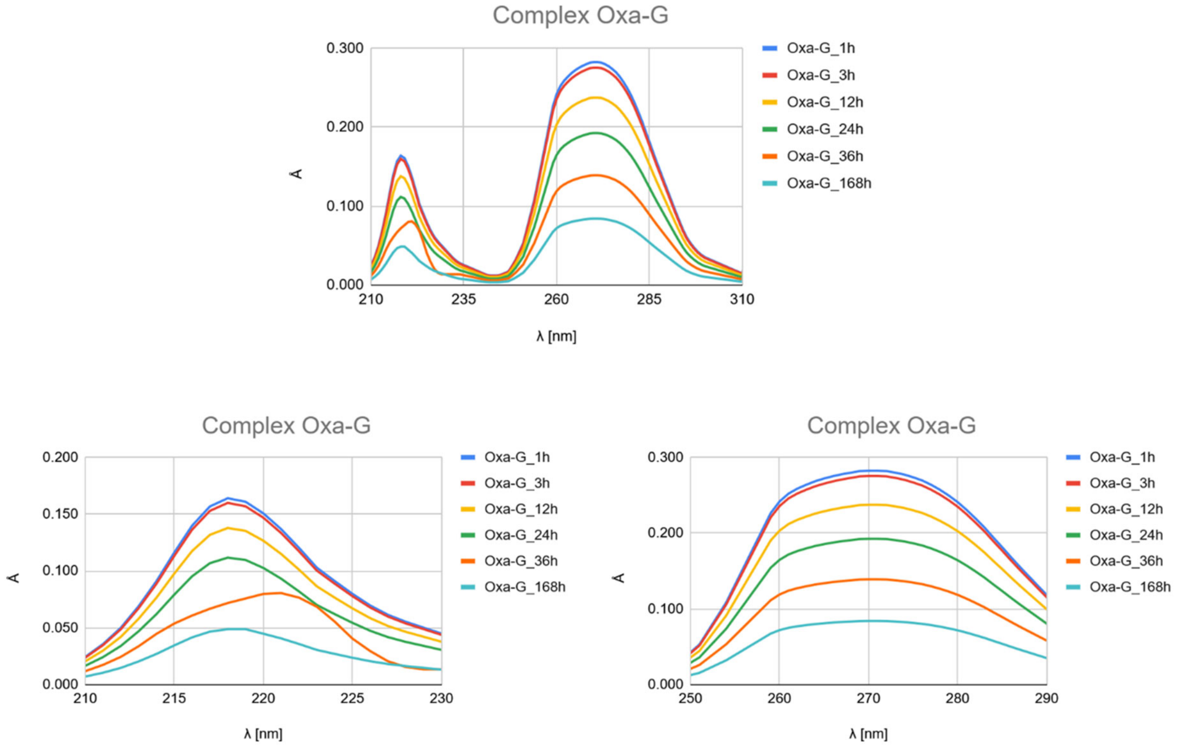

Figure S20. *UV–Vis spectra of a mixed solution of Guanosine and Oxaliplatin recorded after 1, 2, 3, 4, 5, and 168 hours of incubation at 37 °C.*

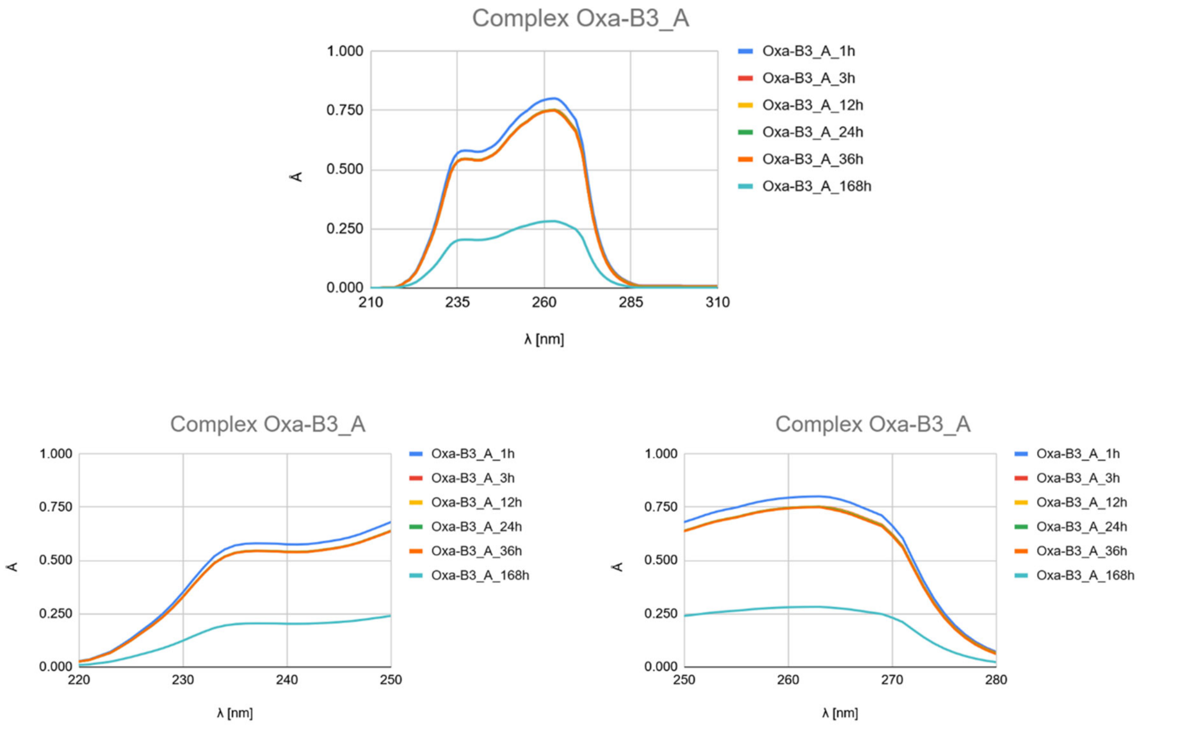

Figure S21. *UV–Vis spectra of a mixed solution of Nicotinic acid (3-pyridinecarboxylic acid, B3_A) and Oxaliplatin recorded after 1, 3, 12, 24, 36, and 168 hours of incubation at 37 °C.*

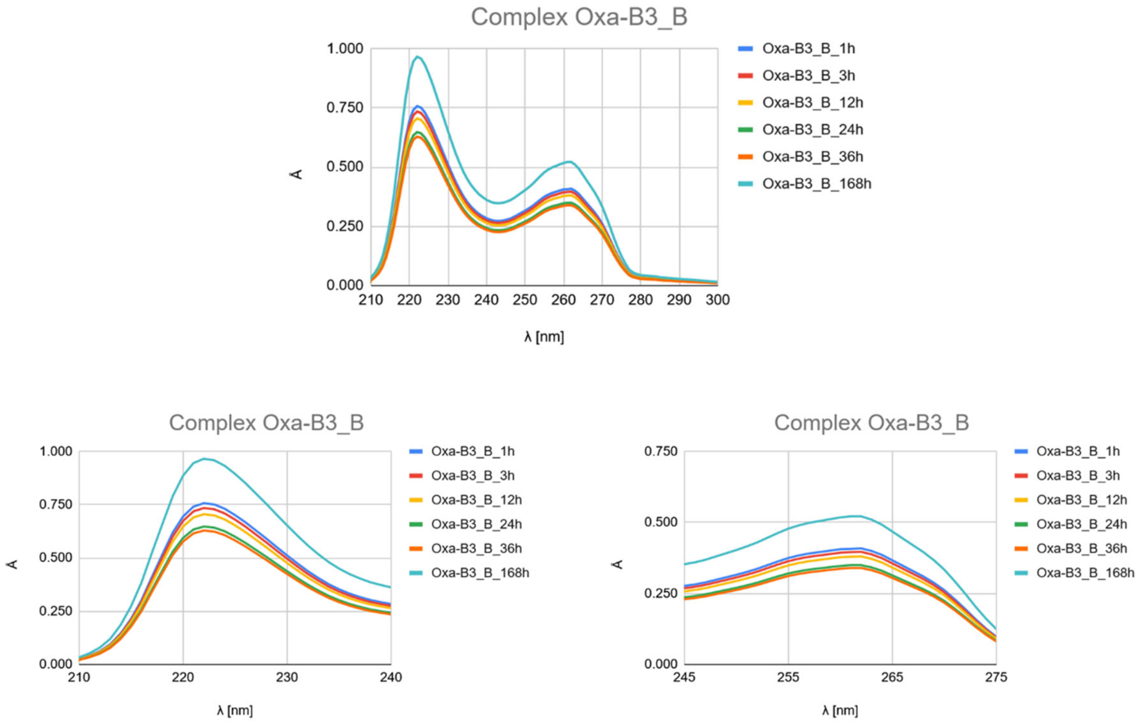

Figure S22. *UV–Vis spectra of a mixed solution of Nicotinamide (pyridine-3-carboxamide, B3_B) and Oxaliplatin recorded after 1, 3, 12, 24, 36, and 168 hours of incubation at 37 °C.*

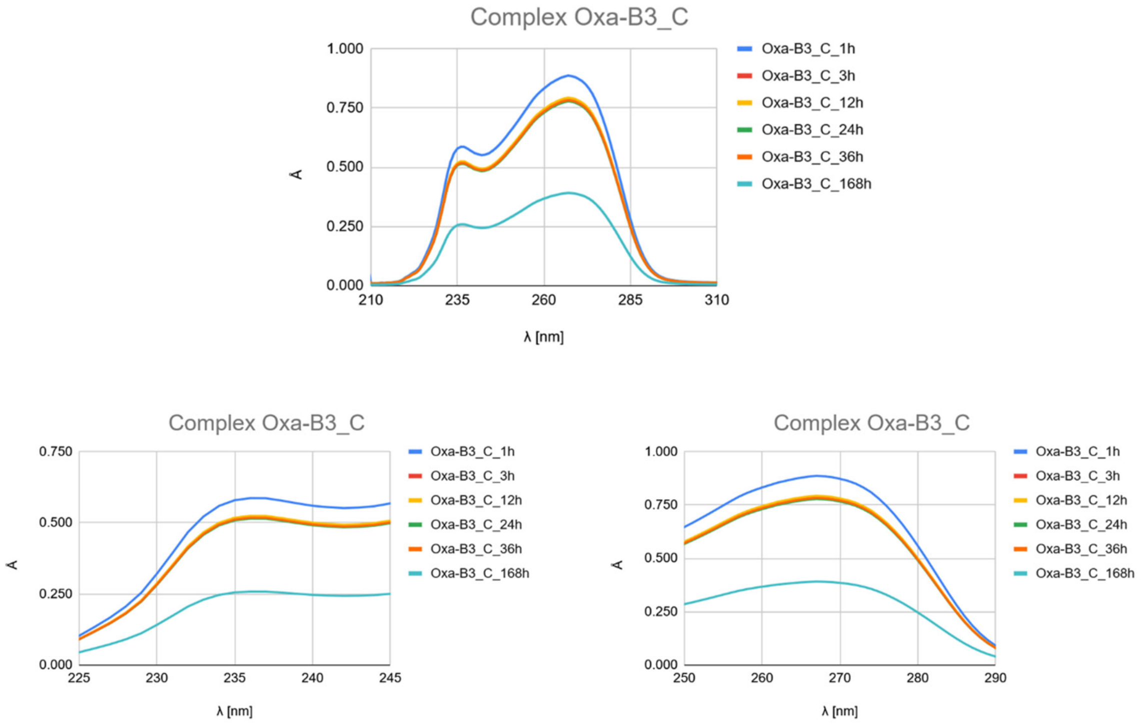

Figure S23. *UV–Vis spectra of a mixed solution of Isonicotinic acid (pyridine-4-carboxylic, B3_C) and Oxaliplatin recorded after 1, 3, 12, 24, 36, and 168 hours of incubation at 37 °C.*

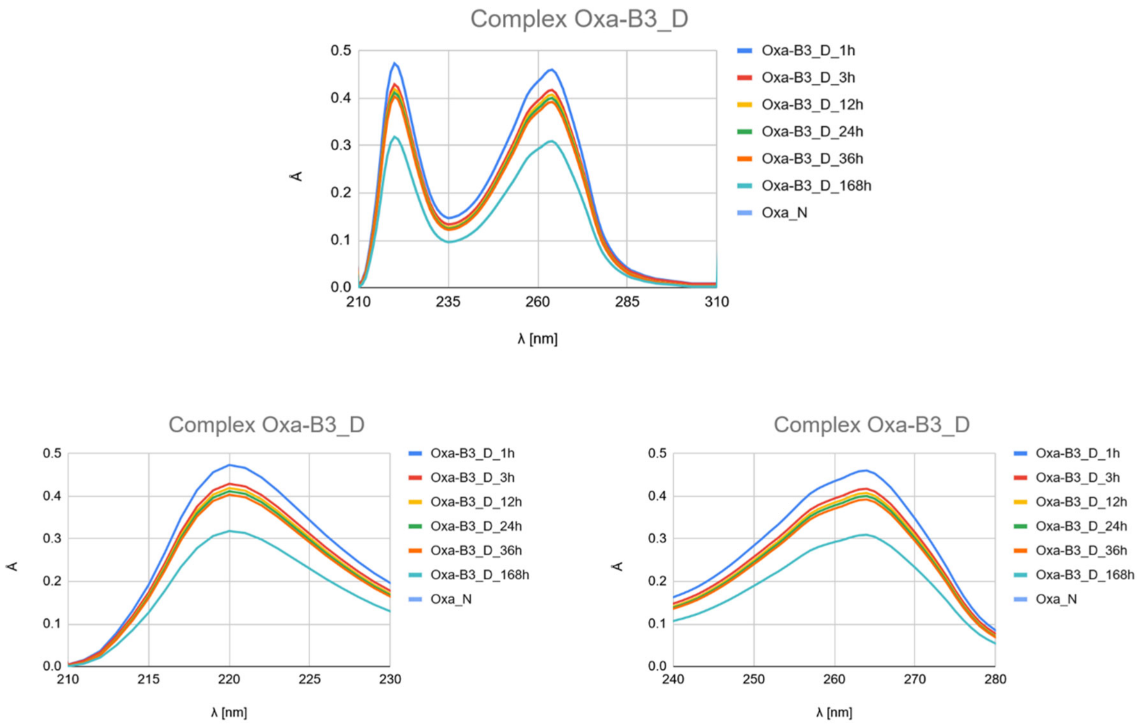

Figure S24. *UV–Vis spectra of a mixed solution of Picolinic acid (pyridine-2-carboxylic, B3_D) and Oxaliplatin recorded after 1, 3, 12, 24, 36, and 168 hours of incubation at 37 °C.*

Methods

B3LYP/6-31G(d,p)/LANL2DZ	MN15/def2-TZVP

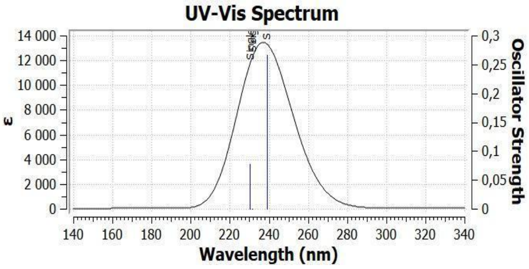

Adenine (A)

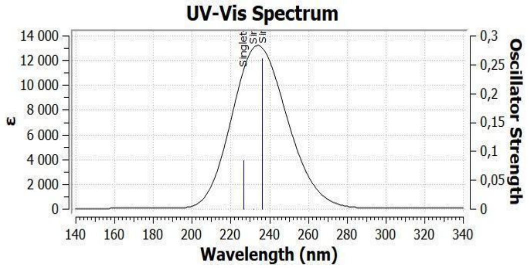

Adenine (A)

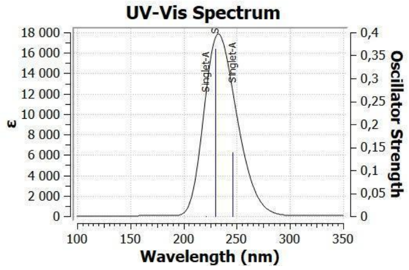

Guanine (G)

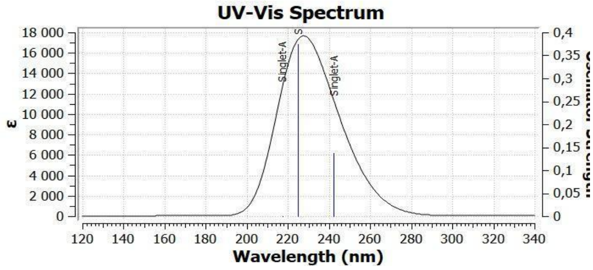

Guanine (G)

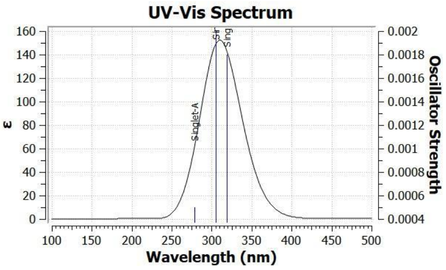

Complex Car_1-A

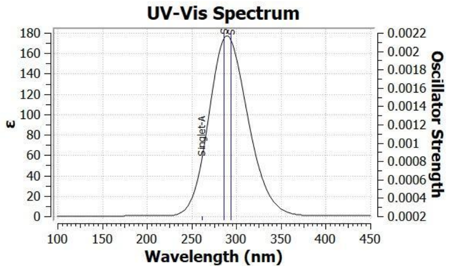

Complex Car_1-A

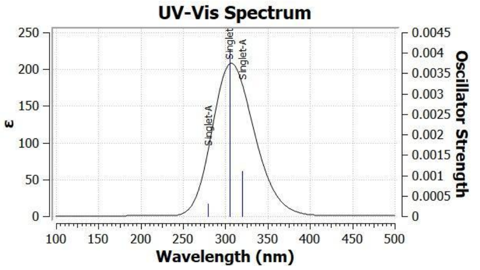

Complex Car_1-G

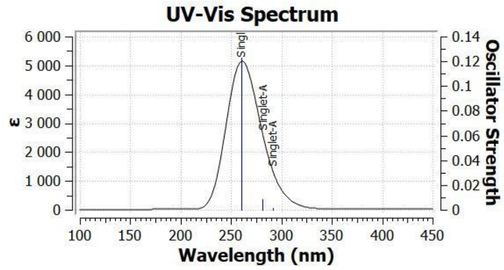

Complex Car_1-G

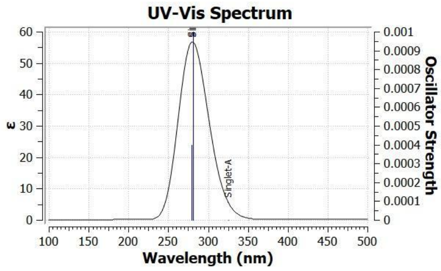

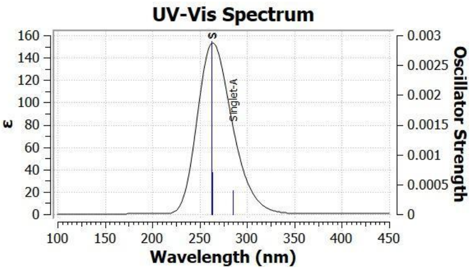

Complex Car_2-A

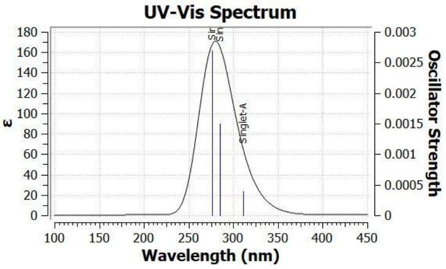

Complex Car_2-A

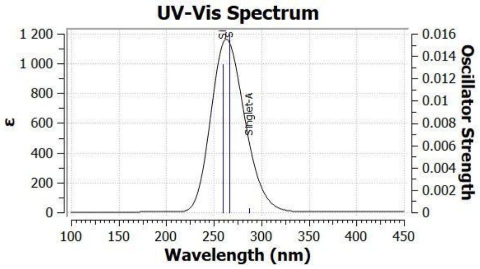

Complex Car_2-G

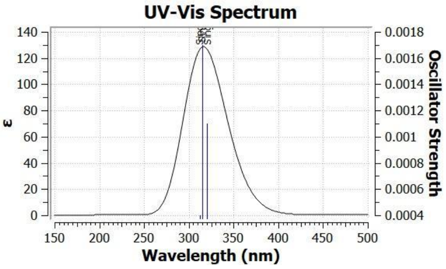

Complex Car_2-G

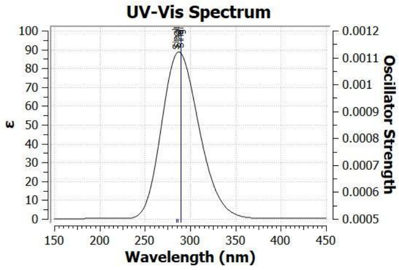

Complex Cis_1-A

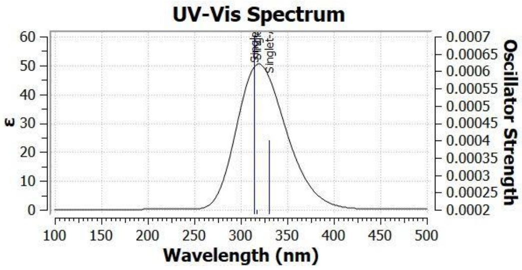

Complex Cis_1-A

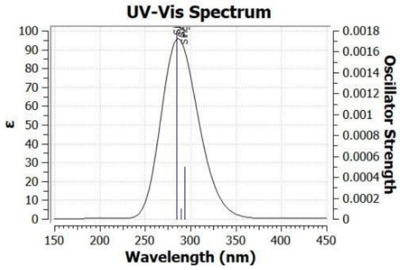

Complex Cis_1-G

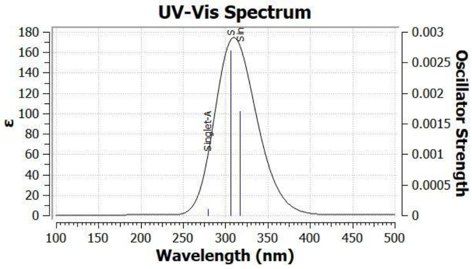

Complex Cis_1-G

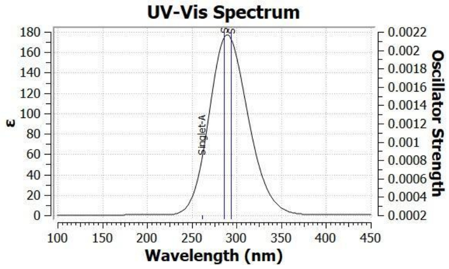

Complex Cis_2-A

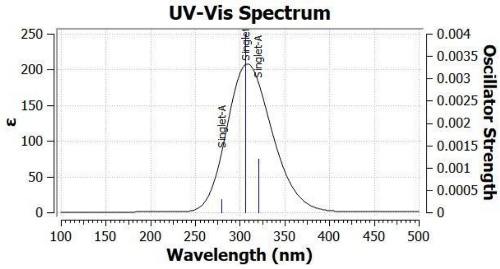

Complex Cis_2-A

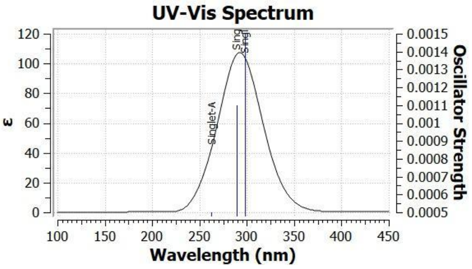

Complex Cis_2-G

Complex Cis_2-G

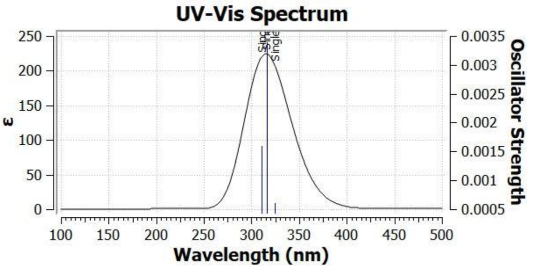

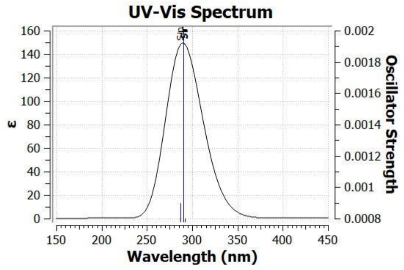

Complex Oxa_1-A

Complex Oxa_1-A

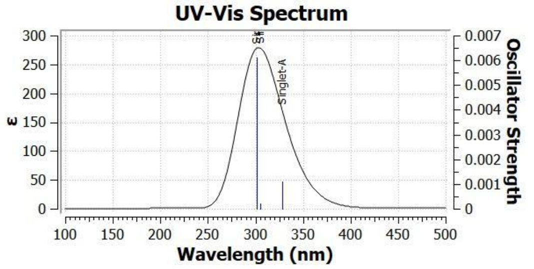

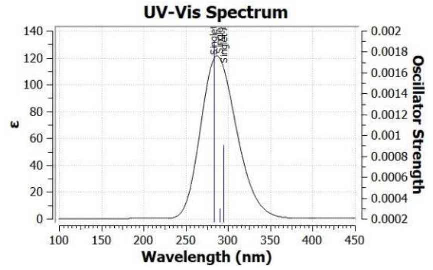

Complex Oxa_1-G

Complex Oxa_1-G

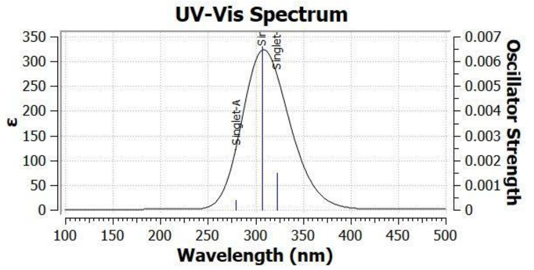

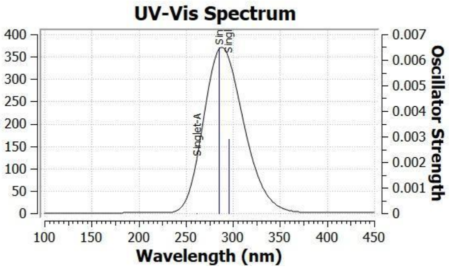

Complex Oxa_2-A

Complex Oxa_2-A

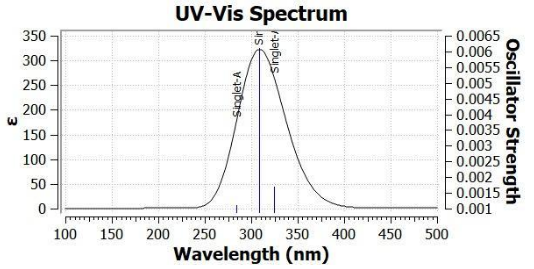

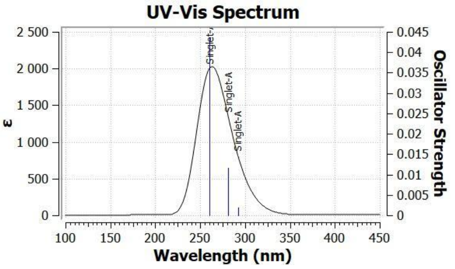

Complex Oxa_2-G

Complex Oxa_2-G

Figure S25. *Calculated UV–Vis absorption spectra of selected nucleobases, Adenine (A) and Guanine (G) and their complexes with the first and second hydrolysis products of Cisplatin (Cis_1 and Cis_2), Carboplatin (Car_1 and Car_2), and Oxaliplatin (Oxa_1 and Oxa_2). The geometries were optimized at the B3LYP/6-31G(d,p)/LANL2DZ and MN15/def2-TZV levels of theory using the PCM model with water as the solvent. All spectroscopic calculations were performed employing the PBE0 functional.*

Methods	
**B3LYP/6-31G(d,p)/LANL2DZ**	**MN15/def2-TZVP**
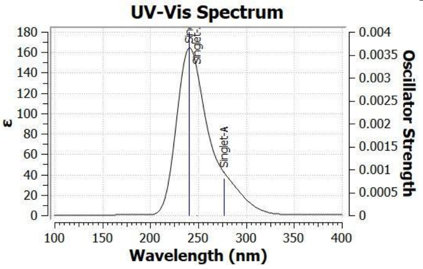<br>Nicotinic acid (B3_A)	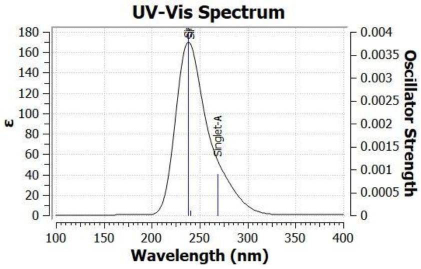<br>Nicotinic acid (B3_A)
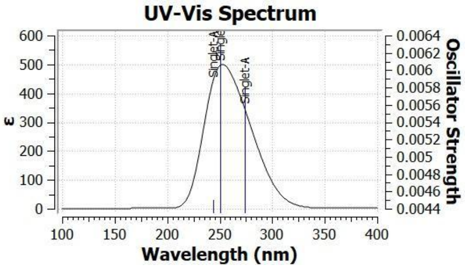<br>Nicotinamide (B3_B)	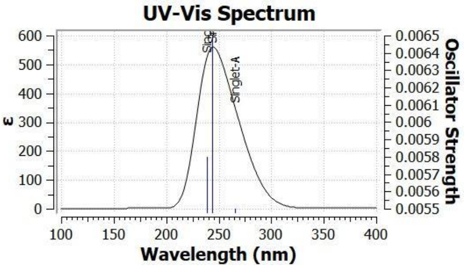<br>Nicotinamide (B3_B)
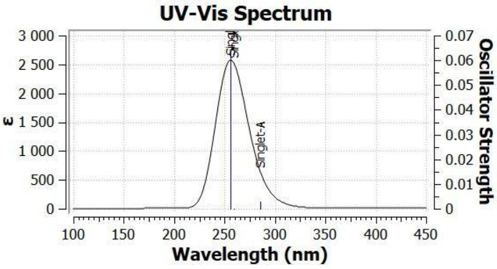<br>Isonicotinic acid (B3_C)	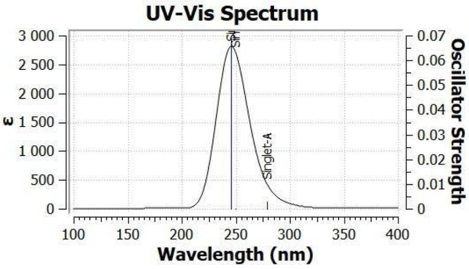<br>Isonicotinic acid (B3_C)
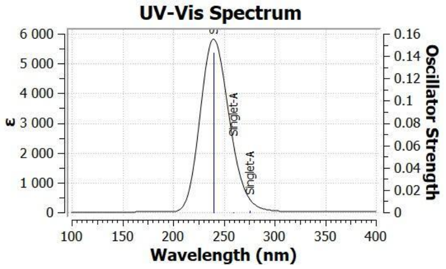<br>Picolinic acid (B3_D)	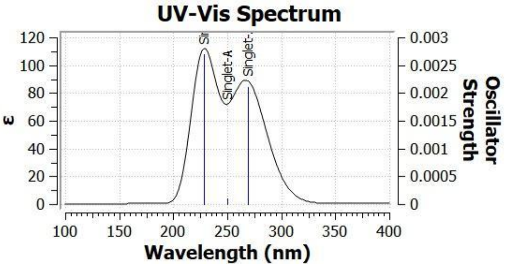<br>Picolinic acid (B3_D)
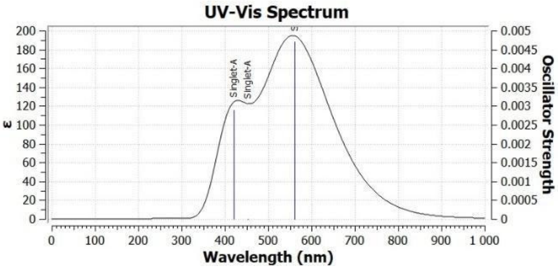<br>Car_1	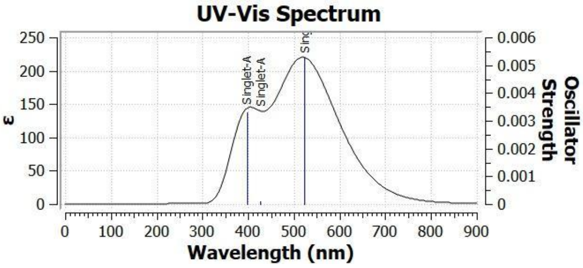<br>Car_1

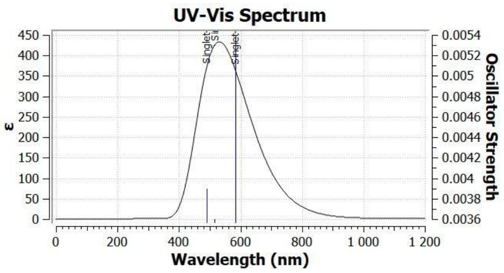

Car_2

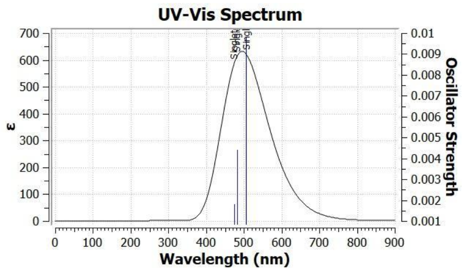

Car_2

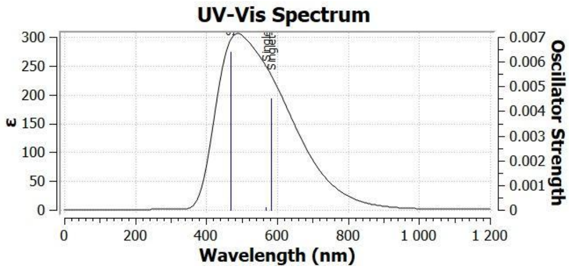

Cis_1

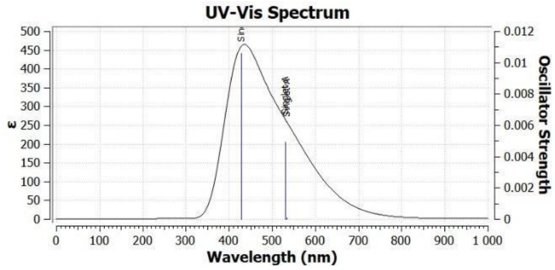

Cis_1

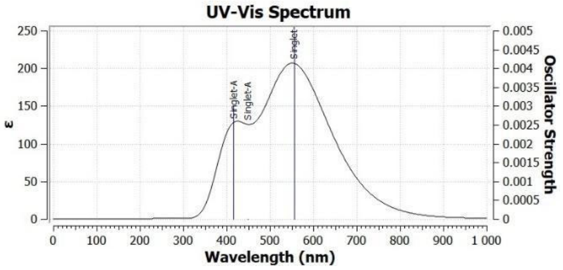

Cis_2

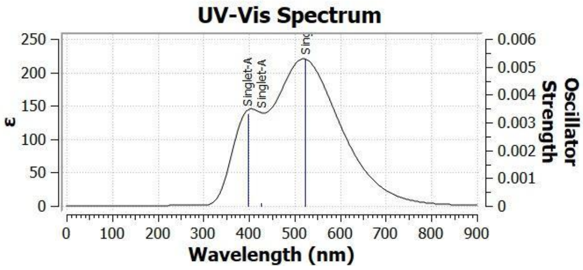

Cis_2

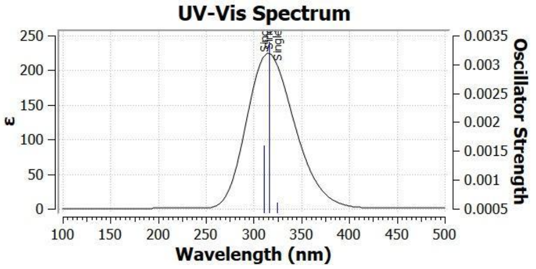

Oxa_1

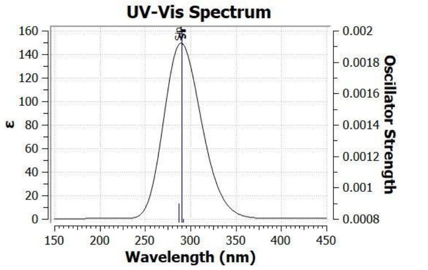

Oxa_1

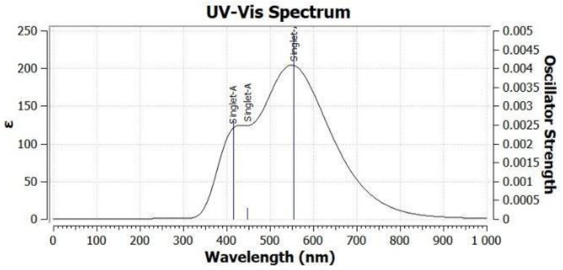

Oxa_2

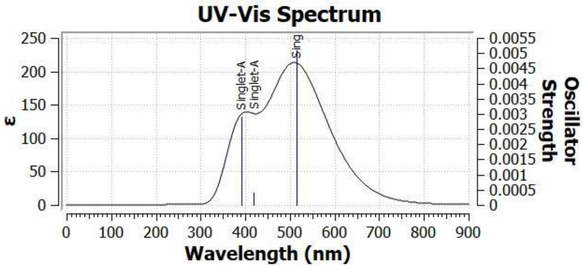

Oxa_2

Figure S26. *Calculated UV–Vis absorption spectra of pyridine derivatives: Nicotinic acid (B3_A), Nicotinamide (B3_B), Isonicotinic acid (B3_C), and Picolinic acid (B3_D), selected nucleobases, Adenine (A) and Guanine (G) and the first and second hydrolysis products of Cisplatin (Cis_1 and Cis_2), Carboplatin (Car_1 and Car_2) and Oxaliplatin (Oxa_1 and Oxa_2). The geometries were optimized at the B3LYP/6-31G(d,p)/LANL2DZ and MN15/def2-TZV levels of theory using the PCM model with water as the solvent. All spectroscopic calculations were performed employing the PBE0 functional.*

Methods

B3LYP/6-31G(d,p)/LANL2DZ | **MN15/def2-TZVP**

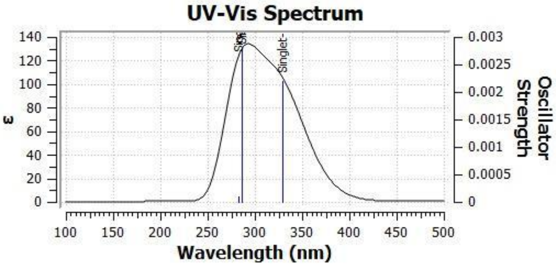

Car_1-B3-A

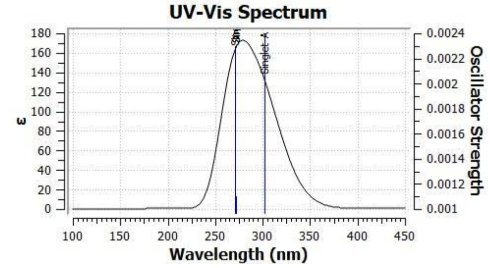

Car_1-B3-A

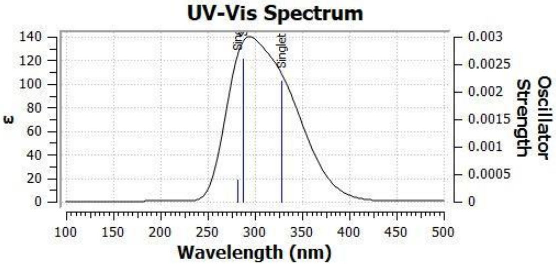

Car_1-B3-B

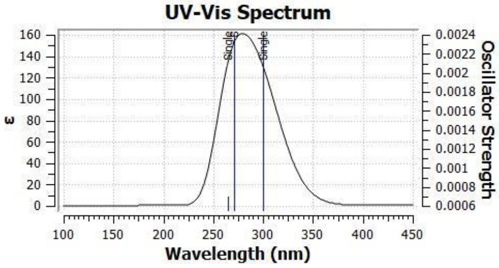

Car_1-B3-B

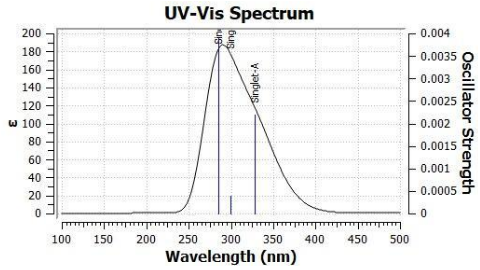

Car_1-B3-C

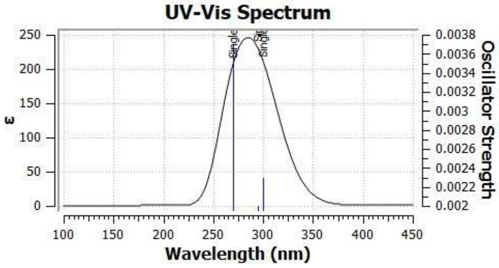

Car_1-B3-C

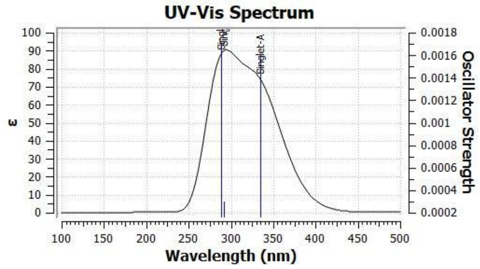

Car_1-B3-D

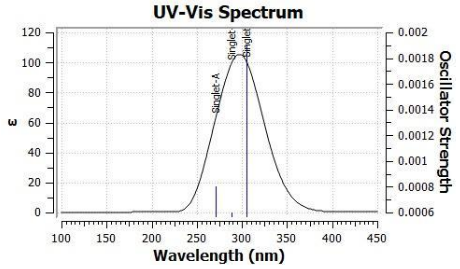

Car_1-B3-D

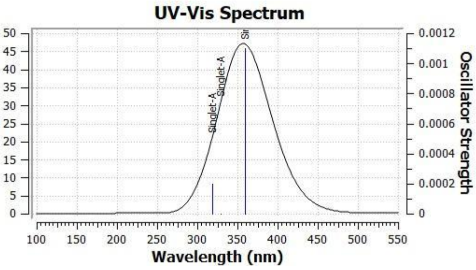

Car_2-B3-A

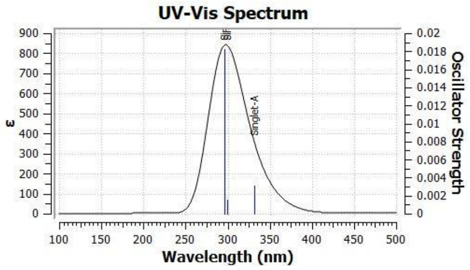

Car_2-B3-A

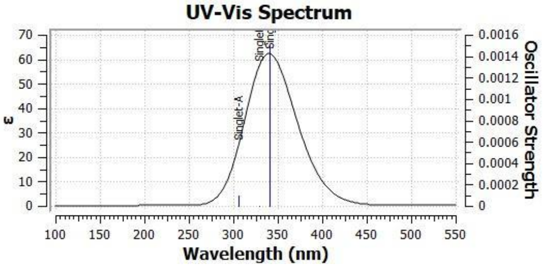

Car_2-B3-B

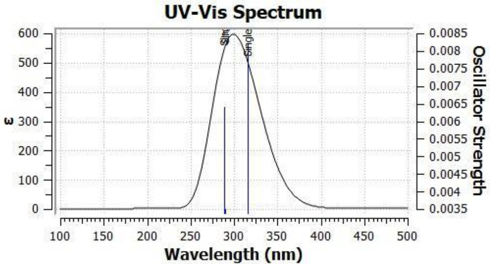

Car_2-B3-B

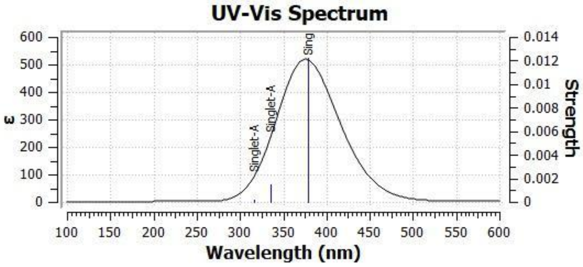

Car_2-B3-C

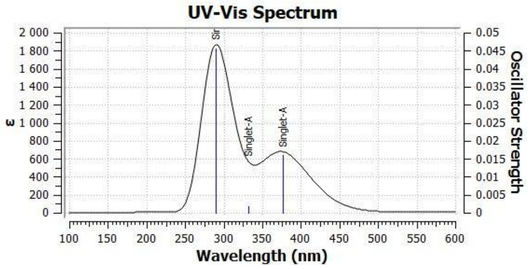

Car_2-B3-C

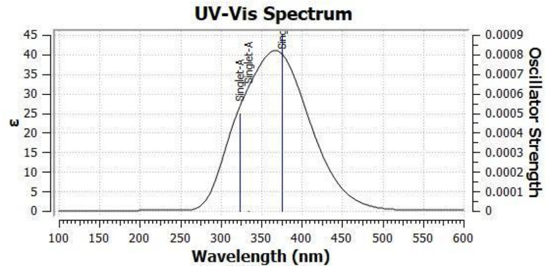

Car_2-B3-D

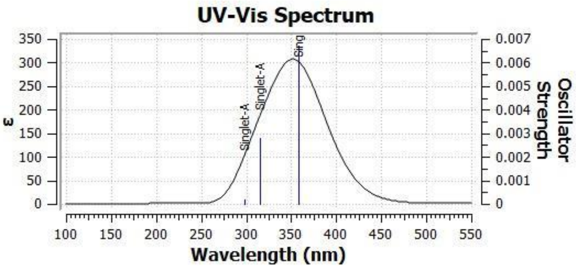

Car_2-B3-D

Figure S27. *Calculated UV–Vis absorption spectra of the first and second product of hydrolysis of Carboplatin (Car_1 and Car_2) complexes with pyridine derivatives: Nicotinic acid (B3_A), Nicotinamide (B3_B), Isonicotinic acid (B3_C), and Picolinic acid (B3_D). The geometries were optimized at the B3LYP/6-31G(d,p)/LANL2DZ and MN15/def2-TZV levels of theory using the PCM model with water as the solvent. All spectroscopic calculations were performed employing the PBE0 functional.*

Methods

B3LYP/6-31G(d,p)/LANL2DZ

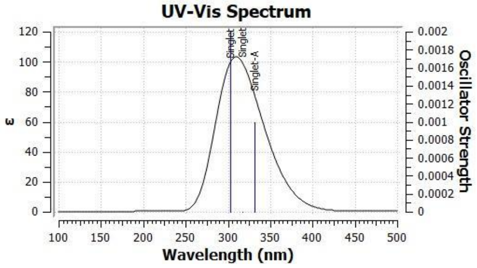

Cis_1-B3-A

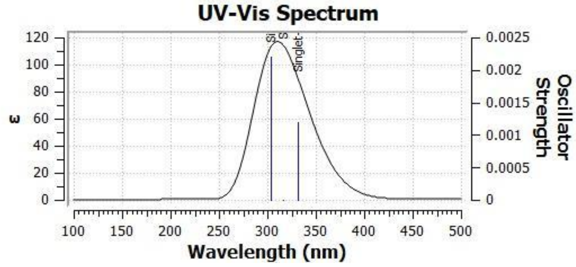

Cis_1-B3-B

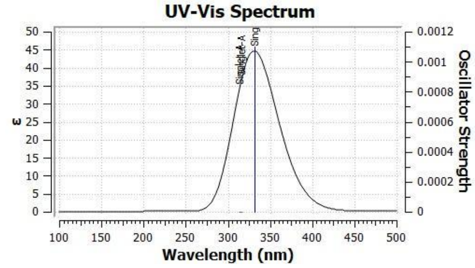

Cis_1-B3-C

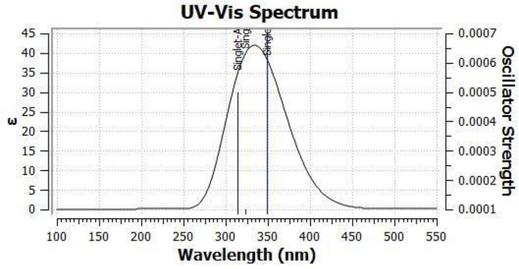

Cis_1-B3-D

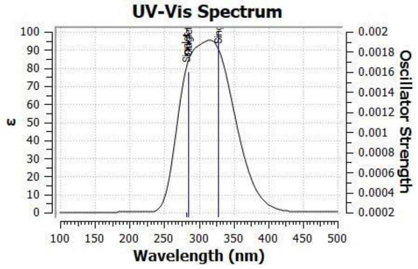

Cis_2-B3-A

MN15/def2-TZVP

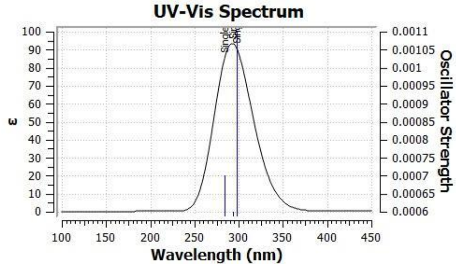

Cis_1-B3-A

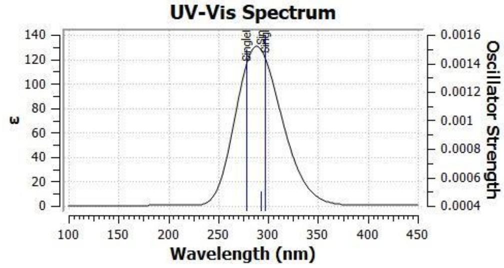

Cis_1-B3-B

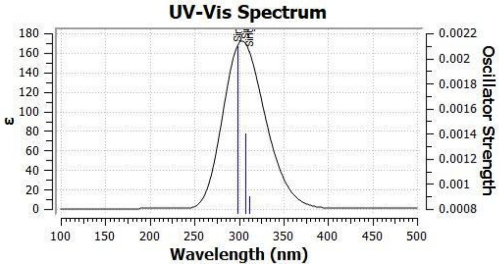

Cis_1-B3-C

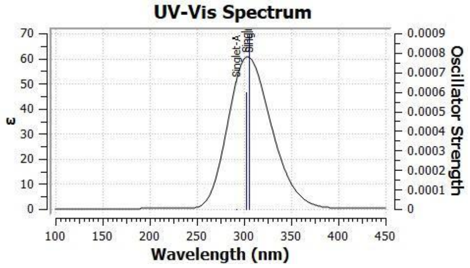

Cis_1-B3-D

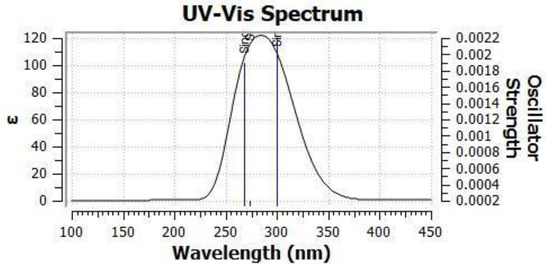

Cis_2-B3-A

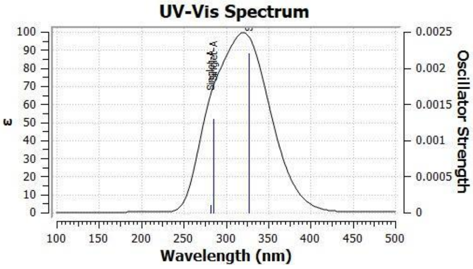

Cis_2-B3-B

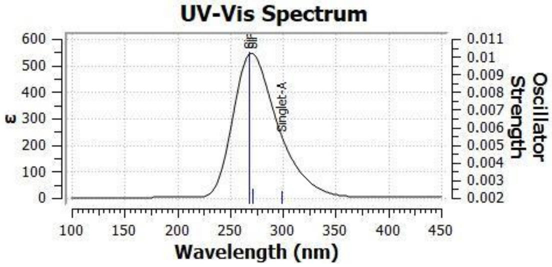

Cis_2-B3-B

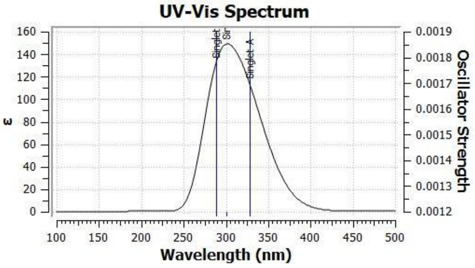

Cis_2-B3-C

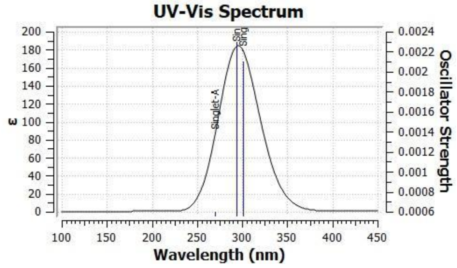

Cis_2-B3-C

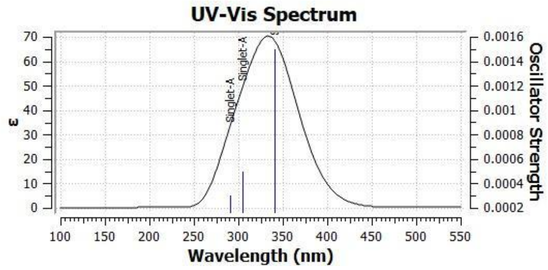

Cis_2-B3-D

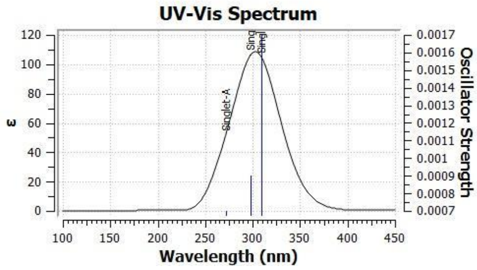

Cis_2-B3-D

Figure S28. *Calculated UV–Vis absorption spectra of the first and second product of hydrolysis of Cisplatin (Cis_1 and Cis_2) complexes with pyridine derivatives: Nicotinic acid (B3_A), Nicotinamide (B3_B), Isonicotinic acid (B3_C), and Picolinic acid (B3_D). The geometries were optimized at the B3LYP/6-31G(d,p)/LANL2DZ and MN15/def2-TZV levels of theory using the PCM model with water as the solvent. All spectroscopic calculations were performed employing the PBE0 functional.*

Methods

B3LYP/6-31G(d,p)/LANL2DZ	MN15/def2-TZVP

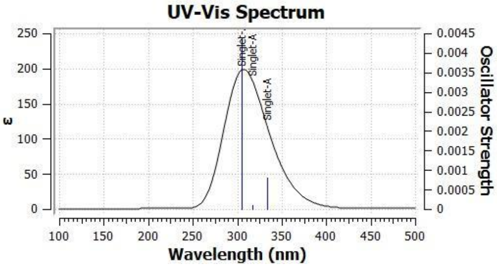

Oxa_1-B3-A

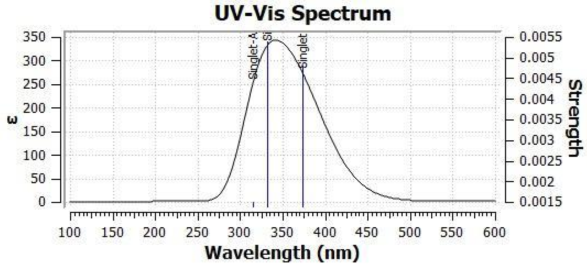

Oxa_1-B3-A

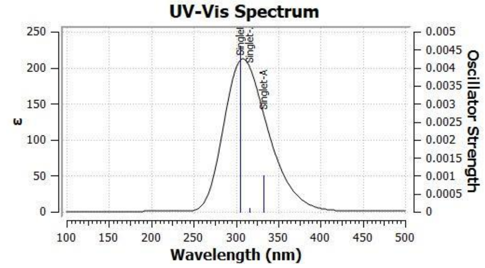

Oxa_1-B3-B

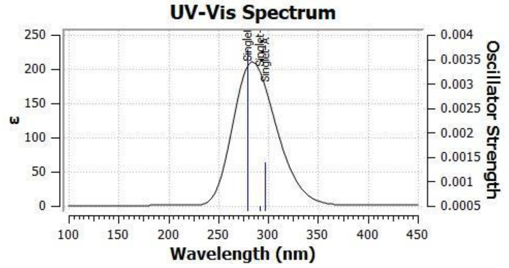

Oxa_1-B3-B

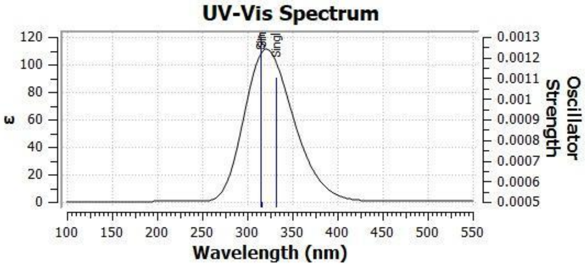

Oxa_1-B3-C

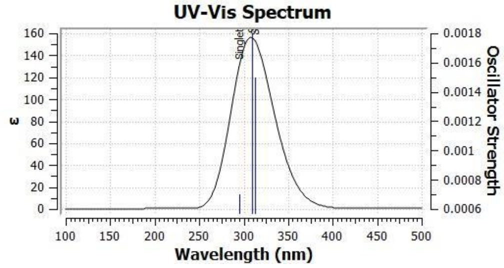

Oxa_1-B3-C

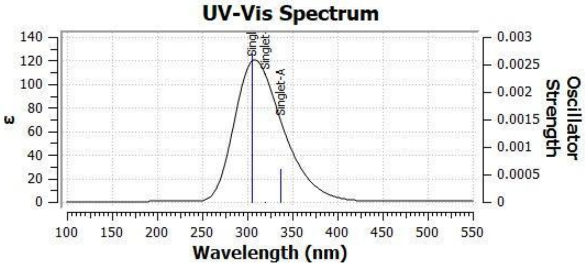

Oxa_1-B3-D

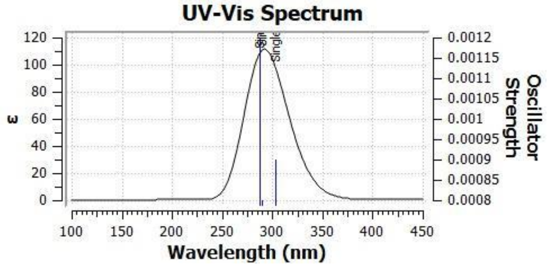

Oxa_1-B3-D

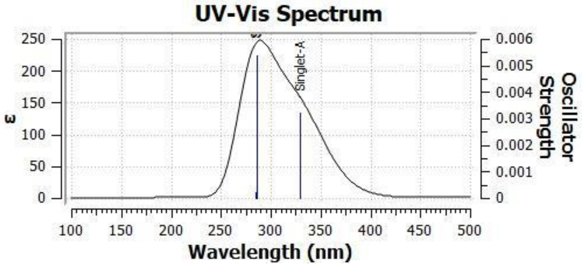

Oxa_2-B3-A

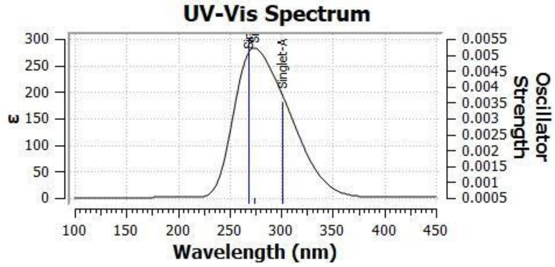

Oxa_2-B3-A

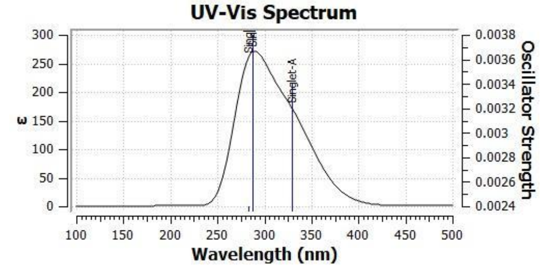

Oxa_2-B3-B

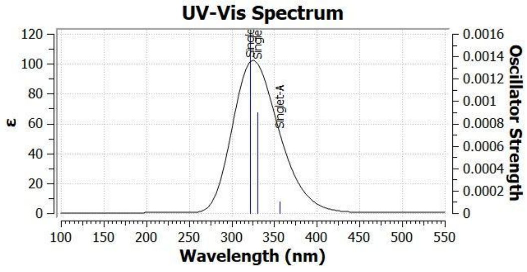

Oxa_2-B3-B

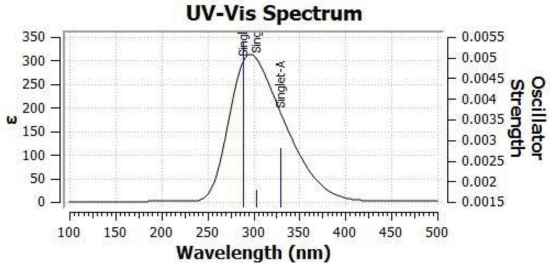

Oxa_2-B3-C

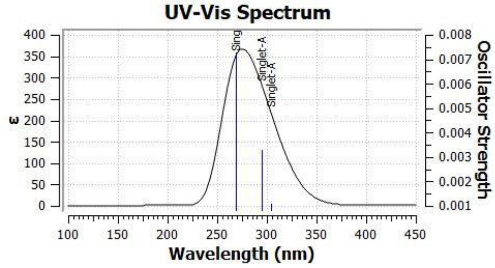

Oxa_2-B3-C

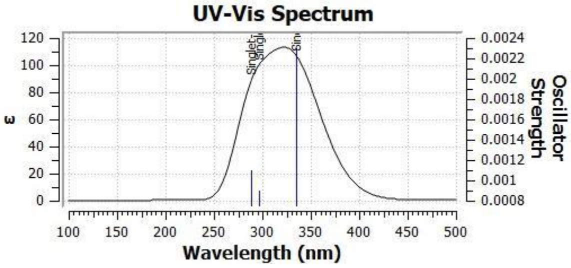

Oxa_2-B3-D

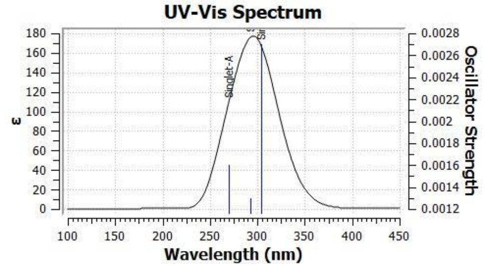

Oxa_2-B3-D

Figure S29. *Calculated UV–Vis absorption spectra of the first and second product of hydrolysis of Oxaliplatin (Oxa_1 and Oxa_2) complexes with pyridine derivatives: Nicotinic acid (B3_A), Nicotinamide (B3_B), Isonicotinic acid (B3_C), and Picolinic acid (B3_D). The geometries were optimized at the B3LYP/6-31G(d,p)/LANL2DZ and MN15/def2-TZV levels of theory using the PCM model with water as the solvent. All spectroscopic calculations were performed employing the PBE0 functional.*

Methods	
**B3LYP/6-31G(d,p)/ LANL2DZ**	**MN15/def2-TZVP**
**HOMO** **LUMO**	**HOMO** **LUMO**
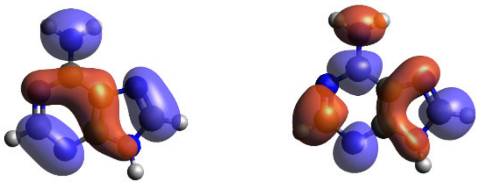 Adenine (A)	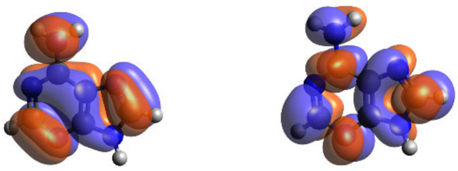 Adenine (A)
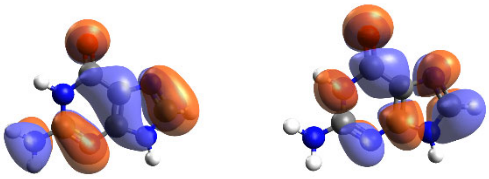 Guanine (G)	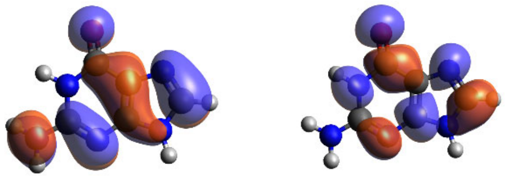 Guanine (G)
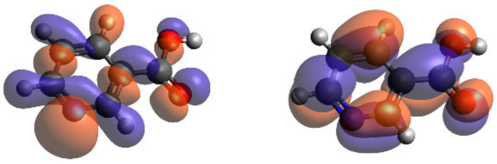 Nicotinic acid (B3_A)	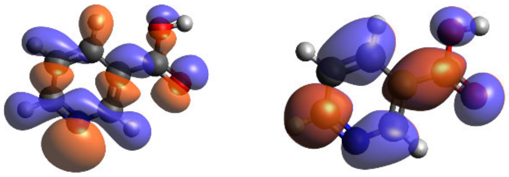 Nicotinic acid (B3_A)
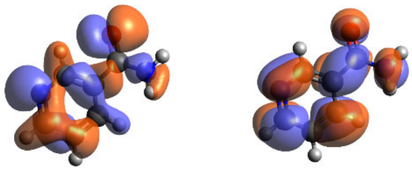 Nicotinamide (B3_B)	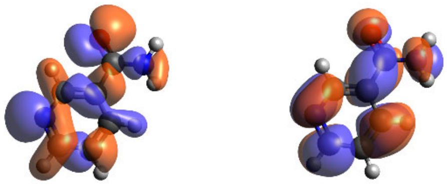 Nicotinamide (B3_B)
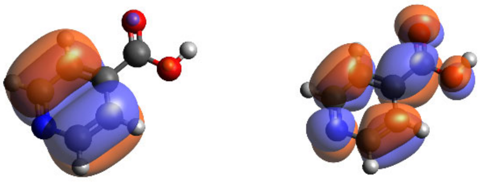 Isonicotinic acid (B3_C)	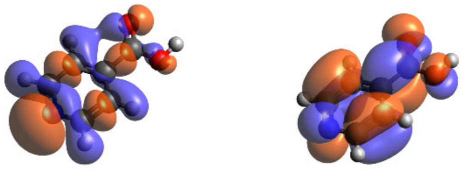 Isonicotinic acid (B3_C)

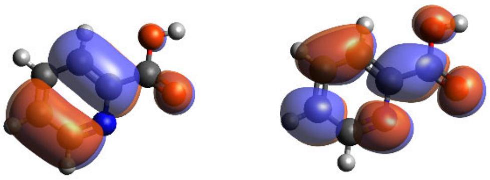

Picolinic acid (B3_D)

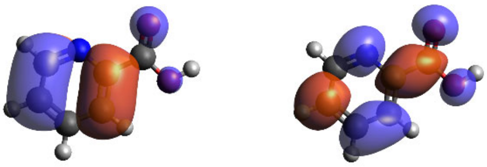

Picolinic acid (B3_D)

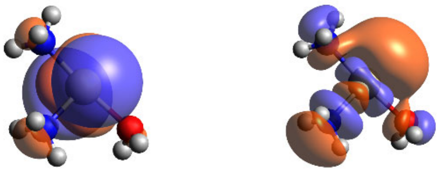

Carboplatin (Car_1)

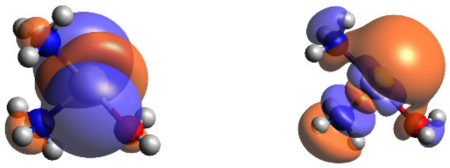

Carboplatin (Car_1)

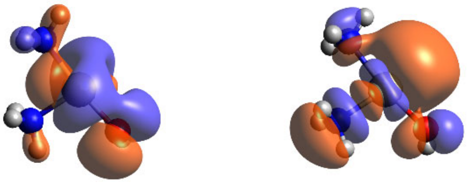

Carboplatin (Car_2)

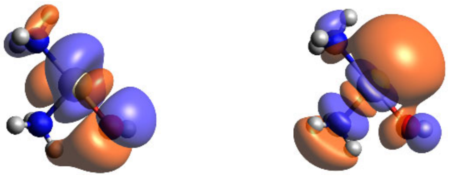

Carboplatin (Car_2)

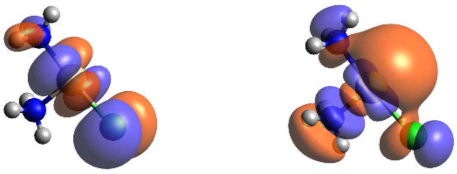

Cisplatin (Cis_1)

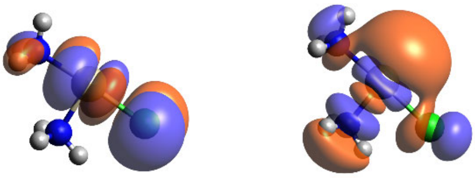

Cisplatin (Cis_1)

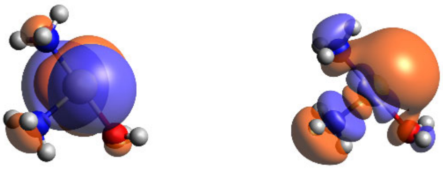

Cisplatin (Cis_2)

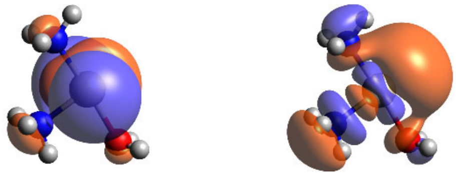

Cisplatin (Cis_2)

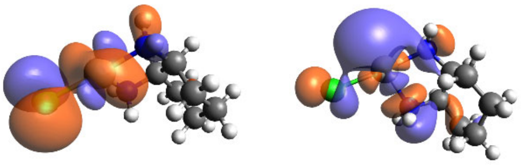

Oxaliplatin (Oxa_1)

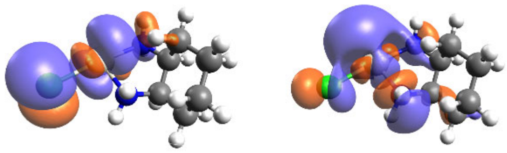

Oxaliplatin (Oxa_1)

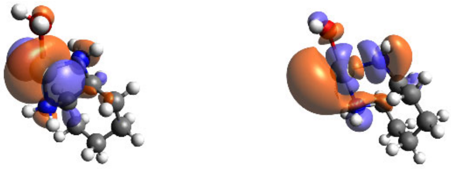

Oxaliplatin (Oxa_2)

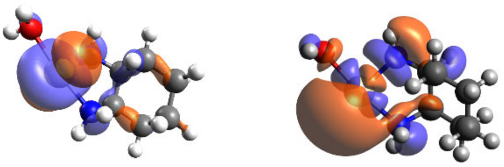

Oxaliplatin (Oxa_2)

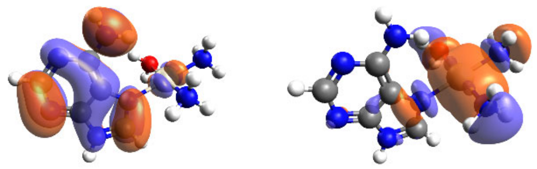

Complex Car_1-A

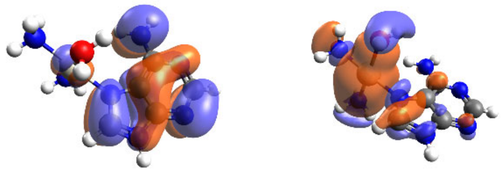

Complex Car_1-A

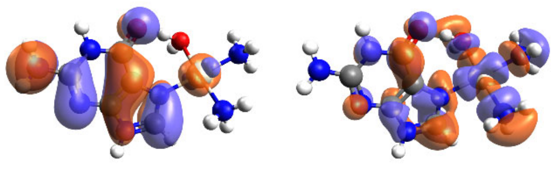

Complex Car_1-G

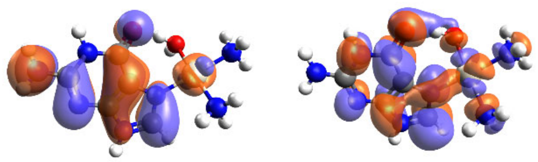

Complex Car_1-G

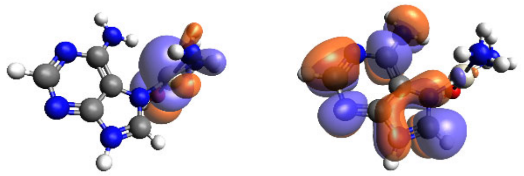

Complex Car_2-A

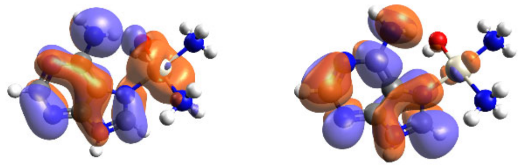

Complex Car_2-A

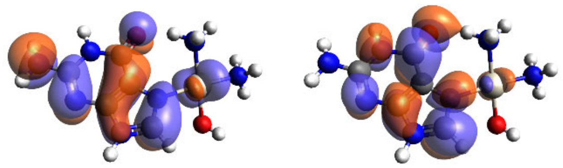

Complex Car_2-G

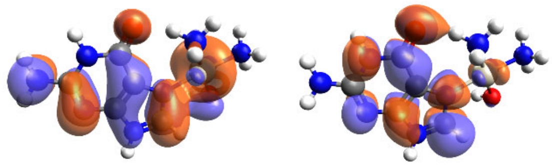

Complex Car_2-G

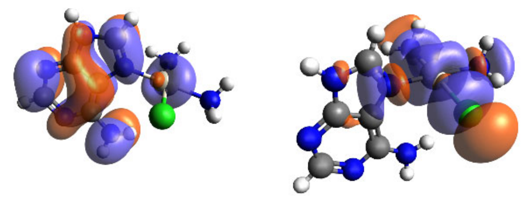

Complex Cis_1-A

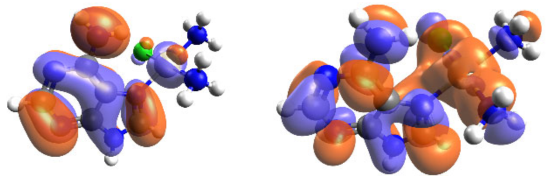

Complex Cis_1-A

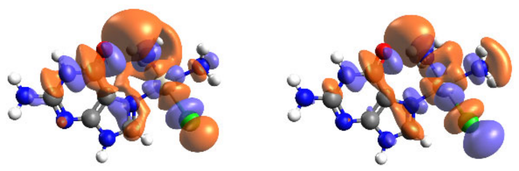

Complex Cis_1-G

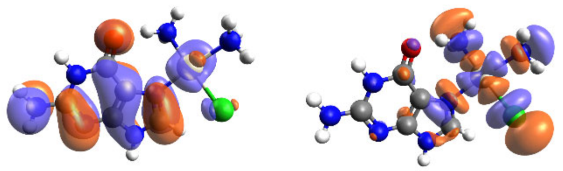

Complex Cis_1-G

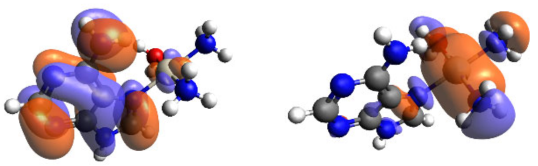

Complex Cis_2-A

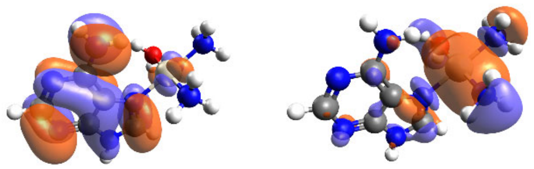

Complex Cis_2-A

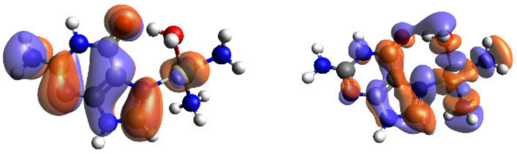

Complex Cis_2-G

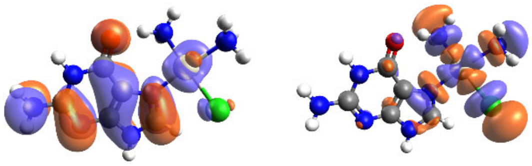

Complex Cis_2-G

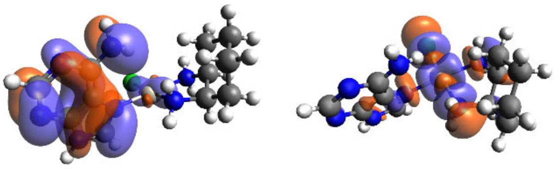

Complex Oxa_1-A

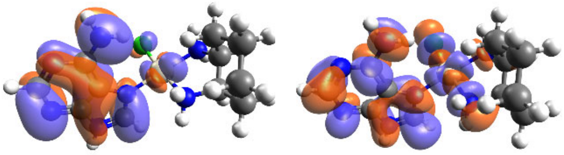

Complex Oxa_1-A

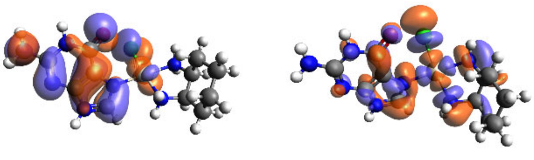

Complex Oxa_1-G

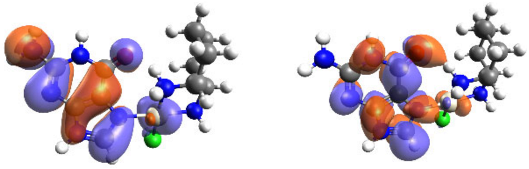

Complex Oxa_1-G

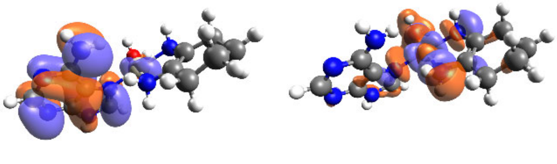

Complex Oxa_2-A

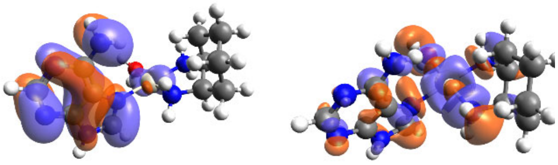

Complex Oxa_2-A

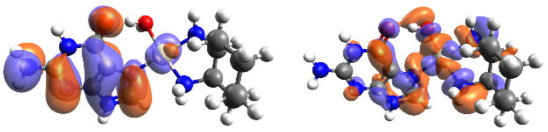

Complex Oxa_2-G

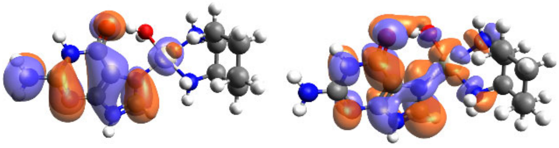

Complex Oxa_2-G

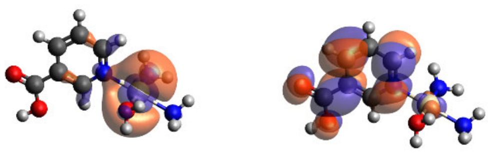

Complex Car_1-B3_A

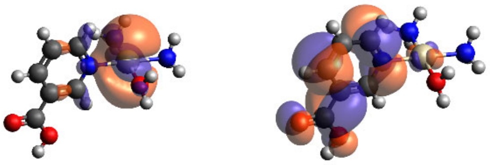

Complex Car_1-B3_A

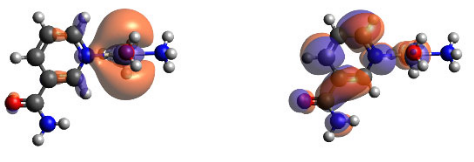

Complex Car_1-B3_B

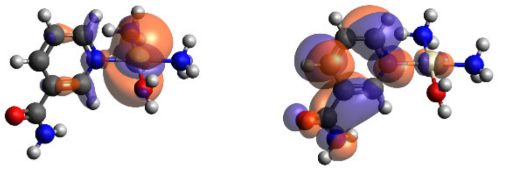

Complex Car_1-B3_B

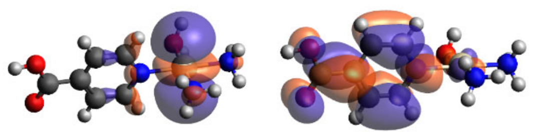

Complex Car_1-B3_C

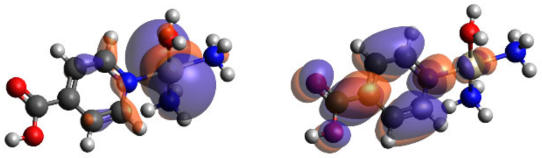

Complex Car_1-B3_C

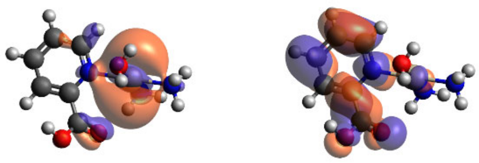

Complex Car_1-B3_D

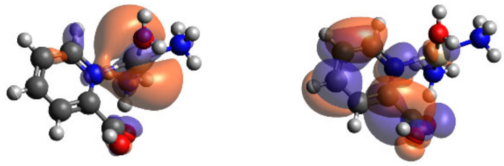

Complex Car_1-B3_D

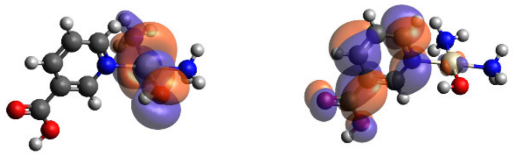

Complex Car_2-B3_A

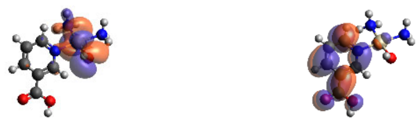

Complex Car_2-B3_A

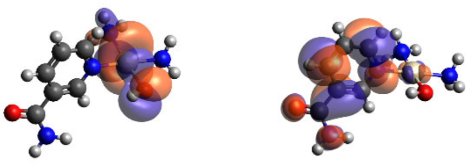

Complex Car_2-B3_B

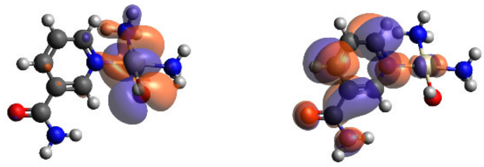

Complex Car_2-B3_B

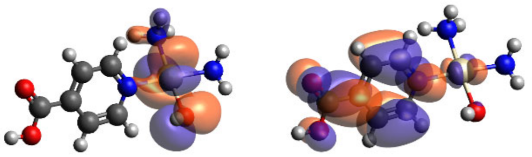

Complex Car_2-B3_C

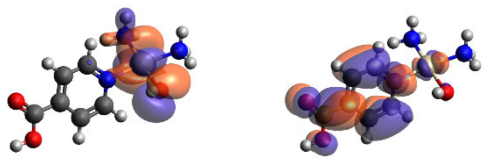

Complex Car_2-B3_C

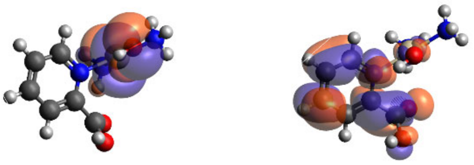

Complex Car_2-B3_D

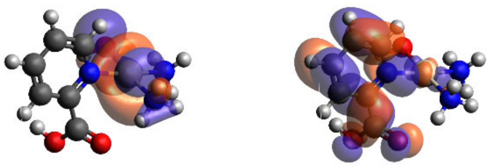

Complex Car_2-B3_D

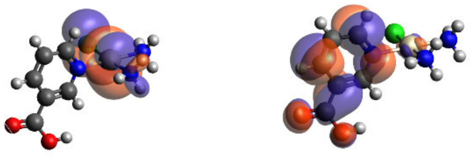

Complex Cis_1-B3_A

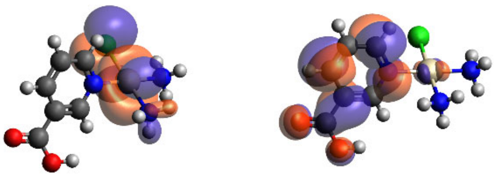

Complex Cis_1-B3_A

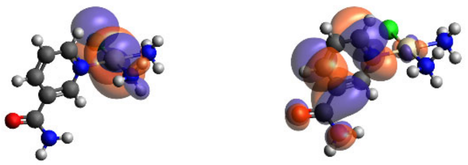

Complex Cis_1-B3_B

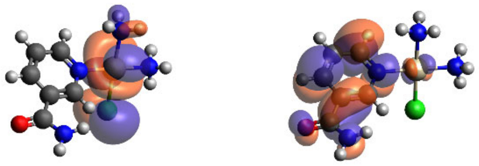

Complex Cis_1-B3_B

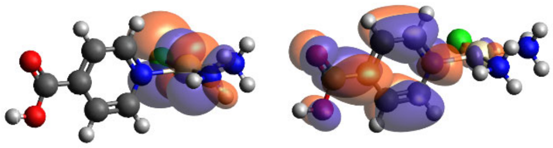

Complex Cis_1-B3_C

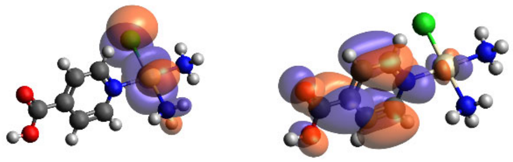

Complex Cis_1-B3_C

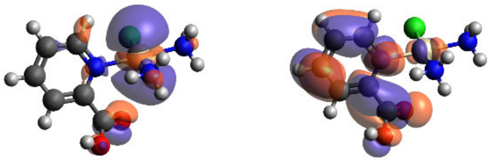

Complex Cis_1-B3_D

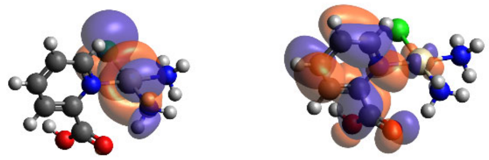

Complex Cis_1-B3_D

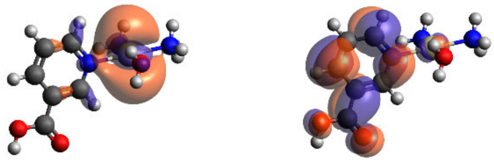

Complex Cis_2-B3_A

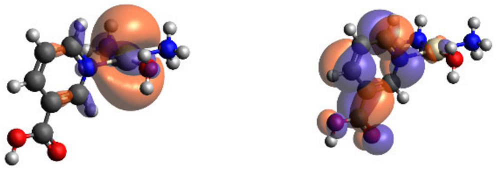

Complex Cis_2-B3_A

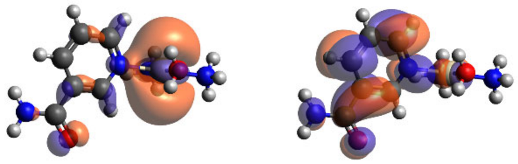

Complex Cis_2-B3_B

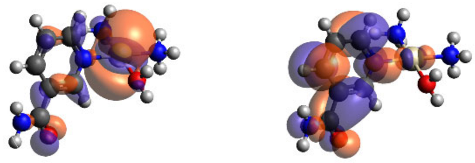

Complex Cis_2-B3_B

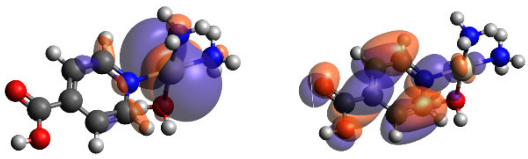

Complex Cis_2-B3_C

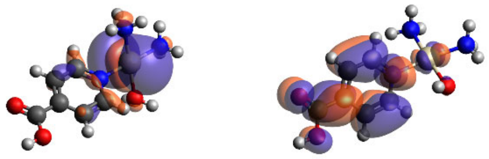

Complex Cis_2-B3_C

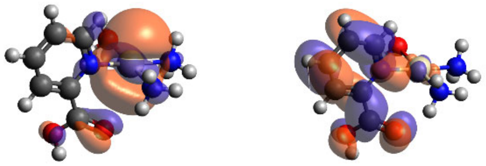

Complex Cis_2-B3_D

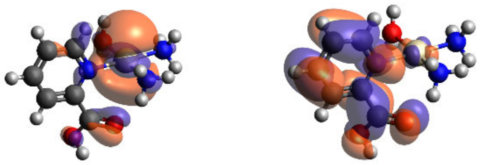

Complex Cis_2-B3_D

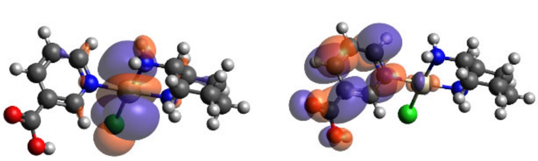

Complex Oxa_1-B3_A

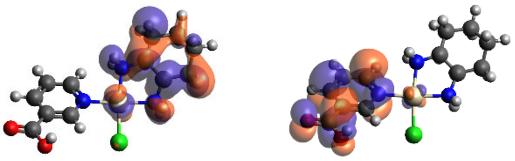

Complex Oxa_1-B3_A

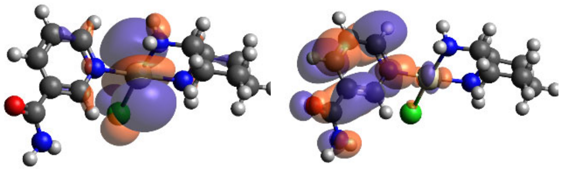

Complex Oxa_1-B3_B

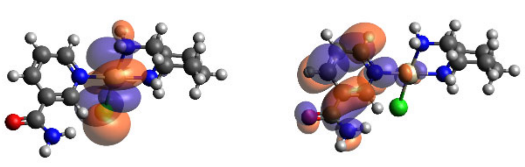

Complex Oxa_1-B3_B

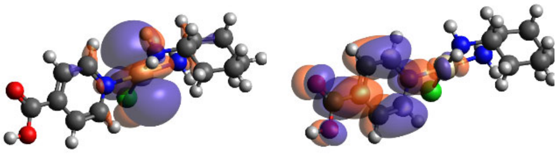

Complex Oxa_1-B3_C

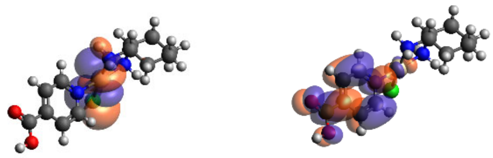

Complex Oxa_1-B3_C

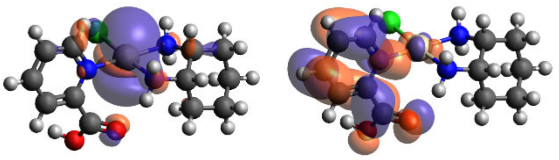

Complex Oxa_1-B3_D

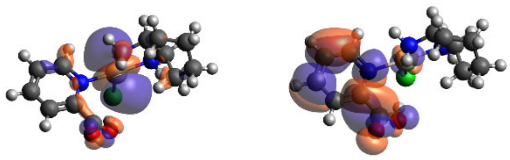

Complex Oxa_1-B3_D

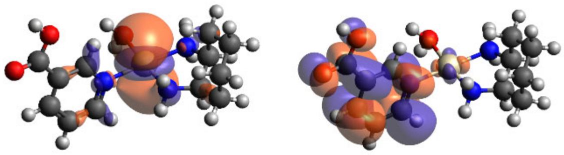

Complex Oxa_2-B3_A

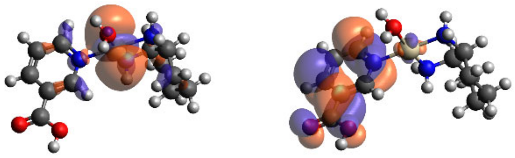

Complex Oxa_2-B3_A

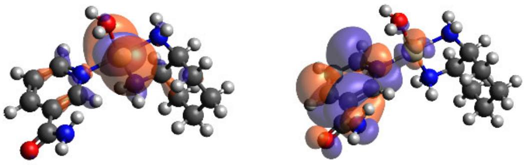

Complex Oxa_2-B3_B

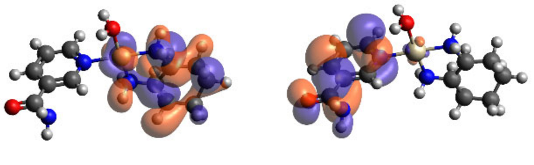

Complex Oxa_2-B3_B

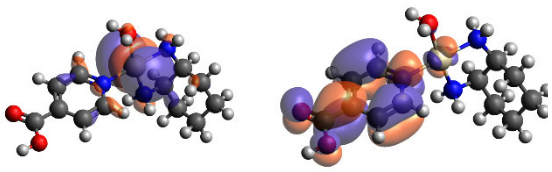

Complex Oxa_2-B3_C

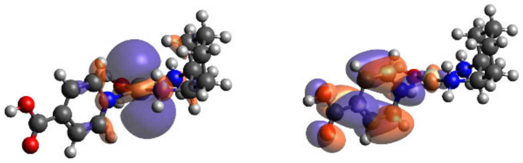

Complex Oxa_2-B3_C

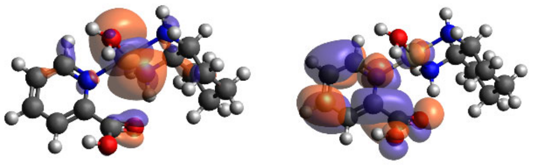

Complex Oxa_2-B3_D

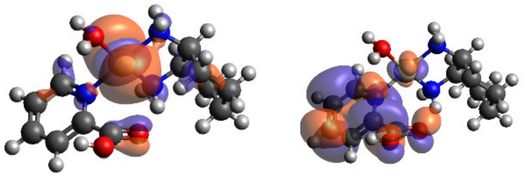

Complex Oxa_2-B3_D

Figure S30. *HOMO LUMO plots of nucleobases (Adenine (A), Guanine (G)) and pyridine derivatives: Nicotinic acid (B3_A), Nicotinamide (B3_B), Isonicotinic acid (B3_C), and Picolinic acid (B3_D), and their complexes with the first and second products of hydrolysis of Carboplatin (Car_1, Car_2), Cisplatin (Cis_1, Cis_2) and Oxaliplatin (Oxa_1, Oxa_2), (isovalue = 0.02 a.u., density = 0.0004 e/Å³).*

Table S1*. HOMO–LUMO energies (in eV) and parameters describing the chemical behaviour of the studied molecules, including the energy gap (ΔE_{gap}), absolute electronegativity (χ), chemical potential (μ), absolute hardness (η), absolute softness (σ), global electrophilicity index (ω), global softness (S), and the maximum additional electronic charge (ΔNmax), calculated at the B3LYP/6-31G(d,p)/LANL2DZ level of theory. The calculated parameters correspond to the complexation reactions between the primary and secondary hydrolysis products of platinum-based drugs: Cisplatin (Cis_1 and Cis_2), Carboplatin (Car_1 and Car_2), and Oxaliplatin (Oxa_1 and Oxa_2) and the tested ligands. The ligands include pyridine derivatives: Nicotinic acid (B3_A), Nicotinamide (B3_B), Isonicotinic acid (B3_C), and Picolinic acid (B3_D), as well as nucleobases: Adenine (A) and Guanine (G). All calculations were performed using the B3LYP/6-31G(d,p)/LANL2DZ computational method.*

B3LYP/6-31G(d,p)												
**STRUCTURE**	**HOMO (eV)**	**LUMO (eV)**	**Energy gap (eV)**	**η (eV)**	**μ**	**σ**	**s**	**χ**	**ω**	**ΔNmax**	**ΔEn**	**Δee**
B3_A	-7.7	-1.5	6.1	3.1	4.6	0.3	1.5	4.6	32.5	0.7	34.0	24.8
B3_B	-7.3	-1.5	5.8	2.9	4.4	0.3	1.5	4.4	28.7	0.8	30.2	21.3
B3_C	-7.7	-1.8	5.9	3.0	4.7	0.3	1.5	4.7	32.8	0.8	34.6	25.2
B3_D	-7.7	-1.6	6.2	3.1	4.7	0.3	1.5	4.7	33.4	0.8	34.9	25.6
Adenine (A)	-6.3	-0.5	5.8	2.9	3.4	0.3	1.5	3.4	16.5	0.6	17.0	10.3
Guanine (G)	-6.0	-0.1	5.9	2.9	3.1	0.3	1.5	3.1	13.7	0.5	13.8	7.7
Car_1	-7.9	-3.4	4.5	2.3	5.7	0.4	1.1	5.7	35.9	1.3	39.3	28.0
Car_2	-7.0	-2.7	4.3	2.2	4.9	0.5	1.1	4.9	25.3	1.1	28.0	18.3
Cis_1	-7.5	-3.2	4.3	2.2	5.4	0.5	1.1	5.4	30.8	1.2	34.0	23.3
Cis_2	-7.9	-3.4	4.5	2.3	5.7	0.4	1.1	5.7	35.9	1.3	39.3	28.0
Oxa_1	-7.5	-3.2	4.3	2.2	5.4	0.5	1.1	5.4	30.8	1.2	34.0	23.3
Oxa_2	-7.8	-3.3	4.5	2.3	5.6	0.4	1.1	5.6	34.7	1.2	38.0	26.9
Car_1-B3_A	-7.5	-2.0	5.6	2.8	4.7	0.4	1.4	4.7	31.3	0.9	33.3	23.8
Car_1-B3_B	-7.5	-1.8	5.8	2.9	4.6	0.3	1.4	4.6	31.3	0.8	33.1	23.8
Car_1-B3_C	-7.6	-2.4	5.2	2.6	5.0	0.4	1.3	5.0	32.2	1.0	34.5	24.6
Car_1-B3_D	-7.5	-2.1	5.4	2.7	4.8	0.4	1.4	4.8	31.0	0.9	33.1	23.5
Car_2-B3_A	-6.5	-1.8	4.7	2.3	4.2	0.4	1.2	4.2	20.2	0.9	22.1	13.8
Car_2-B3_B	-6.5	-1.6	4.9	2.4	4.0	0.4	1.2	4.0	20.0	0.8	21.6	13.5
Car_2-B3_C	-6.4	-2.2	4.2	2.1	4.3	0.5	1.1	4.3	19.6	1.0	21.8	13.2
Car_2-B3_D	-6.3	-1.8	4.5	2.3	4.1	0.4	1.1	4.1	18.7	0.9	20.5	12.4
Cis_1-B3_A	-7.2	-1.9	5.3	2.7	4.5	0.4	1.3	4.5	27.2	0.9	29.1	20.0
Cis_1-B3_B	-7.2	-1.7	5.5	2.8	4.4	0.4	1.4	4.4	27.0	0.8	28.6	19.8
Cis_1-B3_C	-7.2	-2.3	4.9	2.5	4.7	0.4	1.2	4.7	27.5	1.0	29.7	20.3
Cis_1-B3_D	-7.1	-1.9	5.2	2.6	4.5	0.4	1.3	4.5	26.6	0.9	28.6	19.5
Cis_2-B3_A	-7.5	-2.0	5.5	2.8	4.8	0.4	1.4	4.8	31.3	0.9	33.3	23.8
Cis_2-B3_B	-7.5	-1.8	5.7	2.9	4.7	0.3	1.4	4.7	31.2	0.8	33.0	23.7
Cis_2-B3_C	-7.6	-2.4	5.2	2.6	5.0	0.4	1.3	5.0	32.2	1.0	34.6	24.6
Cis_2-B3_D	-7.4	-2.1	5.3	2.6	4.8	0.4	1.3	4.8	30.1	0.9	32.3	22.7
Oxa_1-B3_A	-6.2	-2.4	3.9	1.9	4.3	0.5	1.0	4.3	17.9	1.1	20.3	11.7
Oxa_1-B3_B	-7.1	-1.6	5.5	2.8	4.4	0.4	1.4	4.4	26.5	0.8	28.1	19.3
Oxa_1-B3_C	-7.1	-2.2	4.9	2.5	4.7	0.4	1.2	4.7	27.0	1.0	29.2	19.8
Oxa_1-B3_D	-7.3	-1.9	5.4	2.7	4.6	0.4	1.4	4.6	28.6	0.9	30.5	21.3
Oxa_2-B3_A	-7.5	-2.0	5.5	2.8	4.7	0.4	1.4	4.7	30.6	0.9	32.5	23.1
Oxa_2-B3_B	-6.7	-2.4	4.3	2.2	4.5	0.5	1.1	4.5	22.2	1.1	24.5	15.5
Oxa_2-B3_C	-7.5	-2.3	5.1	2.6	4.9	0.4	1.3	4.9	30.9	1.0	33.2	23.4
Oxa_2-B3_D	-7.4	-2.1	5.4	2.7	4.7	0.4	1.3	4.7	30.0	0.9	32.0	22.6
Car_1-A	-6.8	-1.5	5.3	2.7	4.2	0.4	1.3	4.2	22.8	0.8	24.3	16.0
Car_1-G	-6.7	-1.3	5.4	2.7	4.0	0.4	1.4	4.0	21.6	0.7	22.9	14.9
Car_2-A	-6.3	-0.9	5.4	2.7	3.6	0.4	1.4	3.6	17.5	0.7	18.4	11.2
Car_2-G	-6.3	-0.8	5.5	2.8	3.6	0.4	1.4	3.6	17.3	0.6	18.1	11.0
Cis_1-A	-6.7	-1.3	5.4	2.7	4.0	0.4	1.4	4.0	21.6	0.7	22.9	14.9
Cis_1-G	-6.4	-1.2	5.2	2.6	3.8	0.4	1.3	3.8	18.8	0.7	20.0	12.4
Cis_2-A	-6.7	-1.5	5.2	2.6	4.1	0.4	1.3	4.1	21.9	0.8	23.4	15.2
Cis_2-G	-6.6	-1.3	5.3	2.7	4.0	0.4	1.3	4.0	20.7	0.7	22.0	14.1
Oxa_1-A	-6.7	-1.3	5.4	2.7	4.0	0.4	1.4	4.0	21.6	0.7	22.9	14.9
Oxa_1-G	-6.2	-0.9	5.3	2.7	3.6	0.4	1.3	3.6	16.7	0.7	17.6	10.5
Oxa_2-A	-6.8	-1.5	5.3	2.7	4.2	0.4	1.3	4.2	22.8	0.8	24.3	16.0
Oxa_2-G	-6.6	-1.4	5.2	2.6	4.0	0.4	1.3	4.0	20.8	0.8	22.2	14.2

Table S2. *HOMO–LUMO energies (in eV) and parameters describing the chemical behaviour of the studied molecules, including the energy gap (ΔE_{gap}), absolute electronegativity (χ), chemical potential (μ), absolute hardness (η), absolute softness (σ), global electrophilicity index (ω), global softness (S), and the maximum additional electronic charge (ΔNmax), calculated at the B3LYP/6-31G(d,p)/LANL2DZ level of theory. The calculated parameters correspond to the complexation reactions between the primary and secondary hydrolysis products of platinum-based drugs: Cisplatin (Cis_1 and Cis_2), Carboplatin (Car_1 and Car_2), and Oxaliplatin (Oxa_1 and Oxa_2) and the tested ligands. The ligands include pyridine derivatives: Nicotinic acid (B3_A), Nicotinamide (B3_B), Isonicotinic acid (B3_C), and Picolinic acid (B3_D), as well as nucleobases: Adenine (A) and Guanine (G). All calculations were performed using the MN15/def2-TZV computational method.*

						MN15/def2-TZVP						
**STRUCTURE**	**HOMO (eV)**	**LUMO (eV)**	**Energy gap (eV)**	**η (eV)**	**μ**	**σ**	**s**	**χ**	**ω**	**ΔNmax**	**ΔEn**	**Δee**
B3_A	-7.6	-1.4	6.2	3.1	4.5	0.3	1.5	4.5	31.6	0.7	33.1	24.0
B3_B	-7.3	-1.1	6.2	3.1	4.2	0.3	1.6	4.2	27.7	0.7	28.8	20.3
B3_C	-7.6	-1.7	6.0	3.0	4.7	0.3	1.5	4.7	32.4	0.8	34.0	24.7
B3_D	-7.7	-1.5	6.3	3.1	4.6	0.3	1.6	4.6	33.4	0.7	34.9	25.6
Adenine (A)	-6.3	-0.4	5.9	3.0	3.3	0.3	1.5	3.3	16.5	0.6	16.9	10.2
Guanine (G)	-6.0	0.0	5.9	3.0	3.0	0.3	1.5	3.0	13.2	0.5	13.2	7.3
Car_1	-7.9	-3.4	4.5	2.3	5.7	0.4	1.1	5.7	35.9	1.3	39.3	28.0
Car_2	-7.0	-2.7	4.3	2.2	4.9	0.5	1.1	4.9	25.3	1.1	28.0	18.3
Cis_1	-7.5	-3.2	4.3	2.2	5.4	0.5	1.1	5.4	30.8	1.2	34.0	23.3
Cis_2	-7.9	-3.4	4.5	2.3	5.7	0.4	1.1	5.7	35.9	1.3	39.3	28.0
Oxa_1	-7.5	-3.2	4.3	2.2	5.4	0.5	1.1	5.4	30.8	1.2	34.0	23.3
Oxa_2	-7.8	-3.3	4.5	2.3	5.6	0.4	1.1	5.6	34.7	1.2	38.0	26.9
Car_1-B3_A	-7.5	-2.0	5.6	2.8	4.7	0.4	1.4	4.7	31.3	0.9	33.3	23.8
Car_1-B3_B	-7.5	-1.8	5.8	2.9	4.6	0.3	1.4	4.6	31.3	0.8	33.1	23.8
Car_1-B3_C	-7.6	-2.4	5.2	2.6	5.0	0.4	1.3	5.0	32.2	1.0	34.5	24.6
Car_1-B3_D	-7.5	-2.1	5.4	2.7	4.8	0.4	1.4	4.8	31.0	0.9	33.1	23.5
Car_2-B3_A	-6.5	-1.8	4.7	2.3	4.2	0.4	1.2	4.2	20.2	0.9	22.1	13.8
Car_2-B3_B	-6.5	-1.6	4.9	2.4	4.0	0.4	1.2	4.0	20.0	0.8	21.6	13.5
Car_2-B3_C	-6.4	-2.2	4.2	2.1	4.3	0.5	1.1	4.3	19.6	1.0	21.8	13.2
Car_2-B3_D	-6.3	-1.8	4.5	2.3	4.1	0.4	1.1	4.1	18.7	0.9	20.5	12.4
Cis_1-B3_A	-7.2	-1.9	5.3	2.7	4.5	0.4	1.3	4.5	27.2	0.9	29.1	20.0
Cis_1-B3_B	-7.2	-1.7	5.5	2.8	4.4	0.4	1.4	4.4	27.0	0.8	28.6	19.8
Cis_1-B3_C	-7.2	-2.3	4.9	2.5	4.7	0.4	1.2	4.7	27.5	1.0	29.7	20.3
Cis_1-B3_D	-7.1	-1.9	5.2	2.6	4.5	0.4	1.3	4.5	26.6	0.9	28.6	19.5
Cis_2-B3_A	-7.5	-2.0	5.5	2.8	4.8	0.4	1.4	4.8	31.3	0.9	33.3	23.8
Cis_2-B3_B	-7.5	-1.8	5.7	2.9	4.7	0.3	1.4	4.7	31.2	0.8	33.0	23.7
Cis_2-B3_C	-7.6	-2.4	5.2	2.6	5.0	0.4	1.3	5.0	32.2	1.0	34.6	24.6
Cis_2-B3_D	-7.4	-2.1	5.3	2.6	4.8	0.4	1.3	4.8	30.1	0.9	32.3	22.7
Oxa_1-B3_A	-6.2	-2.4	3.9	1.9	4.3	0.5	1.0	4.3	17.9	1.1	20.3	11.7
Oxa_1-B3_B	-7.1	-1.6	5.5	2.8	4.4	0.4	1.4	4.4	26.5	0.8	28.1	19.3
Oxa_1-B3_C	-7.1	-2.2	4.9	2.5	4.7	0.4	1.2	4.7	27.0	1.0	29.2	19.8
Oxa_1-B3_D	-7.3	-1.9	5.4	2.7	4.6	0.4	1.4	4.6	28.6	0.9	30.5	21.3
Oxa_2-B3_A	-7.5	-2.0	5.5	2.8	4.7	0.4	1.4	4.7	30.6	0.9	32.5	23.1
Oxa_2-B3_B	-6.7	-2.4	4.3	2.2	4.5	0.5	1.1	4.5	22.2	1.1	24.5	15.5
Oxa_2-B3_C	-7.5	-2.3	5.1	2.6	4.9	0.4	1.3	4.9	30.9	1.0	33.2	23.4
Oxa_2-B3_D	-7.4	-2.1	5.4	2.7	4.7	0.4	1.3	4.7	30.0	0.9	32.0	22.6
Car_1-A	-6.7	-1.1	5.6	2.8	3.9	0.4	1.4	3.9	21.3	0.7	22.4	14.6
Car_1-G	-6.6	-1.1	5.5	2.8	3.9	0.4	1.4	3.9	20.4	0.7	21.5	13.8
Car_2-A	-6.5	-0.9	5.6	2.8	3.7	0.4	1.4	3.7	19.2	0.7	20.1	12.7
Car_2-G	-6.3	-0.7	5.6	2.8	3.5	0.4	1.4	3.5	17.2	0.6	17.9	10.9
Cis_1-A	-6.6	-0.9	5.7	2.9	3.8	0.4	1.4	3.8	20.0	0.7	20.9	13.4
Cis_1-G	-6.4	-0.8	5.6	2.8	3.6	0.4	1.4	3.6	18.1	0.6	18.9	11.7
Cis_2-A	-6.7	-1.1	5.6	2.8	3.9	0.4	1.4	3.9	21.3	0.7	22.4	14.6
Cis_2-G	-6.5	-1	5.5	2.8	3.8	0.4	1.4	3.8	19.3	0.7	20.3	12.8
Oxa_1-A	-6.6	-0.9	5.7	2.9	3.8	0.4	1.4	3.8	20.0	0.7	20.9	13.4
Oxa_1-G	-6.4	-0.7	5.7	2.9	3.6	0.4	1.4	3.6	18.0	0.6	18.7	11.6

Oxa_2-A	-6.7	-1.1	5.6	2.8	3.9	0.4	1.4	3.9	21.3	0.7	22.4	14.6
Oxa_2-G	-6.6	-1.1	5.5	2.8	3.9	0.4	1.4	3.9	20.4	0.7	21.5	13.8
